# Cryo‐EM structure of antibacterial efflux transporter QacA from *Staphylococcus aureus* reveals a novel extracellular loop with allosteric role

**DOI:** 10.15252/embj.2023113418

**Published:** 2023-07-17

**Authors:** Puja Majumder, Shahbaz Ahmed, Pragya Ahuja, Arunabh Athreya, Rakesh Ranjan, Aravind Penmatsa

**Affiliations:** ^1^ Molecular Biophysics Unit Indian Institute of Science Bangalore India; ^2^ ICAR‐National Research Centre on Camel Jorbeer Bikaner India; ^3^ Present address: Memorial‐Sloan Kettering Cancer Center New York NY USA; ^4^ Present address: St. Jude Children's Research Hospital Memphis TN USA

**Keywords:** antibacterial efflux, drug:proton antiport, Indian camelid antibodies, QacA, Rocker‐switch, Membranes & Trafficking, Microbiology, Virology & Host Pathogen Interaction, Structural Biology

## Abstract

Efflux of antibacterial compounds is a major mechanism for developing antimicrobial resistance. In the Gram‐positive pathogen *Staphylococcus aureus*, QacA, a 14 transmembrane helix containing major facilitator superfamily antiporter, mediates proton‐coupled efflux of mono and divalent cationic antibacterial compounds. In this study, we report the cryo‐EM structure of QacA, with a single mutation D411N that improves homogeneity and retains efflux activity against divalent cationic compounds like dequalinium and chlorhexidine. The structure of substrate‐free QacA, complexed to two single‐domain camelid antibodies, was elucidated to a resolution of 3.6 Å. The structure displays an outward‐open conformation with an extracellular helical hairpin loop (EL7) between transmembrane helices 13 and 14, which is conserved in a subset of DHA2 transporters. Removal of the EL7 hairpin loop or disrupting the interface formed between EL7 and EL1 compromises efflux activity. Chimeric constructs of QacA with a helical hairpin and EL1 grafted from other DHA2 members, LfrA and SmvA, restore activity in the EL7 deleted QacA revealing the allosteric and vital role of EL7 hairpin in antibacterial efflux in QacA and related members.

## Graphical abstract

Outward‐open state cryo‐EM structure of drug:proton antiporter QacA uncovers conserved extracellular α‐helical hairpin loop with allosteric function in antibacterial compound efflux.

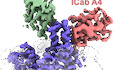

## Introduction

Pathogenic bacteria gain antimicrobial resistance (AMR) through distinct mechanisms to counter the biocidal effects of antibiotics and antibacterial compounds. A major route of developing AMR is through the process of active efflux of biocides, toxic to the pathogen (Blair *et al*, 2015). A diverse array of pumps and transporters is employed by drug‐resistant bacteria to perform antibacterial efflux (Piddock, 2006; Chitsaz & Brown, 2017). Besides being involved in antibacterial resistance through direct efflux, efflux pumps and transporters are known to enhance the persistence among bacterial populations carrying their genes (Pu *et al*, 2016). A number of drug‐resistant pathogens express a variety of efflux pumps in their membranes that include the major facilitator superfamily (MFS), multidrug and toxic compound extrusion family (MATE), small multidrug resistance (SMR), ATP‐binding cassette (ABC), resistance‐nodulation‐division (RND), and proteobacterial antimicrobial compound efflux (PACE) family transporters (Chitsaz & Brown, 2017).

The Gram‐positive pathogen, *Staphylococcus aureus*, is annotated as a high‐priority pathogen by the WHO (2015), which causes localized or systemic infections, including bacteremia, endocarditis, and implant infections (Tong *et al*, 2015). Drug‐resistant strains of *Staphylococcus aureus* express multiple MFS antiporters in their membranes, including the chromosomally encoded transporters NorA, NorB, and NorC that provide resistance against fluoroquinolones (Costa *et al*, 2013), and QacA and QacB that are plasmid‐encoded and are involved in antibacterial efflux (Paulsen *et al*, 1996; Dashtbani‐Roozbehani & Brown, 2021). QacA is highly prevalent in *S. aureus* strains resistant to cationic antibacterial compounds, particularly among those associated with nosocomial infections (Ho *et al*, 2012). The MFS transporters involved in drug:proton antiport (DHA) are divided into DHA1 and DHA2 depending on the number of transmembrane (TM) helices (12 or 14, respectively) in each transporter (Reddy *et al*, 2012). While DHA1 transporters like NorA, MdfA and LmrP comprise 12 TM helices (Heng *et al*, 2015; Drew *et al*, 2021; Brawley *et al*, 2022), QacA, LfrA and SmvA comprise 14 TM helices and are part of the DHA2 family (Brown & Skurray, 2001; Li *et al*, 2004; Dashtbani‐Roozbehani & Brown, 2021).

QacA is a prototypical DHA2 member and is the first proton‐coupled antibacterial efflux transporter to be functionally characterized (Tennent *et al*, 1989). Its expression is regulated by the repressor, QacR, which acts as a negative regulator and allows *qacA* transcription in response to interactions with quaternary ammonium compounds (QACs; Grkovic *et al*, 1998; Schumacher *et al*, 2002). QacA is documented to transport nearly 30 different cationic antibacterial compounds (Mitchell *et al*, 1999). Its efflux ability encompasses divalent and monovalent cationic compounds, including commonly used antibacterials like benzalkonium chloride, dequalinium (Dq), and chlorhexidine (Ch) (Mitchell *et al*, 1999). A homology model of QacA displays six acidic residues (D34, D61, D323, E406, E407, and D411) within the solvent‐accessible vestibule of the transporter that plays diverse roles in the promiscuous antibacterial efflux properties of QacA (Majumder *et al*, 2019). The experimentally determined topology of QacA depicts the presence of a large extracellular loop between TMs 13 and 14, whose role remains unexplored. QacA also retains conserved sequence motifs A, B, and C that are characteristic of DHA and other MFS transporters (Rouch *et al*, 1990; Zhang *et al*, 2015).

This study delves into cryo‐EM structure elucidation of QacA using a single mutant of D411N that enhances its homogeneity. We employ two Indian camelid antibodies (ICabs) as fiducial markers to obtain the first high‐resolution structure of QacA in an outward‐open state (QacA_oo_), at a resolution of 3.6 Å. The structure provides insights into the positions of the protonatable acidic residues around the solvent‐accessible vestibule. It identifies the extracellular loop 7 as a structured α‐helical hairpin loop motif that connects elongated helices, TMs 13 and 14 in QacA. The motif is conserved in a subset of DHA2 transporters and removal of the hairpin completely compromises efflux. Based on molecular dynamics (MD) simulation trajectories, we predict that this motif interacts with extracellular loop 1 (EL1) on the N‐terminal domain, where substitutions at the interfacial residues between EL7 and EL1 lead to compromised efflux. We could recover activity in the EL7 deletion construct of QacA by creating chimeras of EL7 and EL1 from related DHA2 members, LfrA and SmvA, thereby reinforcing the importance of the formation of this extracellular gate between EL7 and EL1 for effective QacA activity.

## Results

### Construct optimization to enhance QacA homogeneity

**Figure 1:**
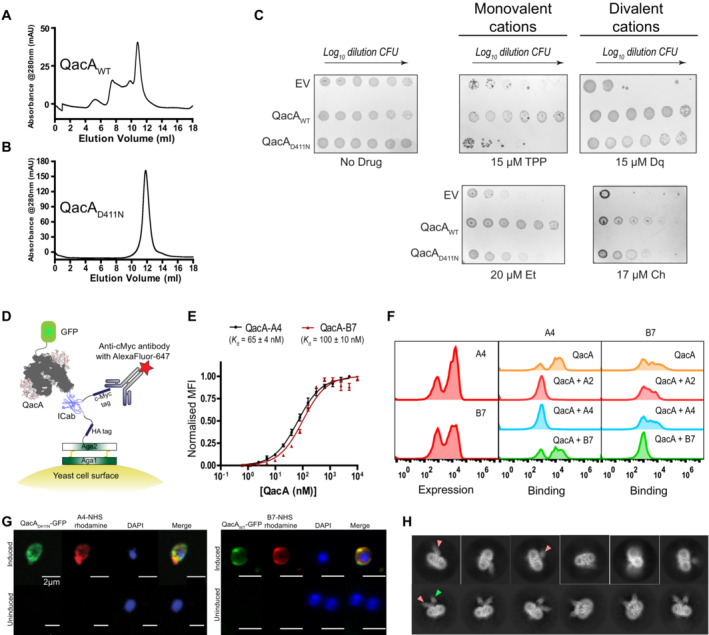
Generation of ICabs against QacA and their characterization A, B
Size exclusion chromatography profiles of QacA_WT_ and QacA_D411N_ mutant, where the latter shows a more homogenous profile than QacA_WT_.C
Survival assays against substrates of QacA showed loss of activity against monovalent cationic antibacterials (Et, ethidium; TPP, tetraphenylphosphonium), while still retaining partial activity against divalent cations (Ch, Chlorhexidine; Dq, Dequalinium). EV, pBAD empty vector. *n* = 3 for biological replicates. CFU indicates colony‐forming units. Image color was inverted from black to white background to enhance clarity.D
Schematic of yeast surface display platform used to enrich anti‐QacA‐ICabs from the cDNA library generated from camels immunized against QacA_D411N_. QacA was fused to GFP while ICab was expressed with HA and c‐Myc tags, probed by AlexaFluor‐647 conjugated antibody.E
Population shift‐based titration of QacA‐GFP against ICabs yielded an apparent *K*_d_ of 65 ± 4 nM for QacA‐A4 complex and 100 ± 10 nM for QacA‐B7. *n* = 3 for independent replicates. Error bars denote S.E.M., and each data point represents mean value.F
ICab binding analysis performed through FACS using yeast populations displaying A4 ICab could bind to precomplexed purified ICab B7 and QacA‐GFP and vice versa, indicating that these ICabs do not compete for a common epitope and bind at different sites. Representational single set is shown out of three independent replicates.G
Colocalization of QacA_WT_ or QacA_D411N_ (tagged with GFP) on *E. coli* spheroplasts (stained with DAPI for viability) with ICabs (labeled with NHS‐Rhodamine) was screened against ICab A4 (left) and ICab B7 (right). *n* = 10–30 spheroplasts were imaged for each sample.H
Representative reference‐free 2D classes of QacA_D411N_ embedded in UDM micelle, in complex with ICab A4 (top) and with ICabs A4 and B7 (bottom), pointed with pink and green arrows, respectively. Representative classes shown for QacA‐A4 complex are from a set of 68,000 particles and QacA‐A4/B7 complex are from 218,040 particles. Source data are available online for this figure.

The QacA_WT_ was heterologously expressed and isolated from the membranes of an *E. coli* strain lacking the efflux pumps *acrB*, *mdfA*, and *ydhE*. Despite optimizing detergent conditions and identifying undecyl–β‐D‐maltopyranoside (UDM) as the optimal detergent for purification, the QacA_WT_ construct displays significant levels of aggregation in size exclusion chromatography (Fig 1A). As part of the analysis of protonation sites within QacA that affect substrate efflux, we identified QacA_D411N_ as a mutant that significantly improves the protein's homogeneity to facilitate structural studies (Fig 1B). We observed in our earlier studies that D411 is essential for the transport of monovalent cations like ethidium (Et) and tetraphenyl phosphonium (TPP), yet QacA_D411N_ retains the ability to transport some divalent cationic substrates like Ch and Dq, which are popular antibacterial compounds (Fig 1C; Majumder *et al*, 2019). Conformational stabilization through cytosolic rim residue mutagenesis had been successfully attempted in the case of MdfA (Zomot *et al*, 2018); hence, we sought to further stabilize QacA_D411N_ by introducing additional mutants of polar residues at the cytosolic membrane interface, including E137Q (TM4‐TM5), E141Q, R142Q and A143N (TM5), D267N (TM8‐TM9), L389N and L392N (TM12; Appendix Fig S1A). Of these, QacA_D411N/E137Q_ displayed a mildly weakened efflux, suggesting a lowered ability to cycle through conformational transitions (Appendix Fig S1B). The construct was purified to homogeneity (Appendix Fig S1C) and used as the antigen for immunizing camels to raise single‐domain camelid antibodies.

### Yeast display screening allows ICab isolation against QacA

**Figure EV1:**
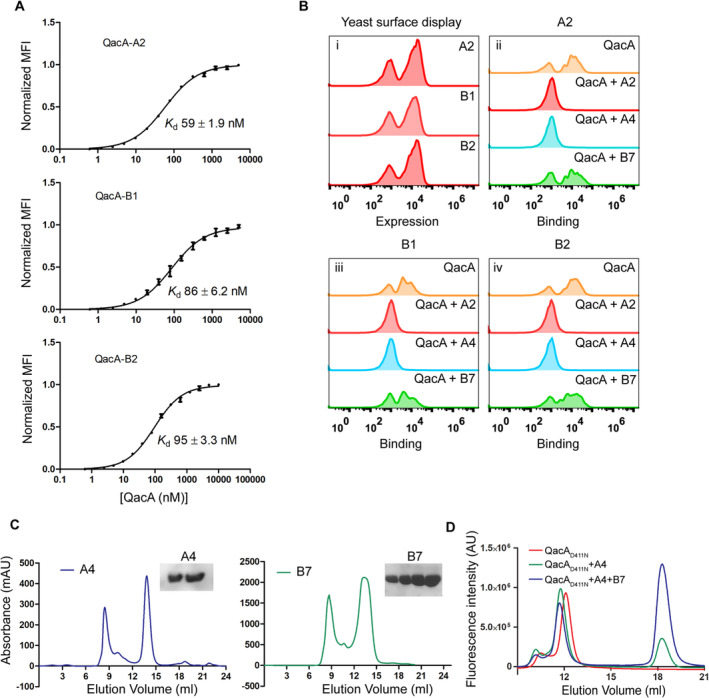
Characterization of affinities of ICabs against QacA A
Flow cytometry derived apparent affinity measurements of ICabs A2 (top), B1 (middle) and B2 (bottom) for QacA. Error bars represent S.E.M. with *n* = 3 for technical triplicates.B
Population shift‐based titrations of ICabs against QacA‐GFP preincubated with other ICabs in the group. A rightward shift of population infers noncompetitive binding of ICabs.C
SEC profiles of ICabs A4 (left) and B7 (right). Collected fractions around 13–14 ml of elution volume were run on SDS–PAGE and the corresponding bands are shown (inset).D
FSEC profiles with leftward shifts in elution volumes of QacA_D411N_ in the presence of A4 and B7 indicate increase in apparent molecular mass of QacA upon binding with either or both the ICabs. Source data are available online for this figure.

Single‐domain Indian camelid antibodies (ICabs) against QacA were screened through a yeast surface display platform using a previously optimized protocol (described in Material and Methods; Kumar *et al*, 2020). ICabs were sorted for expression and affinity for QacA using FACS (Fig 1D; Appendix Fig S2A). Among the pool of binders, we found five unique clones that could induce population shifts in the range of 65–97% upon interacting with QacA_D411N_‐GFP (Appendix Fig S2B and C). A further analysis through QacA_D411N_ titration in flow cytometry yielded similar apparent dissociation constants ranging from 59 nM to 100 nM for the identified ICabs (Figs 1E and EV1A).

We also evaluated whether the ICabs competed for a common epitope on QacA through flow cytometry analyses. QacA_D411N_‐GFP was first incubated with a purified ICab and then screened for binding against yeast cells expressing other ICabs. In a situation involving a common epitope between the purified antibody and the competing antibody expressed on the yeast cell surface, we expect no shifts in the population as the purified antibody prevents interactions by the latter. In the scenario where a purified antibody binds to an alternate site that is distinct from the binding site of the surface‐displayed antibody, the antigen (QacA_D411N_‐GFP) would interact despite the presence of the bound antibody. Through these analyses, we could establish that four among the five ICabs, including A2, A4, B1, and B2, interact competitively at the same epitope, whereas B7 shifts the population of cells despite having a bound antibody, suggesting interactions at an alternate epitope in a noncompetitive fashion (Figs 1F and EV1B). Similar results were obtained when ICabs A4 and B7 were heterologously purified, incubated with QacA_D411N_, and analyzed on FSEC (Fig EV1C and D). These ICabs were also analyzed for topological localization using spheroplasts of *E. coli* expressing QacA‐GFP, where we observed that both A4 and B7 bind to the extracellular surface of QacA (Fig 1G). We employed these ICabs to perform cryo‐electron microscopy (cryo‐EM) on QacA_D411N_ complexed with A4 and B7 with a combined mass of ~ 80 kDa, excluding the detergent micelle. The reference‐free 2D classes computed with the heterodimeric A4‐QacA complex and the heterotrimeric A4/B7‐QacA complex indicate their interactions on the extracellular face of QacA with independent albeit spatially proximal epitopes (Fig 1H).

### Cryo‐EM structure of QacA reveals an outward‐open conformation

**Figure 2:**
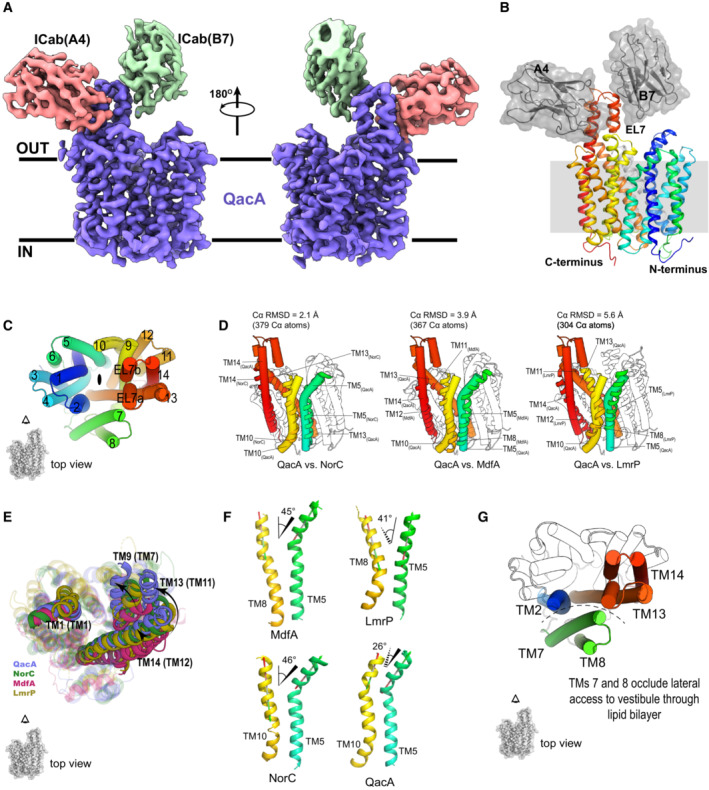
Architecture of QacA‐A4‐B7 complex and comparison with DHA2 antiporters A
Cryo‐EM model of QacA (blue) in complex with ICab A4 (pink) and ICab B7 (green; Dataset EV1).B
Cartoon representation of the complex, with 14 transmembrane helices of QacA shown in rainbow gradient from N terminus to C terminus. Extracellular loop 7 (EL7), with which B7 interacts, lies between elongated TMs 13 and 14 that protrude toward the periplasmic side of the membrane and form the epitope which interacts with A4.C
Topology of transmembrane helical arrangement, the top view shows canonical MFS fold of the transporter, along with the location of EL7 segment as seen in the top view.D
Side views of structural alignments of QacA (cylindrical helices) with LmrP (PDBID: 6T1Z), MdfA (PDBID: 6GV1), and NorC (PDBID: 7D5P) with Cα RMSDs written above the alignments. TMs 5 (green), 10 (yellow, TM 8 in MdfA and LmrP), 13 (orange, TM 11 in MdfA and LmrP), and 14 (red, TM 12 in MdfA and LmrP) are highlighted to show structural differences between QacA and representative DHA transporters. EL7 of QacA is also shown as cylindrical helices.E
Structural alignment of QacA_D411N_ (blue) with MdfA (pink, PDBID: 6GV1), LmrP (yellow, PDBID: 6T1Z), and NorC (green, PDBID: 7D5P). Some of the major rearrangements in TM assembly are shown as opaque helices (TMs 9, 13, and 14 in DHA2 transporters QacA and NorC) with their corresponding TMs in DHA1 transporters MdfA and LmrP are labeled in parentheses. TM1 is shown to suggest a stationary point of view for these rearrangements.F
Angular separation between the upper halves of TM 5 and TM 10 in QacA and NorC, and of TM5 and TM8 in MdfA and LmrP. Dashed lines represent helices going into the plane to the tapered end, while solid line represents helices on the plane (uniform line) or coming out of the plane toward the broader end.G
QacA TM helices shown as cylinders from the top to highlight block of lateral access of the vestibule to the lipid bilayer (dashed arc) due to the presence of TMs 7 and 8 (green). TMs 2, 13 and 14 are shown in their usual color scheme.

The cryo‐EM structure of QacA_D411N_ in UDM micelles was determined in complex with two ICabs (A4 and B7) that served as fiducial markers during cryo‐EM image processing (Appendix Fig S3A and B). The 2D classes of the particle set used for the final reconstruction displayed the presence of secondary structural elements within the transporter, indicating well‐aligned particles with multiple orientations (Appendix Fig S3C). The models of QacA and two ICabs were built into the refined map that was density modified (Appendix Fig S3D and E, Table S1) and yielded unambiguous densities for nearly the entire transporter, from residues 5 to 514. The density was visible for all the 14 TM helices and intra‐ and extracellular loops (Fig 2A; Appendix Fig S4A and B, Dataset EV1). ICabs A4 and B7 interact closely with the extracellular loop 7 (described later). The organization of the TM helices is typical of an MFS fold transporter where two six‐helical bundles (TMs 1‐6 and TMs 9‐14) are arranged with a pseudo‐twofold symmetry (Fig 2B and C). The TMs 7 and 8 are arranged as an insert in the long loop joining the two bundles and are juxtaposed next to the gap between TM2 and TM13 of QacA (Fig 2C). This arrangement of TM helices is also observed in other 14 TM containing MFS transporters, including proton‐dependent oligopeptide symporters (POTs) and NorC, a putative DHA2 member (Martinez Molledo *et al*, 2018; Kumar *et al*, 2021). While the organization of helices is consistent with the MFS fold, the helical positions in QacA differ in comparison with the structures of DHA1 members like MdfA and LmrP (Fig 2D). The differences are particularly apparent in the organization of TMs 5, 9, 10, 13 and 14. The architecture of the QacA_D411N_ structure is outward‐open with solvent accessibility toward the extracellular side. The solvent‐accessible vestibule is narrow compared with other DHA1 members, likely due to the discrepancies within helices that are placed relatively closer to the vestibule than LmrP and MdfA (Fig 2E). The interdomain cleft between TMs 5 and 8 in LmrP and MdfA is wide open toward the extracellular side with relative angles of 41° and 45°, respectively, that can allow the interactions of amphipathic molecules like detergents through the bilayer (Fig 2F; Debruycker *et al*, 2020). This cleft is narrow in QacA between TMs 5 and 10 (equivalent to TM8 in DHA1) as both helices are largely parallel, disallowing the membrane environment to access the vestibule. When compared to the inward‐open state of QacA (QacA_io_) AlphaFold2 model, the TM5 undergoes an angular shift away from TM10 that remains unaltered. The movement of TM5 and TM1 at the cytosolic face allows QacA to attain a cytosol‐facing conformation. The cleft formed between TMs 2 and 13 (TM11 in DHA1) widens toward the extracellular face of the transporter. However, exposure of the vestibule to the lipid bilayer in QacA at this interface is occluded by the presence of TMs 7 and 8 that are juxtaposed next to this cleft (Fig 2G), whereas LmrP and MdfA lack this transmembrane insertion (Nagarathinam *et al*, 2018).

### The vestibule of QacA is negatively charged

**Figure 3:**
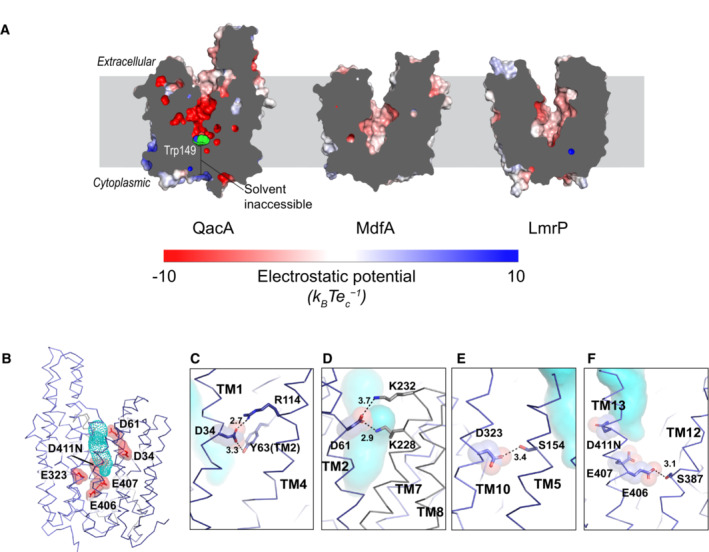
Solvent‐accessible vestibule in the outward‐open state of QacA_D411N_ A
APBS electrostatic maps in solid longitudinal sections of QacA along with common drug proton antiporters. W149 in QacA is shown in green spheres.B
Solvent‐accessible vestibule of QacA in this model (shown with blue dots) is lined with acidic residues, shown in red sticks and spheres.C–F
Neighborhood of all the acidic residues in the vestibule (sticks and transparent spheres; side chains of interactants are shown in sticks) with distances shown in angstroms (Å). Solvent‐accessible volumes are shown with cyan blobs in the background.

The solvent‐accessible vestibule in QacA extends nearly halfway across the bilayer. It is secluded from access to the cytosol through multiple hydrophobic and H‐bond interactions in TM helices below residue Trp149 in TM5, which forms the base of the vestibule (Fig 3A). Like many other transporters of the DHA family, QacA has a negatively charged environment in its vestibule that facilitates the binding and transport of cationic antibacterials. However, as described in previous studies, unlike some well‐characterized members of the DHA1 family like NorA, MdfA, and LmrP that use a single or a couple of acidic residues to drive transport, QacA possesses multiple acidic residues scattered throughout the vestibule, making the overall electrostatic charge inside the vestibule highly negative as observed using the Adaptive Poisson–Boltzmann Solver (APBS) to calculate surface electrostatics among DHA transporters (Fig 3A; Jurrus *et al*, 2018). Although the use of APBS is fraught with some shortcomings like overestimating dielectric at solvated regions with low solvent exchangeability (which also affects the protonation/deprotonation propensity of partially buried residues), the method can be robustly used to visualize the distribution of charged residues in the vestibule. The larger number of acidic residues within the QacA vestibule renders the vestibular surface electronegative to facilitate attraction of protons from the extracellular medium that can be exchanged for cationic antibacterials in the cytosol. This introduces the possibility of localization of metal ions in the vestibule of QacA. Indeed, through MD simulations of QacA in lipid bilayer, we observe an enrichment for both Na^+^ and K^+^ ions but not Cl^−^, at the base of the vestibule, surrounded by E406, E407, and D411 (Appendix Fig S5A). To assess whether Na^+^ can induce proton efflux in QacA, we performed everted vesicle assay with various concentrations of sodium gluconate and found that QacA was not able to transport sodium ions in comparison to empty vesicles lacking QacA (Appendix Fig S5B). Moreover, in the presence of Na^+^, QacA retained the ability of TPP to induce proton efflux (Appendix Fig S5B). The interactions of Na^+^ or K^+^ ions are likely to have a very low affinity as we had previously established that ~ 1,000 times lower concentrations of cationic antibacterial compounds can compete for and release protons in purified QacA despite the presence of 200 mM NaCl (Majumder *et al*, 2019).

The acidic residues in QacA's vestibule have been categorized into three groups based on their contributions to substrate binding and transport (Majumder *et al*, 2019). While D34 and E407 have been deemed essential to QacA's function for all types of substrates, residues like D411 and D323 are shown to play conditional roles in transporting specific divalent cationic substrates with longer linkers. On the contrary, while providing partial fitness to transport substrates like ethidium and TPP, residues like D61 are considered nonessential for multiple substrates. E407 has also been attributed to having a moonlighting role of substrate recognition in the case of dequalinium while typically acting like a protonation site during the transport of TPP and pentamidine. A homology‐based QacA_io_ model was earlier used to provide possible explanations to substrate binding and transport, and cysteine crosslinking‐based assays of QacA mutants were used to verify relative solvent accessibilities of these residues (Majumder *et al*, 2019). The QacA structure described in our present study provides insights into the environment of the protonation sites in its outward‐open state (Fig 3B).

The acidic residues in the vestibule of QacA are involved in myriad polar interactions with neighboring residues through their side chains. D34 (TM1) interacts with a nearby R114 (TM4) and Y63 (TM2; Fig 3C). Y63 is conserved across transporters of the DHA2 subfamily (Appendix Fig S6) and is essential in the functioning of QacA, where a mutation to valine leads to loss of function in QacA but retains function upon Y63F mutation (Wu *et al*, 2008). This type of interaction is also structurally conserved in the case of MdfA, a DHA1 transporter, where E26 interacts with Y30 (TM1) and Y127 (TM4), albeit only in the outward‐open conformation. Furthermore, the D34 interacts with R114, a highly conserved residue that forms motif B in DHA members. Interaction of motif B arginine with the substrate recognizing acidic residues is suggested to enhance the pKa value and facilitate deprotonation at neutral pH (Zhang *et al*, 2015). On the contrary, D61 (TM2) has only partial conservation among DHA2 transporters (Appendix Fig S6) but forms networks adjoining the vestibule with lysine residues from TM7 (K228 and K232; Fig 3D). This feature is absent in DHA1 transporters due to the lack of corresponding interdomain linker TMs 7 and 8 in them.

While there is no sequence conservation of D323 (TM10) even among DHA1/2 transporters (Appendix Fig S6), it fits within the groove formed by TM5 and TM10 between the N‐terminal and C‐terminal domains and forms a hydrogen bond with the hydroxyl group of S154 (TM5; Fig 3E). D323 is selectively needed to transport certain divalent cations like pentamidine but not others (Paulsen *et al*, 1996; Majumder *et al*, 2019). Lying toward the lower leaflet along the normal of the bilayer, E406 and E407 in TM13 are peculiar in their partial conservation as acidic residues or as asparagines in many DHA2 transporters, which are similar to QacA (Fig 3F, Appendix Fig S6). This is interesting, considering that while E407 has been characterized primarily as a protonation site for most of QacA's substrates, it acts as a substrate recognition site for some of QacA's substrates like Dq and Ch that have cationic moieties separated by long linkers. E407 is exposed to the solvent‐accessible vestibule in QacA and could undergo protonation and deprotonation events during the transport cycle. E406 interacts with the hydroxyl group of S387 (TM12), through a hydrogen bond through its side chain (Fig 3F). Interestingly, substitution of S387 to a cysteine causes an enhanced sensitivity of QacA_S387C_ expressing *E. coli* to divalent cationic antibacterials (Dashtbani‐Roozbehani *et al*, 2023). A single α‐helical turn above the E407 is the residue D411 in TM13. This was the site where a substitution to a neutral amino acid stabilized the conformation and biochemical behavior of QacA. The D411N directly faces the solvent‐accessible vestibule and is likely to undergo protonation or deprotonation during the transport cycle (Fig 3F). Using MD simulations, we demonstrate that protonation of D411 causes conformational transitions from outward‐open to an occluded state of QacA (described later). Upon comparing these residues with the corresponding ones from homologous transporters of the DHA2 family, we observe that many of these residues are conserved (Appendix Fig S6). In addition to the presence of multiple acidic residues, we observe a unique structural feature in the form of an α‐helical hairpin located in extracellular loop 7 (EL7) in QacA (Appendix Fig S6).

### EL7 helical hairpin is the site for ICab interactions

**Figure 4:**
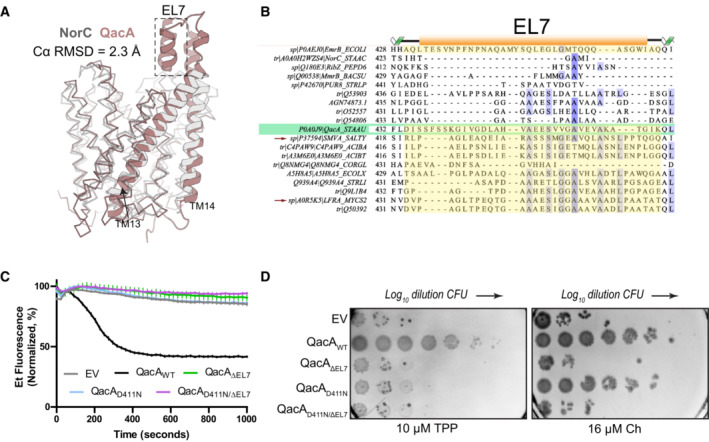
Role of EL7 in antibacterial efflux in QacA A
Alignment of NorC (white) and QacA (brown) structures showing the presence of an α‐helical hairpin in EL7 in QacA (dashed lines).B
Multiple sequence alignment of DHA2 transporter sequences closest to QacA, focused on EL7 region. Highlighted regions (yellow) demarcate EL7 found in a subset of these transporters. Arrows point to two homologs LfrA (*M. smegmatis*) and SmvA (*S. typhimurium*). QacA is highlighted in green.C
Whole cell ethidium efflux assay with overexpressed EL7 deletion construct in the background of wild type and D411N mutant. *n* = 3 for independent replicates, one of which is shown here. Error bars represent SEM from six technical replicates.D
Survival assays of QacA_ΔEL7_ and QacA_D411N/ΔEL7_ mutants in the presence of 10 μM Tetraphenylphosphonium (TPP) and 16 μM Chlorhexidine (Ch). Data from one representative biological replicate are shown here. Image colors were inverted for improved clarity. Source data are available online for this figure.

**Figure EV2:**
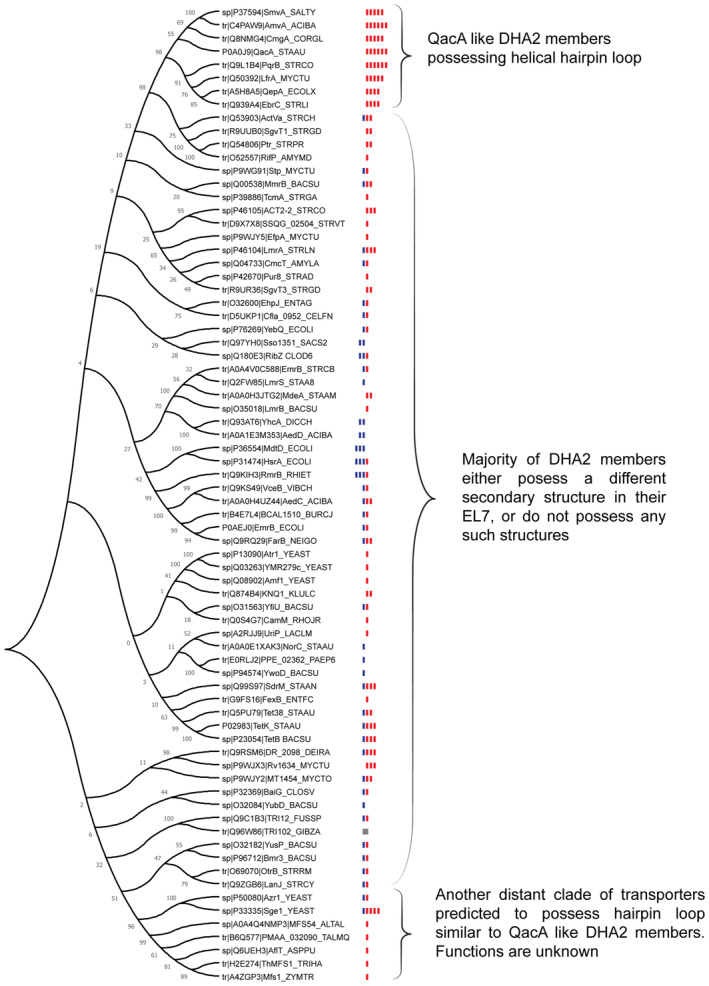
Phylogeny of DHA2 members listed in the Transporter Classification Database Bootstrapped phylogeny derived from an evolutionary tree made using maximum likelihood method. Branching confidence is mentioned at each node. Each red or blue bar represents an acidic or basic residue, respectively, present in the vestibule of the transporter, as manually curated from their AlphaFold2 models.Source data are available online for this figure.

Unlike other DHA1 and DHA2 transporters, QacA TM helices 13 and 14 extend beyond the bilayer to form a highly ordered extracellular loop 7. A comparison of the QacA and NorC structures displays the EL7 as a feature unique to QacA (Fig 4A). A comparison of other canonical DHA2 members related to QacA reveals the insertion of this EL7 hairpin loop as a distinct structural feature (Fig 4B, Expanded view Fig EV2). The residues in the region of 441–467 form a helix‐turn‐helix motif resembling an α‐helical hairpin (EL7a, 7b) which is directed toward the membrane bilayer, but does not plug or occlude the vestibule of QacA in the outward‐open conformation. It is notable that the EL7 helical hairpin sequence is also observed in a few transporters among the functionally characterized DHA2 members like LfrA and SmvA (Takiff *et al*, 1996; Wand *et al*, 2019; Fig 4B; Appendix Fig S6). In the QacA structure, the EL7 is the primary interaction site with two ICabs bound at distinct locations.

**Figure EV3:**
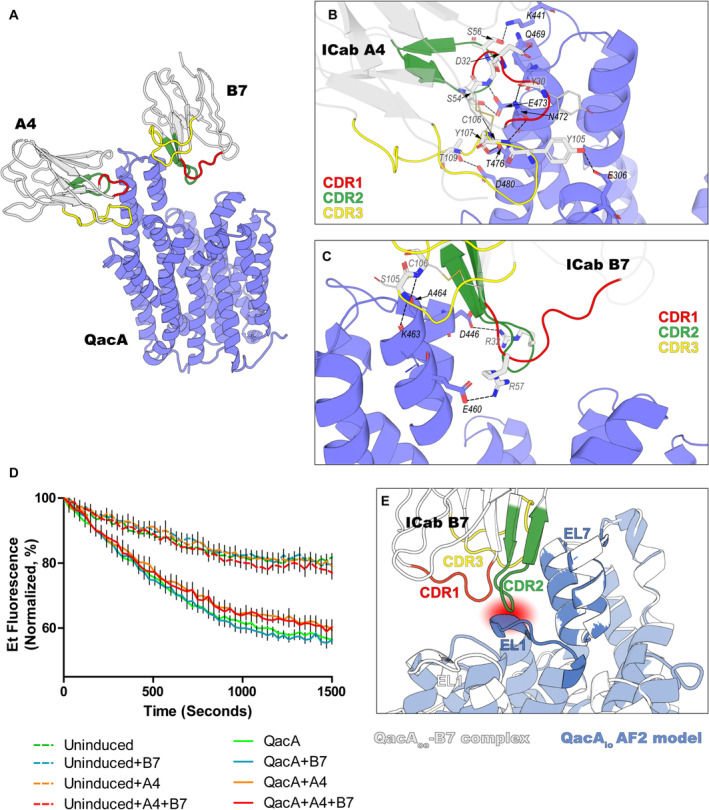
General overview of QacA‐ICab interfaces and their inhibition propensities A
Structural overview of QacA_D411N_‐A4‐B7 complex with the globular domains of ICabs shown as gray cartoons, and the CDRs 1, 2 and 3 are shown in red, green, and yellow colors, respectively. QacA is shown in blue helices.B
QacA‐A4 interface shown with participating residues in sticks. For the residues that are involved in forming polar/H‐bond interactions through their main‐chain atoms only, the sidechains are shown as thin lines, for clarity. Interacting residues from A4 and QacA are labeled in gray and black fonts and depicted in gray and blue sticks, respectively. H‐bond interactions are shown as dotted lines. Color scheme follows panel A.C
QacA‐B7 interface shown in the same convention as panel (B).D
Spheroplasts‐based ethidium efflux assay shown as traces normalized between 100 and 0% to first reading in each sample and zero fluorescence, respectively. Data shown here are from a single biological replicate. Error bars represent SEM obtained from six technical replicates.E
Superposition of QacA_io_ model with QacA_D411N_‐B7 coordinates structurally aligned at the EL7 region. A probable clash between CDR2 (green cartoon) and EL1 (opaque blue cartoon) observed through rigid fitting is shown as the circled ROI. Source data are available online for this figure.

The ICab A4 interacts with helical extensions of TM13 and TM14, and B7 interacts with the hairpin loop of QacA (Fig EV3A–C). The densities of the two ICabs are at a low resolution in some parts of the framework region, but the antigen‐binding loops have well‐defined densities that allowed chain tracing and sidechain assignment. ICab A4 displays a high apparent affinity (*K*_d_ ~ 60 nM) for QacA EL7. This is the epitope where most of the binders interact. The ICab interacts with an interfacial area of 814.2 Å^2^. The epitope interactions are mediated through CDRs 1, 2 and 3, with CDR3 forming the bulk of the interactions and bridging the residues in EL5 (E306, H‐bond with Y105 of CDR3 in ICab A4) with EL7 (Fig EV3B). The CDR3 of A4 is positioned unconventionally as it does not curve over the framework like a typical nanobody but elongates to form the antigen‐binding site. CDRs 1 and 2 have localized interactions with TM13 and TM14 at a region distant from the helical hairpin loop that forms EL7.

The ICab B7 is a conventionally structured camelid antibody and interacts weakly with the hairpin loop. The apparent affinity displayed by B7 is in the similar range (*K*_d_ = ~ 100 nM) when compared to A4, with poorer density in the framework region leading to gaps in the residue assignment in the structure. The B7 has a smaller domain interface of 446.3 Å^2^ compared with A4 with its epitope in QacA. The interactions are mainly modulated through H‐bonds between EL7 hairpin, particularly around EL7b and the residues in CDR loops 1 and 3 (Fig EV3C). We surmised that interactions with B7 or A4 could interfere with QacA's transport properties due to their interactions in the EL7 region. To evaluate this, we employed the use of *E. coli* spheroplasts to perform ethidium efflux assay in the presence of at least ~ 10 times molar excess of ICabs. Since the electron transport chain is already present on the inner membrane of the cell, we proposed that the system would work in spheroplasts as well. Indeed, we could see a steady efflux of ethidium from the spheroplasts with overexpressed QacA in the membrane when glucose was introduced in the medium, albeit significantly slower than with whole cells (Fig EV3D). The efflux from spheroplasts is also sensitive to reserpine, which was earlier proposed as an inhibitor of QacA (Appendix Fig S7A and B; Mitchell *et al*, 1999). Surprisingly, the ability to efflux did not change in the presence of either B7 or A4, suggesting that they do not affect the transport of substrates through QacA (Fig EV3D). The ICab A4 binds to the TM13 and TM14 helices that protrude out of the bilayer and does not hamper interactions required for rocker‐switch motions in QacA. However, B7 interactions that happen at the EL7 hairpin region could have interfered with the transport process. Upon further analyses by overlapping the hairpin loops of the AlphaFold2 QacA_io_ structure on the QacA_oo_, we could observe minor side chain clashes in a part of EL1 region (residues 49–51) with the CDR2 of B7 (residues 55–57) whereas the remaining EL1 and EL7 interface formation remains unhampered (Fig EV3E).

Through multiple sequence alignments and phylogenetic analyses of all the DHA2 members annotated in the transporter classification database, we find that QacA and its closest annotated homologs form a distinct clade of antiporters that possess a highly electronegative surface in their vestibules, and all of these homologs have been predicted to possess the same helical hairpin topology for their EL7 as described for QacA. With the exception of another small branch putatively possessing a similar hairpin topology, none of these transporters have been predicted to possess the secondary structure that QacA displays in its EL7 (Fig EV2). Moreover, almost every DHA2 member with an equally high negative electrostatic vestibular surface is found in the evolutionary clade that harbors QacA. We observe the appearance of a distinct pattern correlating the high density of acidic residues in the vestibule and probable α‐helical hairpin loop of EL7 in DHA2 members.

To analyze the role of the hairpin loop on QacA function, we deleted the residues 443–465 that form the helix–loop–helix motif in EL7 of QacA. Upon deletion, even with comparative expression levels between QacA_WT_ and QacA_ΔEL7_, the latter fails to provide resistance against any of the antibacterial compounds tested (Fig 4C and D).

### EL1‐EL7 interface formation is required for antibacterial efflux

**Figure 5:**
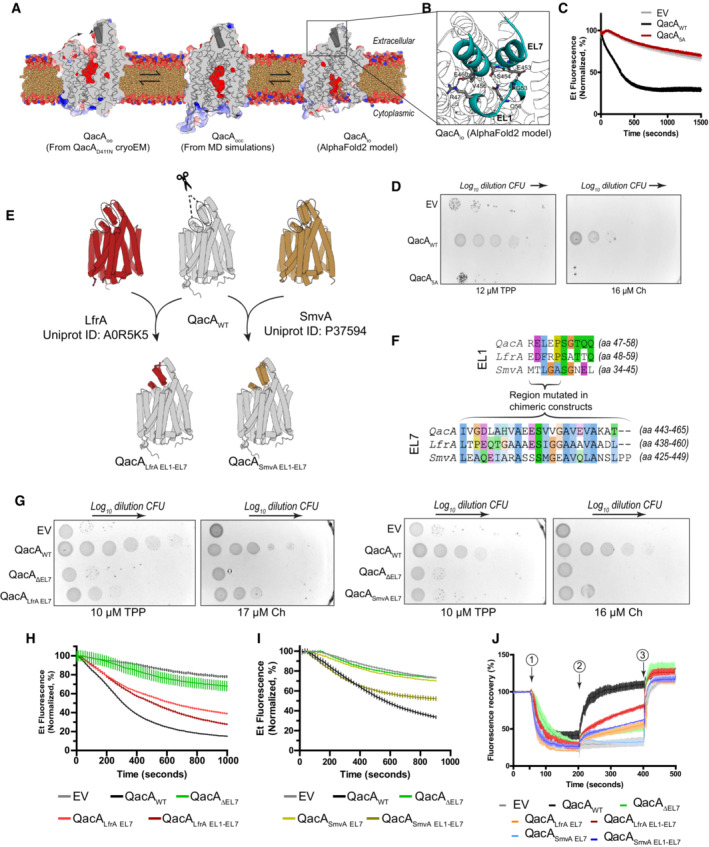
Role of EL1‐EL7 interaction in the transport activity of QacA A
Sagittal section of QacA structure in outward‐open state (QacA_oo_), a frame from MD simulations showing QacA in inward‐facing occluded state (QacA_occ_), and AlphaFold2 model of QacA in an inward‐open state (QacA_io_) shown. APBS electrostatics are used to depict the presence of acidic residues in the vestibule. EL7 region is highlighted in the states as gray cylinders.B
EL1‐EL7 interaction region is shown from the ROI drawn in QacA_io_ model. Residues modeled to be involved in EL1‐EL7 interactions are shown as sticks, with possible polar interactions shown with dashes.C
Whole cell ethidium efflux assay done with *E. coli* JD838 cells expressing QacA_WT_ or QacA_5A_. Empty vector (EV) was used as a control for the experiment. A graph of one independent replicate is shown with error bars representing SEM of six technical replicates.D
A single representative replicate of survival assays shown for the same cells spotted in 10‐fold dilution series on 12 μM TPP and 16 μM Ch. Image colors were inverted for clarity in the figure.E
A schematic of chimeric construct design. AlphaFold2 models of LfrA (red), QacA_io_ (gray) and SmvA (olive) are shown as cartoon cylinders. Chimeric constructs are shown with new stretches colored based on their origin. Only dual chimeras (QacA_LfrA EL1‐EL7_ and QacA_SmvA EL1‐EL7_) are drawn. Dotted lines represent excised regions used to create chimeras.F
Aligned sequences of EL1 and EL7 from QacA, LfrA and SmvA. Indicated regions were replaced in QacA's original sequence. Similar amino acids are highlighted based on clustal color scheme. Sequence limits of the respective stretches are mentioned in parentheses.G
Survival assays of solely EL7 chimeric constructs compared against empty vector (EV) and QacA_WT_. One representative biological replicate is shown. Image color inverted for clarity.H, I
Whole cell‐based ethidium efflux assay done with *E. coli* JD838 cells expressing QacA_WT_, its EL7 deletion construct (QacA_ΔEL7_), and the chimeric mutants of LfrA and SmvA. All assays were done in at least 2 independent replicates. One representative replicate shown with data normalized to highest value in each trace. Error bars depict S.E.M. for six technical replicates.J
Everted vesicles‐based assays of chimeric and EL7 deletion constructs of QacA. Traces represent mean values of three replicates performed on vesicles made with a single batch of cells for each construct. Timepoints indicated as 1–3 above the traces indicate the addition of 100 μM ATP, 1 mM TPP and 2 μM Nigericin, respectively. One representative shown for data obtained from biological triplicates. Error bars represent SEM for technical triplicates from one of three biological replicates. Individual traces are shown in Expanded view Fig EV4B. Source data are available online for this figure.

To find out the possible role of EL7 in the transport cycle of QacA, we ran MD simulations to track the dynamics of EL7 with respect to the rest of the transporter. We converted the D411N substitution back to aspartate and set up simulation boxes with the D411 in protonated and deprotonated states. Every setup was run with different random seeds for 1 μs of simulation time each, and the trajectories were analyzed for conformational changes by tracking the distance between the centers of masses of EL1 and EL7 residues' Cα atoms, and by tracking the angle between the two rocker‐switch bundles (Appendix Fig S8A). While all other acidic residues were modeled unprotonated in the setups, we found that mere protonation of D411 caused the transporter to transition toward the occluded state (Appendix Fig S8B). We also predicted the inward‐open state, QacA_io_, using AlphaFold2, which is known to model transporter structures with high accuracy (Fig 5A and B; Del Alamo *et al*, 2021; Del Alamo *et al*, 2022). Analyses of the occluded and inward‐open states revealed the appearance of interactions between residues of EL7 and extracellular loop 1 (EL1) present between TMs 1 and 2 in the N‐terminal domain, possibly involved in gating the vestibule from the extracellular end (Fig 5B). In DHA1 transporters like MdfA the corresponding loop (EL6) between TM11 and TM12 has no role in gating, as it is distant from the vestibule (Heng *et al*, 2015). While not all the interacting residue pairs were found to be common to both QacA_occ_ and QacA_io_, we decided to check whether these residues R47, Q56 (EL1), and S454, V456, and E460 (EL7) have any role in regulating transport activity in QacA.

We found that while the activities vary to a minor extent among mutants with comparable expression levels (Appendix Fig S9A and B), the efflux activity remains unaltered for individual substitutions at the EL1‐EL7 interface of QacA (Appendix Fig S10A–E). However, we observe that wider substitutions at this interface through mutations of five residues (R47A/Q56A/S454A/V456A/E460A, named as QacA_5A_) causes a complete loss of Et efflux activity and loss of survival against TPP, and Ch, signifying the importance of the EL1‐EL7 interface in QacA activity (Fig 5C and D).

**Figure EV4:**
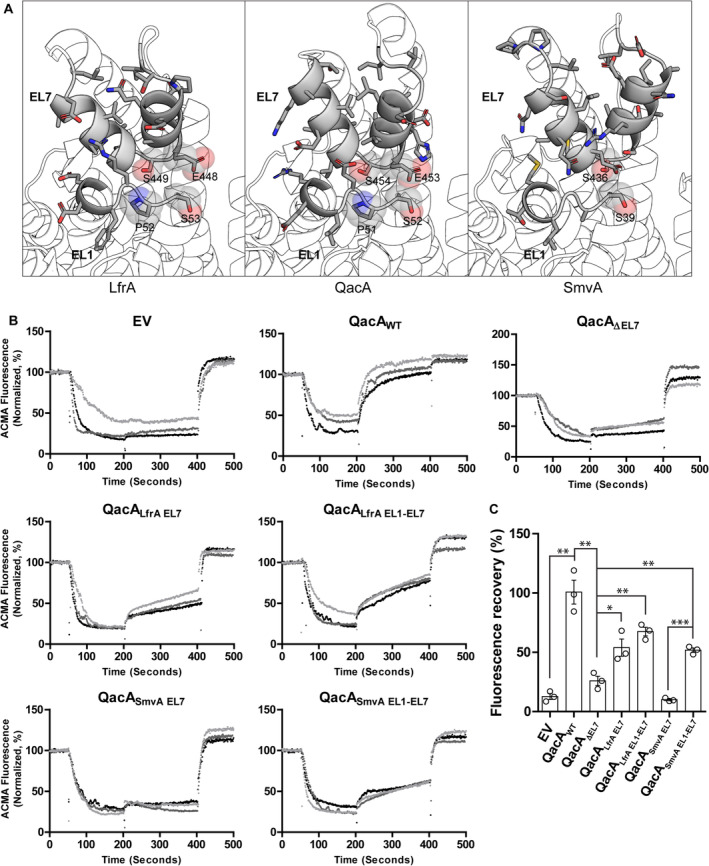
Structure similarity of EL1 and EL7 regions in QacA homologs A
Structural similarity between LfrA, SmvA, and QacA ELs 1 and 7 shown using their AlphaFold2 models. Identical residues are shown in transparent spheres.B
Everted vesicle traces (technical triplicates shown for 1 biological replicate analyzed in Fig 5J) for one of three biological replicates reported in this study. Fluorescence is normalized between data recorded between 40 and 50 s of acquisition and zero absolute fluorescence intensity.C
Histogram showing normalized ACMA fluorescence recovery during everted vesicle‐based transport assays. Data points overlaid are mean values of technical triplicates, for three biological replicates. Error bars represent SEM. Unpaired *t*‐test conducted assuming Gaussian distribution and equal variance across constructs gave the following *P*‐values: 0.001 for EV vs QacA_WT_; 0.002 for QacA_WT_ vs QacA_ΔEL7_; 0.0275 for QacA_ΔEL7_ vs QacA_LfrA EL7_; 0.0015 for QacA_ΔEL7_ vs QacA_LfrA EL1‐EL7_; 0.004 for QacA_ΔEL7_ vs QacA_SmvA EL1‐EL7_; < 0.0001 for QacA_SmvA EL7_ vs QacA_SmvA EL1‐EL7_. *P < 0.05, **P < 0.01, ***P < 0.001. Source data are available online for this figure.

Given the similarity of structural organization in models of DHA2 members like LfrA and SmvA with QacA (Fig EV4A), we explored the possibility of building EL7 chimeras among the three transporters. We initially translocated the EL7 of LfrA and SmvA into the QacA_ΔEL7_ and built chimeric constructs QacA_LfrA EL7_ and QacA_SmvA EL7_ (Fig 5E and F). While we were unable to see any recovery in the efflux activity of QacA_SmvA EL7_, we interestingly observed a recovery in the activity of QacA_LfrA EL7_ for Et efflux and survival against both monovalent TPP and divalent Ch antibacterial compounds (Fig 5G and H).

We found the EL7 of QacA to possess greater sequence identity with LfrA EL7 hairpins (33%), in comparison with SmvA (24%; Figs 5F and EV4A), hence providing a greater probability of re‐establishing the interactions between LfrA‐EL7 and QacA‐EL1. Thus, if we could improve the complementarity of the interaction at the EL1‐EL7 interface, we surmised that it could improve the gain‐of‐function seen in the EL7 chimeric constructs. To test our hypothesis, we incorporated the EL1 region of LfrA and SmvA into QacA_LfrA EL7_ and QacA_SmvA EL7_, respectively. The resultant dual mutants QacA_LfrA EL1‐EL7_ and QacA_SmvA EL1‐EL7_ were tested for their transport activity against Et and TPP through whole cell‐based efflux assay and everted vesicle‐based transport assays. We consistently found that introducing a native EL1 sequence of LfrA improved the transport activity of QacA_LfrA EL1‐EL7_. Moreover, incorporation of the native EL1 of SmvA in the seemingly inactive QacA_SmvA EL7_ background resulted in a significant enhancement in activity, with QacA_SmvA EL1‐EL7_ regaining about 50% transport efficacy relative to QacA_WT_ (Figs 5H–J and EV4B and C).

This has led us to conclude that the helical hairpin can be translocated in a modular fashion among related DHA2 members, and EL7 has a distinct role of interacting with EL1 during the transport cycle of QacA and related homologs, to form an extracellular gate during the transition from outward‐open to inward‐facing/open state.

## Discussion

**Figure 6:**
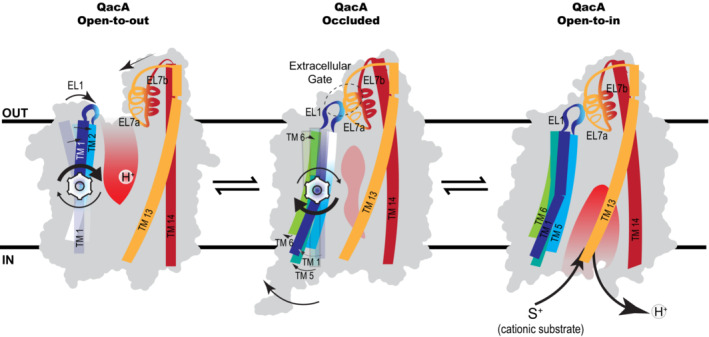
Schematic of conformational transitions in QacA The proposed mechanism progresses rightward, where at first low extracellular pH causes protonation events at the base of the vestibule. Protonation induces extracellular side of the N‐terminal helices to move toward the vestibule. EL7 starts moving toward the center of the vestibule (black arrows). Rest of the C‐terminal bundle remains relatively static (Left panel). The silhouette of QacA_oo_ is depicted in the background for positional context. This is followed by vestibule occlusion from the extracellular side. EL1 exclusively interacts with the descending part of EL7 (EL7a; dotted circle). N‐terminal bundle helices move out of the vestibule from the cytoplasmic end. Vestibule becomes accessible from the cytoplasmic side (shown by black arrow at the cytoplasmic end, middle panel). The silhouette of one of the frames observed in QacA_occ_ depicted in the background. Finally, conformational transition is shown to inward‐open state, where higher cytoplasmic pH renders coupled proton labile to competitive dissociation from substrate. In this state, EL1‐EL7 interaction does not get disrupted. Silhouette of QacA_io_ AF2 model is depicted in the background (right panel).

The study delves into the structural organization of a prototypical member of DHA2 transporters, QacA, in complex with two ICabs that do not interfere with QacA's efflux activity. The structure of QacA_D411N_ displays the symmetric organization of two helical bundles 1–6 and 9–14 with the TMs 7 and 8 forming an inserted helical pair (6 + 2 + 6 arrangement) characteristic of 14TM helix MFS transporters (Law *et al*, 2008; Kumar *et al*, 2021). The structure of QacA_D411N_ is in an outward‐open conformation with multiple acidic residues that surround the vestibule and play distinct roles in promiscuous antibacterial efflux (Majumder *et al*, 2019). Multiple acidic residues facilitate proton:drug stoichiometry > 1 to allow transport of structurally diverse divalent cationic substrates or facilitate transport of multiple substrates in a single transport cycle, both of which can make QacA highly processive (Paulsen *et al*, 1996; Tirosh *et al*, 2012). A typical efflux cycle involves a competition‐driven H^+^/substrate antiport involving the interactions of protons from the periplasmic space at a lower pH (~ 6.0–6.5) with the acidic residues in the vestibule that undergo protonation in the outward‐open conformation of the transporter (Fig 6; Schuldiner, 2014). Protonation of acidic residues coupled with the membrane potential and proton electrochemical gradients force the conformational shift of the antiporter to the cytosol‐facing state where a higher pH (~ 7.5) leads to the deprotonation of the acidic residues. This allows the cationic substrates to interact with the negatively charged deprotonated acidic residues causing the subsequent reorganization of the transporter back to the outward‐open state leading to the release and efflux of quaternary antibacterial compounds into the periplasmic space. The early stages of the transport cycle in QacA are evident in the molecular simulations of the QacA_WT_ by substituting the D411N mutant back to a protonated aspartate at 411. The D411 protonated form displays the formation of the occluded state of the transporter by closing the extracellular gate whereas the deprotonated forms do not shift from their outward‐open conformation and further cause a reversal of the QacA_occ_ state to the QacA_oo_ state thus reinforcing the residue D411 to have a crucial role in QacA's protonation‐induced conformational shifts.

**Figure EV5:**
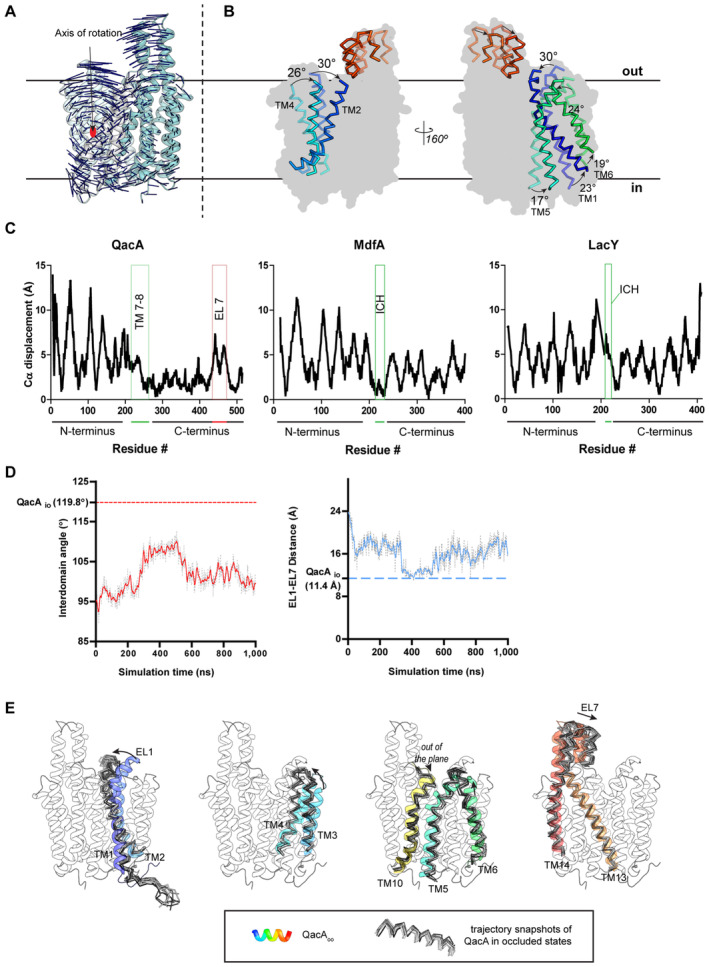
Presence of asymmetric rocker‐switch motion in QacA A
The Cα displacement vectors (blue dashes) between aligned QacA_D411N_ (QacA_oo_) structure and QacA_io_ model show axis of rotation traversing along and through the middle of the bilayer to depict asymmetry between the degree of motions between the N‐terminal and C‐terminal domains.B
Overlay of QacA_oo_ and QacA_io_ coordinates with QacA_oo_ contoured in the background for positional context. Helices shown in opaque or corresponding faded shades depict individual segments of QacA_io_ and QacA_oo_ states, respectively. A rainbow color scheme is followed from N‐ to the C‐terminal end of QacA.C
Cα displacement values are shown for structurally aligned inward‐ and outward‐open states of QacA, MdfA and LacY, with extents of N‐ and C‐terminal domains, TMs 7‐8 or ICH, and EL7 annotated. Models used for these alignments are QacA_D411N_ cryo‐EM structure and QacA AF2 model, PDBIDs 6GV1 (MdfA outward open) and 4ZP0 (MdfA inward open), and PDBIDs 5GXB (LacY outward open) and 1PV7 (LacY inward open).D
Interdomain angle (left) and EL1‐EL7 distance (right) traces extracted from one of the simulation runs having D411 sidechain neutral (protonated). One frame from 408^th^ ns was used to depict QacA_occ_ conformation. Solid lines are smoothened projections of real trace (shown as dashed gray trace). Y‐intercept in both the graphs represents the values of these CVs in QacA_io_ model.E
Snapshots (ribbons in greyscale) of 10 equally distributed frames between 300 and 500 ns of trajectory of the run described in panel (E). Small bunches of helices are shown in multiple panels for clarity. Major displacements from QacA_oo_ state are marked with arrows. A cartoon outline of QacA_D411N_ is displayed behind the helices to provide positional context. Since the comparison is made with an ensemble, displacements are not quantified in the panels. Source data are available online for this figure.

A further observation of interest using the QacA_oo_ structure, the QacA_occ_ and QacA_io_ models is that the helical bundle of TM1‐6 undergoes significantly larger rocker‐switch movements in comparison with the TMs 9–14 that remain relatively static and play a scaffolding role (Fig EV5A–C). In the N‐terminal domain, individual transmembrane helices TMs 1–2 and TMs 4–6 undergo angular motions ranging between 17° and 30° about a point lying roughly at the center of the bilayer. In the C‐terminal bundle, the EL7 hairpin along with the TM13 and TM14 marginally moves toward the vestibule to establish contact between EL7 and EL1 in the QacA_io_ conformation. The center of mass of the TM helices 1–6 remains unaltered in its position during these shifts indicating the rotation of the helical bundle around a single axis without any translation. This is in stark contrast with the other MFS members like LacY and the DHA1 member MdfA whose structures are available in both outward and cytosol‐facing states and display relatively symmetric movements of the two helical bundles (Fig EV5C; Abramson *et al*, 2003; Smirnova *et al*, 2014; Heng *et al*, 2015; Nagarathinam *et al*, 2018). Although the study does not have an experimental QacA structure facing the cytosol, the accuracy of AlphaFold2 models and the simulations done with QacA_oo_ coordinates (Fig EV5D and E) provide the insights to suggest the presence of an “asymmetric rocker‐switch” in QacA similar to the recent observation of this behavior in *Hs*PepT1 and *Hs*PepT2 where a similar asymmetry of movement for the N‐terminal domain was observed relative to the C‐terminal half (Killer *et al*, 2021).

The unique structural feature of QacA is the EL7 hairpin loop inserted between TMs13 and 14. This insertion is observed in multiple DHA2 members like LfrA and SmvA, whose structural context was previously unknown. The presence of this EL7 hairpin makes QacA and its related sequences a distinct subset among the DHA2 transporters. The region of the TM13‐EL7‐TM14 helical bundle that is solvent exposed is also the site of interactions for the two ICabs used in the study. Unlike recent studies on NorA and NorC, where the antibodies interact by inserting the CDR loops into the vestibule of the transporter and locking them in an outward‐open state (Kumar *et al*, 2021; Brawley *et al*, 2022), the ICabs interact with EL7 of QacA at distinct locations resembling a “molecular bow‐tie,” without hampering the efflux activity. Although surprising, we observe the absence of any interference of ICab A4 in the conformational transitions and ICab B7 displays a minor clash during the formation of EL1‐EL7 interface that does not seem to influence efflux activity of QacA. This is further supported by the alternate observation that substituting individual residues in the interface does not affect transport and substantial disruption of EL1‐EL7 interface is required to cause loss of efflux activity. Cases of antibodies not interfering with transport function are known, for instance in LacY, where diverse nanobodies do allow substrate interactions and affect structural dynamics (Smirnova *et al*, 2015). Also, nanobodies generated against MsbA that bind to the ATPase domains have been shown to not have an effect on their catalytic activity (Galazzo *et al*, 2022).

As observed in the study, EL7 hairpin is not a passive structural motif but plays an active role in the antibacterial efflux of QacA through the interactions it forms with EL1 during the transport cycle. Importance of the allosteric role of EL7 is evident from the loss of QacA activity observed from the disruption of the EL1‐EL7 interfacial residues and a complete deletion of the EL7 motif in QacA. Deleting the hairpin loop also severely compromises the efflux properties of QacA, despite being at a distant location in the structure away from the vestibule where protons and quaternary ammonium compounds interact. To further evaluate its mechanistic role, we built chimeric constructs on QacA_∆EL7_ background using the EL7 and EL1 motifs from related DHA2 members like LfrA and SmvA that display similar hairpin loops in their AlphaFold2 models. Remarkably, we could observe that introduction of EL7 hairpin as a module from other transporters led to the recovery of transport activity in one of the chimeras QacA_LfrA‐EL7_, which was further enhanced when the EL1 region was modified to resemble the respective transporters to re‐establish complementary interfaces between the EL7 hairpin and EL1 for the respective transporters.

The structure of QacA not only presents features among the DHA2 transporters that are consistent with other MFS transporters but also has distinct characteristics that facilitate efficient efflux activity (Drew *et al*, 2021). Primary among these is the EL7 hairpin loop that is observed to have an allosteric role through interactions with the EL1 during the transport cycle. The EL7 coincidentally occurs in DHA2 members with larger number of acidic residues in the vestibule. The motif could aid in reinforcing the extracellular gate and prevent leaks during the transport cycle. Although the QacA structure represents a conformation stabilized single mutant of QacA_D411N_, it can serve as an insightful template to study the dynamics of transport cycle and as a target for efflux pump inhibitors that can reduce efflux of widely used quaternary ammonium antibacterials in drug‐resistant strains of *Staphylococcus aureus*.

## Material and Methods

### Plasmids and strains

JD838 (∆*mdfA*∆*acrB*∆*ydhE*::*kan*) strain derived from *E. coli* K‐12 strain, LMG194, bearing a knockout of three multidrug efflux pumps was used for all the experiments in this study if not mentioned otherwise (Majumder *et al*, 2019). A codon‐optimized QacA synthetic gene (Hassan *et al*, 2009) was cloned into a pBAD‐His_8_ vector between *NdeI* and *HindIII* sites. C‐terminal GFP constructs were generated using the megaprimer‐based whole plasmid PCR method. Site‐directed mutagenesis was performed to modify QacA_WT_ to a stable functional construct QacA_D411N_ and QacA_D411N_‐GFP and cloned into pBAD‐His_8_ vector. QacA_R47A_, QacA_Q56A_, QacA_E453A_, QacA_S454A_, QacA_E460A_, QacA_5A_ (QacA with R47A/Q56A/S454A/V456A/E460A), extracellular loop (EL) deletion constructs QacA_∆EL7_ (i.e., QacA_∆I443‐T465_) and QacA_D411N/∆EL7_, and chimeric constructs QacA_LfrA EL7_ (i.e., I443‐T465 replaced in QacA with LfrA EL7 [L438‐L460]), QacA_LfrA EL1‐EL7_ (i.e., R47‐E50 replaced in QacA_LfrA EL7_ with LfrA EL1 [E48‐R51]), QacA_SmvA EL7_ (i.e., I443‐T465 replaced in QacA with SmvA EL7 [L425‐P449]), and QacA_SmvA EL1‐EL7_ (i.e., R47‐P51 replaced in QacA_SmvA EL7_ with SmvA EL1 [M34‐A38]) were cloned using the megaprimer‐based whole plasmid PCR method into pBAD‐His_8_ vector.

For QacA construct optimization process, the cytosolic rim mutants E137Q, E141Q, R142Q, A143N, D267N, L389N, and L392N were constructed using site‐directed mutagenesis with specific primers and megaprimer‐based whole plasmid PCR was used to clone them in QacA_D411N_ pBAD‐His_8_ backbone. The protein expression profiles were checked using JD838 strain of *E. coli*. The ICab library was cloned into the pETCON vector between *NdeI* and *XhoI* restriction sites. The ICab library was expressed in *Saccharomyces cerevisiae* EBY100 strain, used for FACS and flow cytometry analysis. The ICabs were subcloned in pET22b vector without pelB signal sequence and overexpressed in SHuffle T7 cells of *E. coli* (NEB, catalog no. C3026J).

### QacA protein purification

QacA and its mutants used in this study were purified following the protocol as described in our earlier study (Majumder *et al*, 2019). The transformed JD838 cells were grown in the presence of 100 μg/ml ampicillin at 37°C till it reaches 0.6 OD_600_. The cells were induced with 0.05% (w/v) of L‐arabinose and grown overnight at 18°C. The harvested cells were resuspended in the lysis buffer (30 mM phosphate buffer pH 7 and 120 mM NaCl, 5% v/v glycerol), and cells were lysed using high‐pressure homogenization at 4°C. All the steps of the purification were performed at 4°C. The unlysed cells were removed by centrifugation at ~ 20,000 *g* for 15 min, and the membrane was purified using ultracentrifugation at 100,000 *g* for 1 h. The purified membrane was homogenized in lysis buffer. The protein was extracted using 20 mM UDM detergent and kept for solubilization for 1 h. The remaining cell debris and insoluble membrane were removed using ultracentrifugation at 100,000 *g* for 1 h. The supernatant was kept for binding with pre‐equilibrated Ni‐NTA beads for 1 h. The beads were washed with wash buffer (30 mM imidazole, 30 mM phosphate buffer pH 7 and 120 mM NaCl, 5% v/v glycerol, 1 mM UDM) and eluted in the elution buffer (300 mM imidazole, 30 mM phosphate buffer pH 7 and 120 mM NaCl, 5% v/v glycerol, 1 mM UDM). The freshly eluted protein was quickly concentrated using a 50 kDa cutoff concentrator and purified further by size exclusion chromatography using Superdex™ 200 increase 10/300 GL column.

### ICab library generation

ICab library was generated as described (Kumar *et al*, 2020). A male camel of age 4–5 years was immunized in primary and seven boosters regimen with purified QacA_D411N/E137Q_ protein in detergent micelles and reconstituted in proteoliposomes. The blood was collected after 7 days of final immunization, and peripheral blood mononuclear cells (PBMCs) were isolated. The total RNA was purified from PBMCs and reverse transcribed into cDNA using the RevertAid First Strand cDNA Synthesis Kit (Thermo Scientific, K1621). The VHH open reading frame was amplified from cDNA, and the PCR product along with the linearized pETCON plasmid was electroporated in EBY100 strain of *S. cerevisiae* as explained (Benatuil *et al*, 2010). Positive transformants were selected and grown in SDCAA media, and induced in SGCAA media, followed by FACS sample preparation as described (Ahmed *et al*, 2021). The cells were labeled with anti‐HA antibody conjugated to Alexa Fluor™ 647 (Invitrogen, 26183‐A647), and QacA‐GFP was used to estimate the levels of expression and binding of the ICabs on the yeast cell surface. The cells that were positive for both the ICab expression and QacA‐GFP binding were enriched for three rounds using FACS. A concentration of 1,000 nM, 300 nM and 100 nM QacA‐GFP was used in first, second, and third round of sorting, respectively, and a total of 20 million cells were used for sorting in each round on BD FACSAria™ III Cell Sorter. Expression and binding of unique individual ICabs clones were analyzed on BD Accuri C6 flow cytometer as described (Ahmed *et al*, 2021). To estimate the expression of individual ICabs, anti‐c‐Myc antibody conjugated to Alexafluor™ 647 (catalog no. MA1‐980‐A647) was used. Individual ICabs' affinity to QacA_D411N_ on the yeast cell surface was estimated using flow cytometry as described in earlier studies (Kumar *et al*, 2020; Chattopadhyay *et al*, 2022). Briefly, the yeast cells expressing ICabs were incubated with different concentrations of QacA_D411N_‐GFP, and the amount of binding was plotted. The data were fitted using GraphPad Prism v5 using one site‐specific binding to estimate the apparent *K*_d_.

### ICab expression and purification

ICabs (A2, A4, and B7) were cloned into pET22b vector and expressed in the SHuffle T7 *E. coli* cells. The cells were induced with 1 mM IPTG and grown at 30°C. The harvested cells were resuspended in the lysis buffer (30 mM HEPES pH 7.4, 120 mM NaCl) and sonicated. The cell debris and insoluble aggregates were removed using centrifugation at ~ 20,000 *g* for 1 h, and the supernatant was kept for binding with pre‐equilibrated Ni‐NTA resin. The column was washed with 50 column volumes of wash buffer (30 mM HEPES pH 7.4, 120 mM NaCl and 30 mM imidazole), and the protein was eluted with elution buffer (30 mM HEPES pH 7.4, 120 mM NaCl and 300 mM imidazole). The purified protein was further enriched by size exclusion chromatography using Superdex™ 75 10/300 GL column.

### Epitope competition assay with ICabs displayed on the yeast cell surface

To check the epitope competition between ICabs, purified QacA_D411N_‐GFP (100 nM) was incubated with purified ICab A2/A4/B7 (1,000 nM) for 30 min at 4°C. This preformed complex of QacA_D411N_‐GFP‐A2/A4/B7 was incubated with the yeast cells expressing ICabs on their cell surface. Flow cytometric analysis was performed to estimate the amount of binding of the preformed complex on the yeast cell surface. No binding was observed if the yeast cell surface‐displayed ICab and the purified ICab shared the same epitope region suggesting competitive interactions.

### Spheroplast preparations for imaging

pBAD‐His_8_ QacA_D411N_‐GFP transformed *E. coli* JD838 cells were grown overnight at 37°C. The secondary culture (5 ml) was induced with 0.05% (w/v) L‐arabinose, and the cells were harvested as the OD_600_ reached 0.5–1.0. The harvested cells were washed with 1× PBS, 0.1% (w/v) BSA solution. The cell pellet was resuspended in 500 μl of 800 mM sucrose solution. To form the spheroplasts, the following solutions were added in the described order: (i) 30 μl of 1 M Tris–HCl pH 8, (ii) 24 μl of 0.5 mg/ml lysozyme, (iii) 6 μl of 5 mg/ml DNase, (iv) 6 μl of 125 mM EDTA‐NaOH pH 8, and (v) 5 μl of 1 mM of spermidine. The mixture was incubated at room temperature for 20 min. The spheroplasts were harvested by low‐speed centrifugation at 500 *g* for 3 min. To stabilize the spheroplasts, 4 μl of 1 mM Tris–HCl pH 8, 400 μl of 800 mM sucrose, 8 μl of 1 mM MgCl_2,_ and 4 μl of 1 mM of spermidine were added to the spheroplasts and the volume was brought upto 400 μl with sterile water.

### Confocal sample preparation

ICabs A4 and B7 were labeled with NHS‐Rhodamine (ThermoScientific, 46406) as described by the manufacturer. For conjugation, a molar ratio at 10:1 for NHS‐rhodamine: protein was used in the conjugation buffer (20 mM HEPES pH 7, 150 mM NaCl). The reaction was stopped by the addition of molar excess of Tris buffer pH 8. The unreacted dye was removed using PD‐25 desalting columns. The NHS‐Rhodamine labeled ICabs were added to the freshly prepared spheroplasts at a final concentration of 0.03 mg/ml. The spheroplasts were incubated at room temperature for 30 min and washed thrice with 1× PBS, 0.1% BSA solution to remove excess ICabs. Furthermore, 10 μg/ml final concentration of DAPI was added to spheroplasts. The spheroplasts were washed once again to remove extra DAPI with 1× PBS, 0.1% BSA solution. The spheroplasts were then fixed with a drop of ProLong™ Gold Antifade Mountant (Thermo Scientific, P10144). The slides were observed under confocal microscope (Olympus FV3000 or Zeiss LSM 880 with Airyscan) for DAPI (λ_Ex_/λ_Em_ = 358 nm/461 nm), NHS‐rhodamine (λ_Ex_/λ_Em_ = 552 nm/575 nm) and GFP (λ_Ex_/λ_Em_ = 488 nm/512 nm).

### Binding study using FSEC

Purified QacA_D411N_ was mixed with ICab (A4, B7) at a 1:1.2 ratio and incubated at 4°C for 1 h. The shift in FSEC profile was observed by comparing QacA_D411N_‐ICab complex and QacA_D411N_. The intrinsic tryptophan fluorescence was measured λ_Ex_ at 294 nm and λ_Em_ at 334 nm.

FSEC shift measurements of GFP‐tagged QacA constructs in detergent‐solubilized membranes were done by mixing purified ICabs A4 or B7 followed by incubation at 4°C for 1 h. GFP‐fluorescence (λ_Ex_ at 488 nm and λ_Em_ at 510 nm) was measured to compare the elution volumes of free QacA and QacA‐ICabs complex.

### Cryo‐EM sample preparation and data collection

SEC‐purified QacA_D411N_ in 30 mM Hepes pH7.0, 120 mM NaCl, 2% glycerol and 1 mM UDM buffer was concentrated to 4 mg/ml using a 100 kDa cutoff centricon. QacA_D411N_ and ICabs A4 and B7 were mixed in a 1:1.2:1.2 (QacA:A4:B7) molar ratio. To ensure QacA_D411N_‐A4‐B7 complex formation, a small aliquot from concentrated peak fraction of QacA_D411N_‐A4‐B7 complex was analyzed using FSEC. The concentrated protein sample was ultracentrifuged at 100,000 *g* at 4°C for 1 h just before the grid freezing. Quantifoil gold (R 0.6/1) holey carbon grids were used to freeze QacA_D411N_‐A4‐B7 protein sample using a Vitrobot Mark IV (ThermoFisher). The grid freezing was carried out in 100% humidity, with 5 s blot time, 10 s wait time, and at 16°C. Data for QacA_D411N_‐A4 complex were collected on a Titan Krios equipped with a K2 detector (Appendix Fig S3). Grid screening for QacA_D411N_‐A4‐B7 complex was done using an in‐house Talos Arctica 200 keV cryo‐electron microscope, and the grids with good particle distribution in thin ice were sent to the eBIC‐Diamond Light Source, UK, where a complete dataset was collected on a Titan Krios 300 keV electron microscope equipped with Gatan BioQuantum K3 imaging filter and detector. A total of 7,770 movies were collected in super‐resolution mode, 2× binning with a pixel size of 0.831 Å/pixel. The total electron dose was 51 e^−^/ Å^2^ with an exposure time of 2 s and an energy filter slit of 20 eV. The electron dose was fractionated over 50 frames.

### Cryo‐EM data processing and refinement

The data were processed using cryoSPARC v3.0 (Punjani *et al*, 2017). The imported 7,770 movies were motion‐corrected using patch motion correction, and CTF estimation was done using patch CTF estimation (Appendix Fig S3). The poor‐quality micrographs were removed using a manually curate exposure tool present in cryoSPARC (Punjani *et al*, 2017) with a cutoff of 1.05 ice thickness and CTF correlation > 5.0 Å, and a total of 6,310 micrographs were selected for further processing. 158,327 particles were picked automatically using the blob picker tool from 400 micrographs, and using inspect particle pick, 136,503 particles were chosen. The 2D classification was performed with extracted 107,759 particles. Twelve 2D classes were selected, which consisted of 26,890 particles. These particles were used as templates for the template picker tool, and a total of 5,723,316 particles were picked from 6,310 micrographs. Particles were further inspected using inspect particle pick tool, and 2,276,055 particles were selected. 1,666,340 particles were extracted from the selected particles, and 2D classification was performed. From the first round of 2D classification, 502,364 particles were selected and further two rounds of 2D classifications were performed to remove noise. Finally, from the last 2D classification 218,040 particles were selected from 97 2D classes. A single *ab initio* model was built with 218,040 particles, and homogeneous refinement was performed with a maximum align resolution of 3 Å and keeping other values as default. The GSFSC value after homogeneous refinement was 4.85 Å. Furthermore, nonuniform refinement was performed taking previously refined volume as input. In the case of nonuniform refinement (Punjani *et al*, 2020), the number of extra final passes was changed to 5, maximum alignment resolution was changed to 2 Å, and GSFSC fit resolution was kept at 5 Å. Also, the initial batch size was changed to 2000 with a batch epsilon value of 0.01 and the dynamic mask start resolution was kept at 7 Å. The value of GSFSC resolution after nonuniform refinement became 3.84 Å following the standard FSC cutoff value of 0.143. Furthermore, the map resolution was significantly improved by density modification using the Phenix Resolve density modification tool (Terwilliger *et al*, 2020), and the final resolution after density modification was 3.6 Å. QacA_D411N_ and ICabs A4 and B7 AlphaFold2 models were initially fitted into the map using Chimera (Pettersen *et al*, 2004), and the model was built using Coot (Emsley & Cowtan, 2004). The model was refined using Phenix real‐space refinement program to remove the outliers and improve the model refinement statistics (Afonine *et al*, 2018). After iterative real‐space refinement, model matches to the density‐modified map with a CC of 0.77 (Appendix Table S1). The local resolution of the map was calculated using local refinement in Phenix (Terwilliger *et al*, 2020).

### Drug resistance assay

Drug resistance assay was performed in the presence of Dq, Ch, Et, and TPP with JD838 cells expressing QacA_WT_ and its mutants described above, along with cells harboring empty pBAD vector. The experiment was performed on 2% w/v Luria‐Bertani (LB) media and 1.5–1.8% (w/v) agar. Cells were diluted to an OD_600_ of 1.0, and a series of 10‐fold dilutions were prepared, and 2 μl from each dilution was spotted on LB‐agar plates. The plates contained 0.05% (w/v) L‐arabinose and 100 μg/ml Ampicillin, with or without the addition of substrates Dq (15 μM), TPP (10–15 μM), Et (25 μM) and Ch (16 μM). The survival of the cells was checked after 14 h of incubation at 37°C to check the resistance against Dq, TPP, Et and Ch.

### Whole cell and spheroplast preparation for ethidium efflux assay

Whole cell‐based ethidium efflux assay was done as previously described (Majumder *et al*, 2019). JD838 cells harboring pBAD‐*qacA*_*WT*_ and its mutants, or empty vector were inoculated in 2% LB broth (w/v), which were used as 1% inoculum for 10 ml of secondary cultures. The cells from the secondary culture were induced with 0.05% (w/v) L‐arabinose and grown till the OD_600_ reached 0.6. The cells were washed with 20 mM HEPES pH 7.0 twice and incubated with 20 μM EtBr and 0.5 μM carbonyl cyanide m‐chlorophenyl hydrazone (CCCP) for 1 h in dark at 37°C. The cells were washed thrice and finally resuspended in 2 ml of 20 mM HEPES pH 7.0 for the efflux assay.

To prepare for spheroplast‐based efflux assays, cultures were grown using JD838 cells harboring pBAD‐*qacA*_*WT*_. Secondary culture in 100 ml 2% LB broth with 100 μg/ml ampicillin was split into two equal volumes. 0.1% w/v L‐arabinose was added to one of the 50 ml cultures to induce QacA overexpression, while the other was left uninduced. When the cultures' OD_600_ reached 0.6 A.U., equal number of cells were harvested by centrifugation at 3,000 *g* for 15 min and washed twice with 1× phosphate buffer saline (pH 7.4). The cells were incubated with 20 μM EtBr and 0.5 μM CCCP for 30 min and then washed thrice with PBS. The cells were incubated in 90 ml of spheroplast solution (700 mM sucrose, 10 mM EDTA, 20 mM Tris pH 7.2 and 0.2 mg/ml lysozyme) for 20 min at 4°C with constant and slow tumbling. The spheroplasts were stabilized by adding 10 ml of ice‐cold stabilizing solution (700 mM sucrose, 10 mM MgCl_2_, and 20 mM Tris pH 7.2) to the suspension. The spheroplasts were harvested and washed by centrifugation at 900–1,000 *g* for 15 min each and finally resuspended PBS and 1 mM spermine. When incubating with ICabs, it was assumed on the basis of prior protein expression trials that the cultures at an O.D._λ = 600nm_ of 2.0 A.U. harbored 4–8 μg/ml of QacA_WT_, and after incubating these spheroplasts with ICabs in concentrations ranging between 0.1 and 1.0 mg/ml in different trials for optimization, the QacA:ICab molar ratio always ranged between 10 and 100.

As protein‐specific inhibition control, 5 μM Reserpine was added to whole cells as well as spheroplasts 1–2 min prior to ethidium efflux measurement.

### Ethidium efflux assay measurement

The assays were done in a 96‐well corning flat bottom black plate with 90 μl of cells or spheroplasts. ICabs were purified in 10 mM Tris pH 7.2 buffer and 150 mM NaCl for the spheroplasts‐based assay, and the same buffer was used as buffer control in the assay. A final concentration of 0.2 mg/ml of each ICab was used to incubate with spheroplasts. The cells were energized with 5 mM glucose, whereas the spheroplasts were energized using 2% (w/v) of glucose and fluorescence measurements at λ_Ex/Em_ = 530 nm/610 nm were done on Thermo Varioskan Flash with 100 ms long measurements after every 30 s.

### Everted vesicle preparation

Eight hundred milliliter of secondary culture was grown for 8–10 h at 20°C in terrific broth after induction of protein overexpression with 0.05% L‐Arabinose. The cells were harvested at 5,000 *g* for 30 min followed by a washing step with 50 mM potassium phosphate buffer, pH 7.0. The cell pellet thus obtained was dissolved in 60 ml of 50mM potassium phosphate buffer and incubated with 0.2 mg/ml chicken egg white lysozyme for 30 min with gentle shaking at 60 rpm at 30°C. The bacterial cells were lysed in 5–6 cycles using GEA Niro Soavi PandaPlus homogenizer at 400–500 bar pressure. The lysate was then incubated with 1 mM MgSO_4_ solution for 30 min with gentle shaking at 30°C. The unbroken cells were removed using centrifugation at 6,000 *g* for 30 min at 4°C. The supernatant was ultracentrifuged at 111,000 *g* for 90 min to extract the membrane vesicles. The vesicles were resuspended in 1 ml buffer containing PBS and 10% glycerol. Aliquots of 50 μl were made, snap frozen, and stored at −80°C for later use.

Fifty microliter of vesicle aliquot was quickly thawed and diluted in 2 ml solution containing PBS and 10 mM MgSO_4_. To this, 0.5 μM of Valinomycin and 4 μM of ACMA dye was added and mixed gently. After incubating for 5 min, kinetic fluorescence measurements were taken using Fluoromax‐3 (Horiba) fluorescence spectrophotometer at λ_Ex_ = 409 nm, λ_Em_ = 474 nm with continuous stirring. One hundred micromolar ATP, 1 mM TPP (substrate), and 2 μM nigericin were added after 50, 200, and 400 s from the start of the kinetic measurement. Five hundred seconds of kinetic measurements were recorded in every assay. For each construct, vesicles were made thrice independently, and from every batch, three aliquots each were assayed as technical replicates.

Fluorescence recovery was quantified as percentage ratio ((C − B)/(A − B)) × 100, where A, B, and C are the average fluorescence values between 40 and 50, 190 and 200, and 395 and 400 s, respectively (corresponding to time before adding ATP, substrate, and Nigericin, respectively). For each biological replicate, average fluorescence recovery from the three technical replicates was plotted on histogram. For everted vesicles‐based transport assays against sodium gluconate, 100 μM ATP, sodium gluconate at various concentrations, 0.5 mM TPP, and 2 μM nigericin were added 50, 200, 300, and 400 s after the start of the kinetic measurement. Five hundred seconds of kinetic measurements were recorded in every assay. From one batch, three aliquots each were assayed as technical replicates.

### Western blots to analyze the expression of QacA constructs in whole cells/membrane vesicles

For whole cell‐based efflux assay samples, the O.D._600nm_ was normalized as it was during the assay. The cells were harvested, washed with 20 mM HEPES buffer pH 7.0, and again dissolved in the same buffer with SDS–PAGE loading dye. The sample was vortexed and kept at R.T. for half an hour followed by a quick spin at ~ 20,000 *g* for 10 min. The supernatant was loaded on SDS–PAGE.

For everted vesicle‐based assay samples, one 50 μl aliquot of every sample from each batch was thawed and diluted in a final concentration of 50 mM of potassium phosphate buffer (pH 7.0). To them, a final concentration of 20 mM of UDM was added and the sample was kept on nutation for 1 h at 4°C. The samples were then ultracentrifuged at 50,000 *g* for 30 min and the supernatant was loaded on SDS–PAGE.

To probe for expression of QacA constructs, the gel was transferred to a PVDF membrane using wet western blot transfer for 120 min at 90 volts. The blot was blocked using 5% skimmed milk dissolved in 0.1% Tween‐20 dissolved in Tris buffer saline, pH 7.4 (TBST) overnight at 4°C. The blot was washed with TBST twice and then incubated with 1:1,000 dilution of anti‐His_6_‐tag mouse antibody (Invitrogen, MA1‐21315) at 4°C overnight. After three washes with TBST, the blot was incubated with 1:10,000 dilution of goat HRP conjugated anti‐mouse‐Fc antibody (Invitrogen, 62‐6520) for 50 min at RT. The blot was washed 4–5 times with TBST and was probed through chemiluminescence of HRP substrate (Immobilon, WBLUF0100).

### APBS electrostatic maps preparation and representation

To visualize the general electrostatic environment, coordinates of QacA (this study), MdfA (PDB ID: 6GV1) and LmrP (PDB ID: 6T1Z) were parsed through the Adaptive Poisson–Boltzmann Solver (APBS) plugin in PyMOL v2.5.0, which uses AMBER ff99 forcefield. A gradient from red to blue through white colors was used to represent a ramp of −10 to + 10 *k*_B_Te_c_^−1^ in the generated electrostatic maps. All acidic and basic residues were considered solvent exposed and hence charged, and their relative solvent accessibility was not derived from the structure for this purpose. The APBS uses known dielectric constants and local electric potentials for bulk water, organic molecules and many ions for implicit solvation and calculating electric fields around the macromolecule (Jurrus *et al*, 2018).

### Molecular dynamics simulations

A simulation box was generated for the coordinates of QacA_D411N_ where residue 411 was reverted to aspartic acid using CHARMM‐GUI (Jo *et al*, 2008; Wu *et al*, 2014; Lee *et al*, 2016). CHARMM36m forcefield was used for the simulation run on Gromacs‐2020 MD engine. All the acidic residues that were solvent exposed were kept deprotonated except D411, which was modeled either with a neutral side chain, deprotonated side chain, or as asparagine. The setup contained > 124,000 atoms made of QacA, 224 and 70 molecules of POPE and POPG, respectively, in the bilayer, charge neutralized 150 mM NaCl and water molecules. The system was energy minimized using steepest gradient descent and equilibrated stepwise with decreasing positional restraints. Either Berendsen and Nose‐Hoover, or V‐rescale thermostat were used for temperature coupling, and Berendsen and Parrinello‐Rahman barostats with semi‐isotropic p‐coupletype were used for pressure coupling, during equilibration and production MD runs, respectively. After box preparation, energy minimization was done independently for every setup to generate three to 10 different random seeds as replicates. Multiple simulation runs in the D411 protonated state setups yielded a stable occluded state, which was further used to generate a simulation box with one of the frames in that state (408^th^ ns from run2) as the starting model and three different random seeds. This setup had D411 with a deprotonated side chain. Another setup was created with D411 deprotonated model where we used 150 mM KCl instead of NaCl.

For simulation analyses, all trajectories were written out in pdb format and analyzed using in‐house codes. For tracking ions in the simulation runs and their 2D projections, frames at ns timesteps were taken into account, with ~ 1,000 frames as input per analysis. For tracking water molecules for correlation plots, frames at a timestep of 100 ps were considered, which were grouped in batches of 100 frames chronologically ordered. Vestibule was defined as the space occupied by water molecules within the limits that were defined as follows: the Z‐axis component of water molecules should reside within the range delimited by the coordinates of phosphate headgroups of lipid bilayer; and in the XY planes, water molecules should not be farther than 15 Å from a line along Z‐axis that ran through the center of mass of all Cα atoms. The limits were refreshed for every frame considered.

The following parameters were defined for correlation plots to generate input data points per batch: water retention, “Interdomain angle,” and “EL1‐EL7 distance.” Water retention is the average number of frames a unique water molecule is seen in the vestibule of the transporter. The average was taken against all 100 frames of a batch. Interdomain angle is defined as the angle between three points, com1, com2, and com3, where com1 is the center of mass of Cα atoms of L21, A78, L87, and T128 (representing cytoplasmic end of the N‐terminal bundle), com2 is the center of mass of Cα atoms of L42, L57, V110, and F177 (representing the extracellular end of the N‐terminal bundle), and com3 is the center of mass of Cα atoms of Q302, G313, S426, and F478 (representing the extracellular end of the C‐terminal bundle). EL1‐EL7 distance is defined as the distance between the center of masses of Cα atoms of EL1 and EL7 residues. EL1 and EL7 for this purpose were defined using the residue ranges E48‐T54 (both inclusive, com4) and I443‐T465 (both inclusive, com5), respectively. For interdomain angle and EL1‐EL7 distances correlation (used for validation purposes only, not described in the figures) with water retention readouts, only the last frame of every batch was taken into consideration, as a 10 ns time scale (encompassing 100 frames or 1 batch) generally does not record significant within‐batch variation of these parameters. Since all three these parameters were expected to have a Gaussian distribution, correlation analyses were done using Pearson statistics.

More than 25 μs of MD trajectory was used for analysis.

### Statistical analysis

For all biochemical assays involving kinetics measurements, technical replicates of one of the biological replicates were represented to avoid overnormalization, while data for all other biological replicates have been provided along with the source data. Standard error of mean (S.E.M.) was used to calculate error for all experiments. Each data point shown in Expanded view Fig EV4C is the mean obtained from technical triplicates from one biological replicate, and significance test was conducted for values from three such biological replicates (unpaired *t*‐test was conducted assuming Gaussian distribution and equal variance across constructs). Kinetic measurement graphs were normalized to respective first data points in case of everted vesicle assays and to the highest value recorded for the mean of the observations in ethidium efflux assays. A nonlinear fit was performed for the plots shown in Fig 1E and Expanded view Fig EV1A. Polynomial smoothening was performed to trace solid lines in Expanded view Fig EV5D.

### Evolutionary analysis by maximum likelihood method

The evolutionary history was inferred by using the Maximum Likelihood method and JTT matrix‐based model (Jones *et al*, 1992). The bootstrap consensus tree inferred from 500 replicates is taken to represent the evolutionary history of DHA2 members annotated in the TCDB. Branches corresponding to partitions reproduced in < 50% bootstrap replicates were collapsed. Initial trees for the heuristic search were created using Neighbor‐Join and BioNJ algorithms to a matrix of pairwise distances estimated using the JTT model and then selecting the topology with superior log likelihood value. This analysis involved 74 amino acid sequences. There were a total of 916 positions in the final dataset. Evolutionary analyses were conducted in MEGA X (Kumar *et al*, 2018).

## Author contributions

**Puja Majumder:** Conceptualization; resources; software; formal analysis; validation; investigation; visualization; methodology; writing – review and editing. **Shahbaz Ahmed:** Resources; formal analysis; investigation; visualization; methodology. **Pragya Ahuja:** Resources; formal analysis; investigation; visualization; methodology; writing – review and editing. **Arunabh Athreya:** Resources; data curation; software; formal analysis; validation; investigation; visualization; methodology; writing – review and editing. **Rakesh Ranjan:** Methodology. **Aravind Penmatsa:** Conceptualization; resources; data curation; formal analysis; supervision; funding acquisition; validation; visualization; writing – original draft; project administration.

## Disclosure and competing interests statement

The authors declare that they have no conflict of interest.

## Supporting information



Appendix S1

Expanded View Figures PDF

Dataset EV1

Source Data for Expanded View

PDF+

Source Data for Figure 1

Source Data for Figure 4

Source Data for Figure 5

## Data Availability

Cryo‐EM density map has been deposited in the Electron Microscopy Data Bank under accession number EMD‐33612. Model coordinates have been deposited in the Protein Data Bank under accession number 7Y58. All other data needed to evaluate the conclusions in the paper are present in the paper and/or the supplementary materials. Aliquots of purified ICabs would be available to research groups upon reasonable request. Source data are provided with the manuscript.
