# Supplementary material for: Cryo‐EM structure of antibacterial efflux transporter QacA from Staphylococcus aureus reveals a novel extracellular loop with allosteric role
Source: EMBO J. 2023 Jul 17;42(16):e113418. doi: 10.15252/embj.2023113418 (PMC10425836; doi:10.15252/embj.2023113418)
Supplement: Supplementary file 1 — Appendix S1 [file EMBJ-42-e113418-s003.pdf]

Appendix Figures

Appendix Figure S1 - Screening of QacA mutants for immunization.

Appendix Figure S2 - Five unique ICabs isolated against QacAD411N.

Appendix Figure S3 - Workflow for structural determination of QacA-ICabs complex through cryoEM.

Appendix Figure S4 - Model fit for QacA-ICab complex and individual TM helices.

Appendix Figure S5 - Ion specificity in QacA’s vestibule.

Appendix Figure S6 - Sequence and structural alignments of QacA with DHA1/2 transporters.

Appendix Figure S7 - Viability assay for spheroplasts based Ethidium efflux assay.

Appendix Figure S8 - Collective variables analyzed for various simulation runs on QacA.

Appendix Figure S9 - Expression analysis of different constructs designed for functional studies on QacA using Western Blotting.

Appendix Figure S10 - Ethidium efflux assay of single alanine mutants at EL1-EL7 interface.

Appendix Table S1 - CryoEM data collection and processing statistics

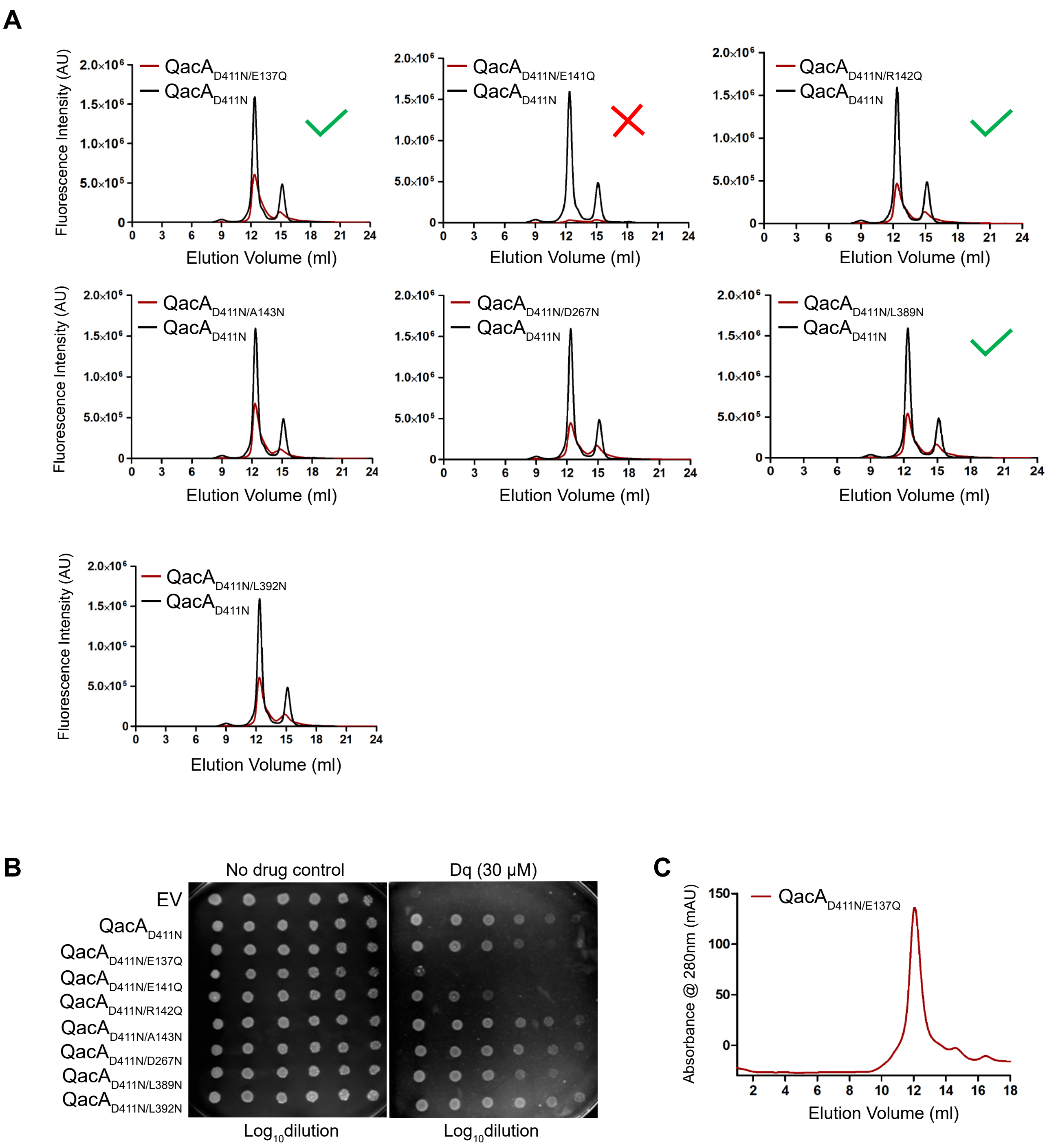

Appendix Figure S1 - Screening of QacA mutants for immunization

A FSEC profiles of all the screened cytosolic rim residue mutants.

B Survival assay on JD838 cells to screen for a stable construct of QacA. Constructs with monodisperse FSEC profile but with reduction in function (i.e., E137Q, E142Q and L389N in the background of D411N) were shortlisted, as such constructs could be more stable in one conformation and out of these, QacA$_{D411N/E137Q}$ was purified and used for immunization. EV stands for empty vector, QacA mutants are labelled.

C SEC profile of QacA$_{D411N/E137Q}$ mutant.

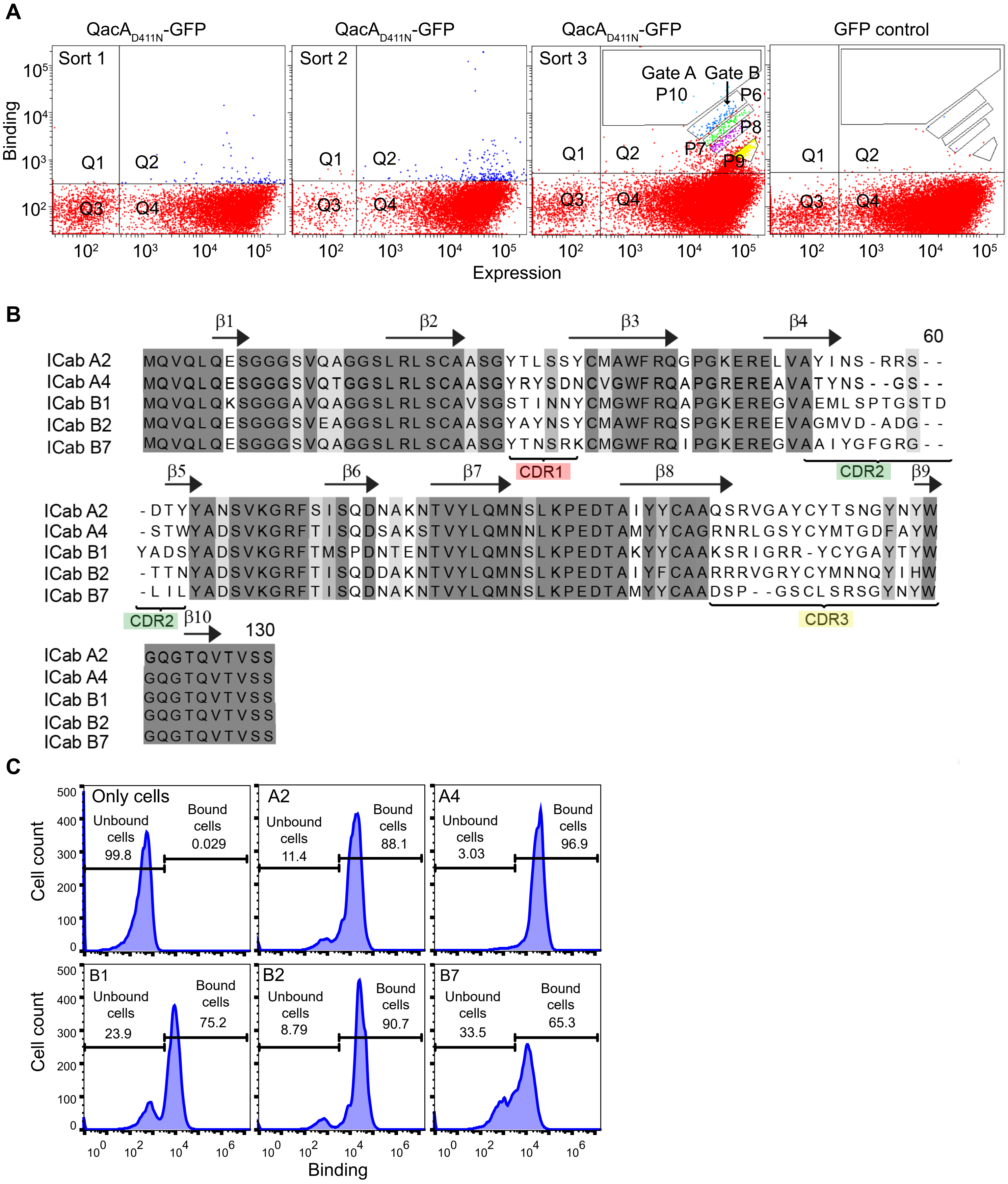

Appendix Figure S2 - Five unique ICabs isolated against QacA$_{D411N}$.

A, FACS enrichment of ICab binders against QacA through sequential sorting of Q2 from Sort 1 to Sort 3. ICabs were isolated from Gates A (P10) and B (P6).

B, Multiple sequence alignment of ICabs A2 and A4 (from P10), and B1, B2 and B7 (from P6) with higher sequence conservations highlighted with saturated shades of grey.

C, Population shifts of yeast cells expressing ICabs A2, A4, B1, B2 or B7 on their surface in the presence of micellar QacA$_{D411N}$-GFP. Bound and unbound fractions are represented in percentages.

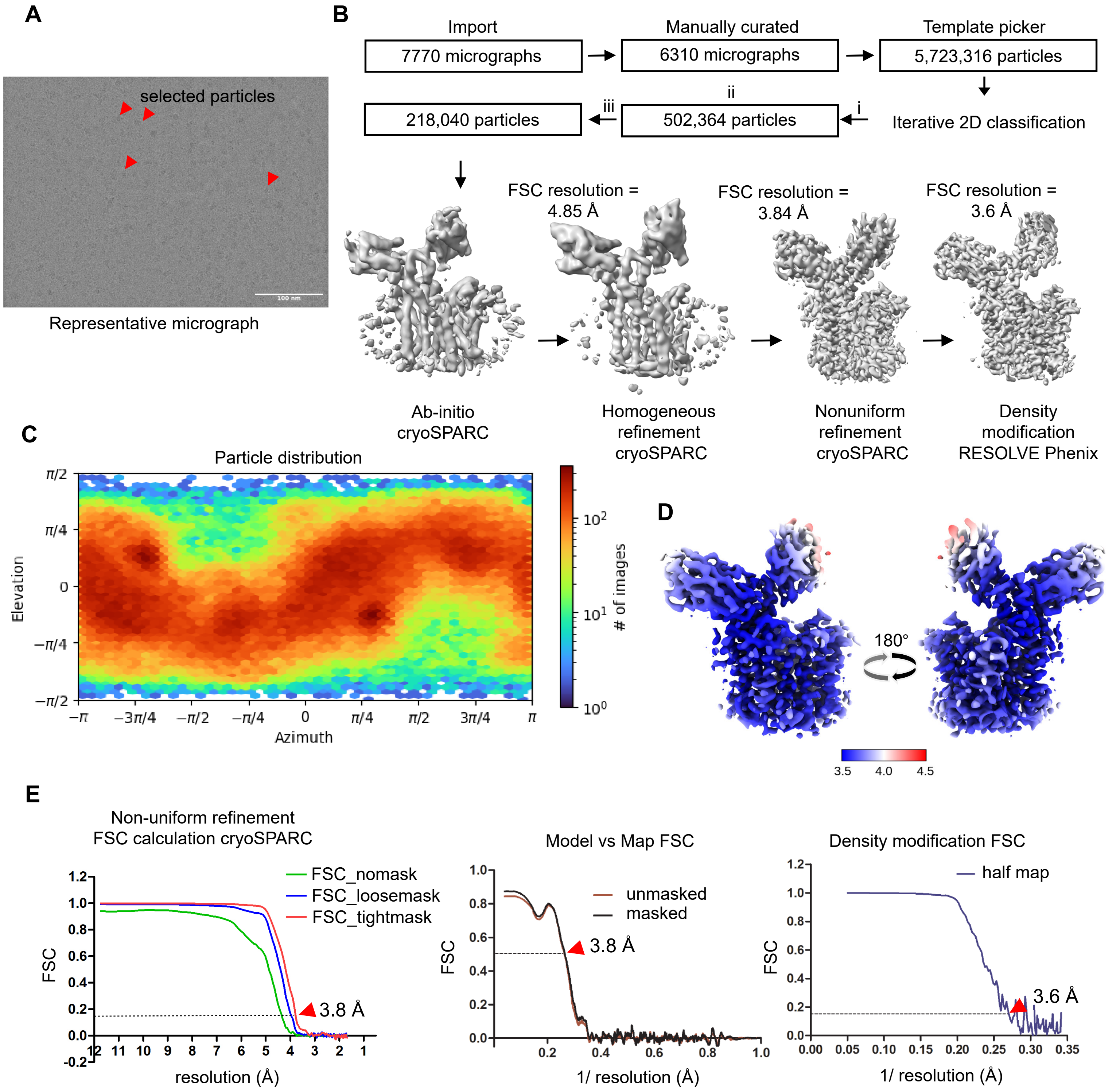

Appendix Figure S3 - Workflow for structure determination of QacA-ICabs complex through cryoEM.
A, Representative micrograph from the 7770 movies collected. Scale bar represents 100nm.
B, Summarised workflow of the data processing in CryoSPARC. Each step written is followed below by a value for micrographs/particles used in that step (boxed). For iterative 2D class averaging, 5,723,316 particles were picked in iteration (i), followed by 502,364 and 218,040 particles in iteration (ii) and (iii) respectively.
C, 2D heat map of particle orientation distribution in the processed data.
D, Local resolution maps of QacA-ICabs complex in the final structure. Scale bar units are in Angstroms.
E, Fourier shell correlation (FSC) plots of the final dataset with resolutions pointed at FSCs of 0.143 and 0.5 for non-uniform refinement and density modification, and model versus map FSC comparison respectively.

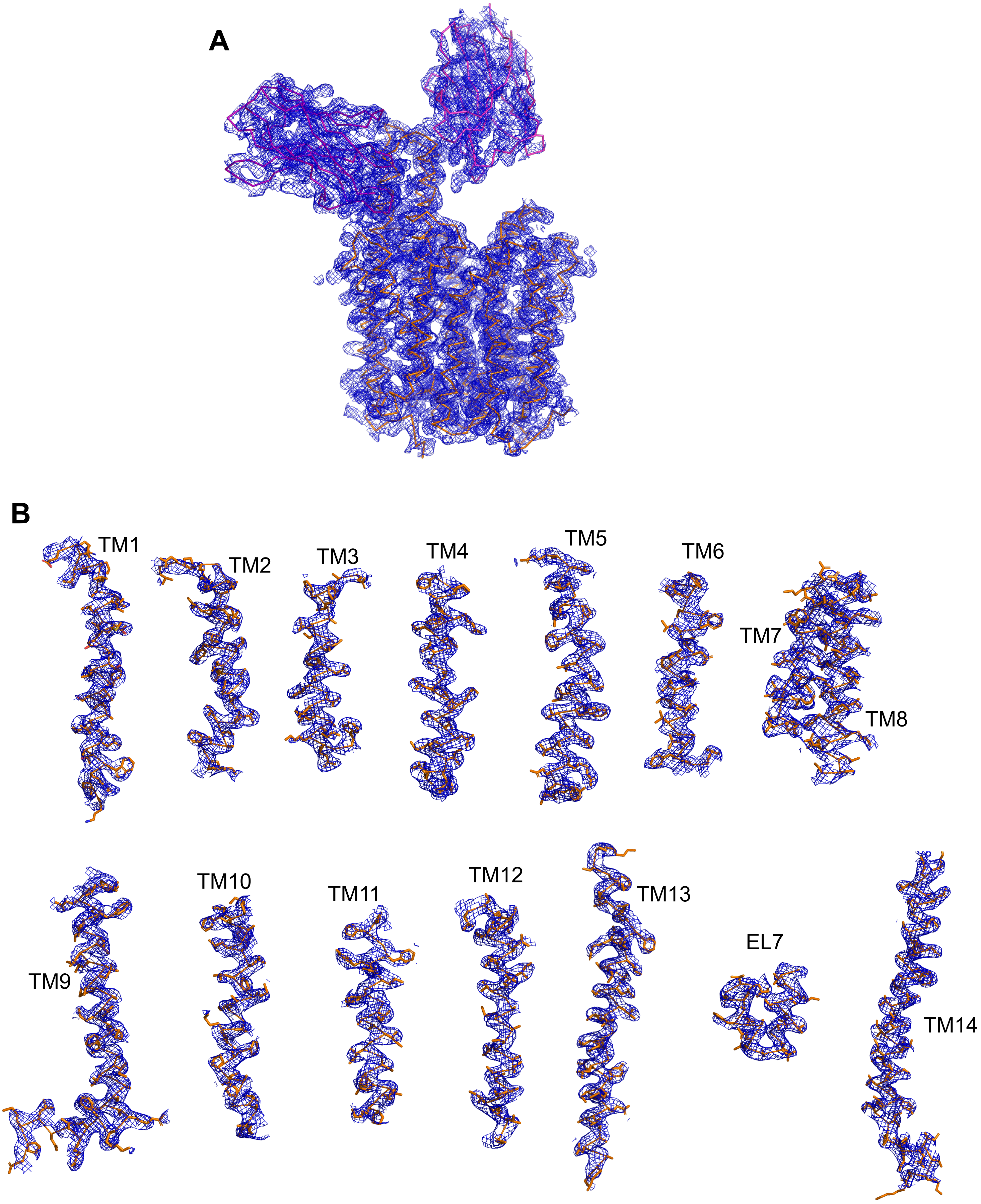

Appendix Figure S4 - Model fit for QacA-ICab complex and individual TM helices.
A, Coulomb potential map for QacA-ICabs complex contoured at 2σ.
B, individual transmembrane helices modelled.

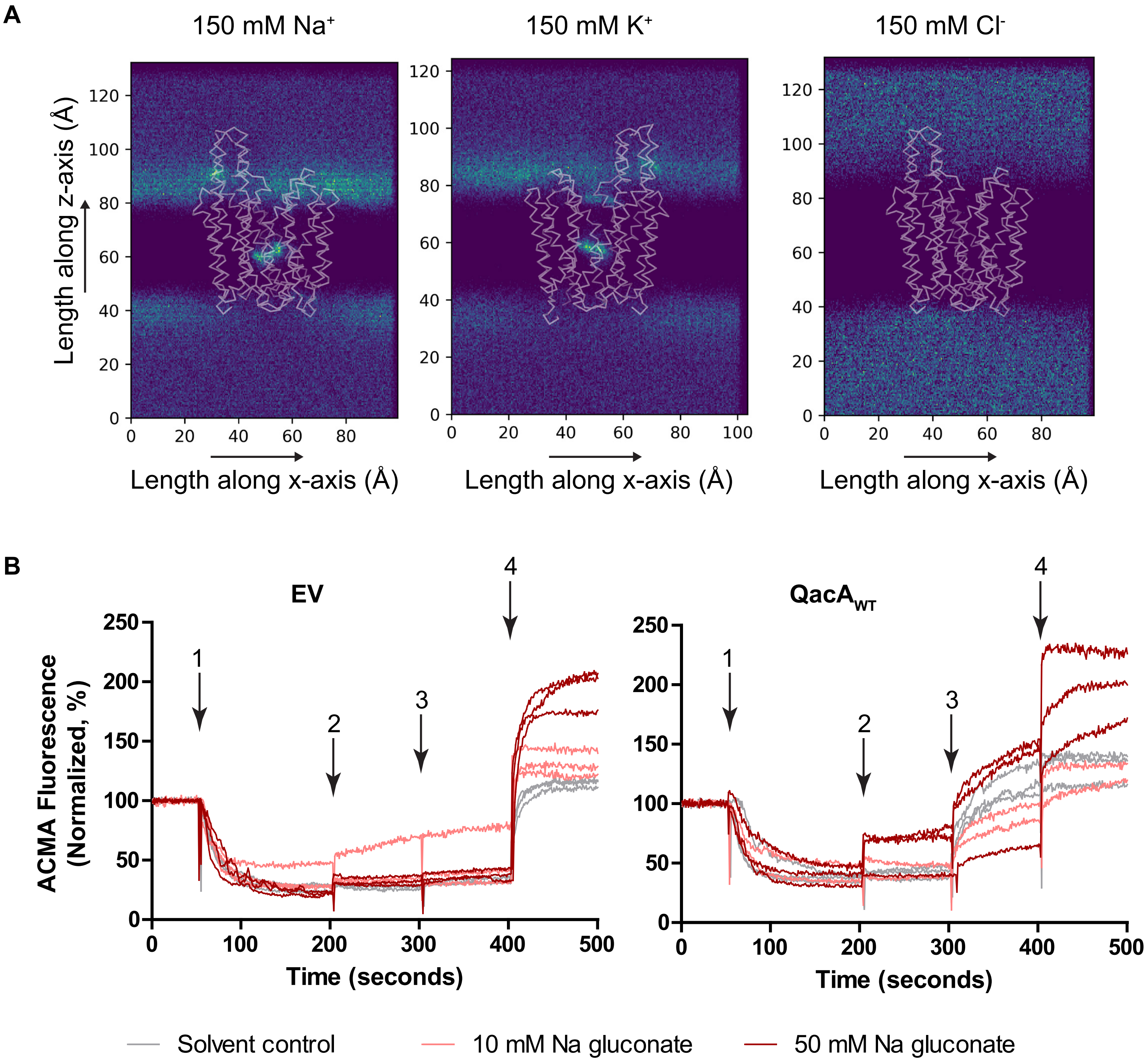

Appendix Figure S5 - Ion specificity in QacA's vestibule.

A, 2D heat map showing projection of a simulation run on xz plane. Individual dots represent anion/cation tracked in the trajectory. Dark band in the middle of Z axis represents lipid bilayer where Na^+/K^+/Cl^- ions could not enter. Bright spot in the centre of the box represents localization of Na^+/K^+ seen at the bottom of the solvent accessible vestibule of QacA. Atoms other than Na^+/K^+/Cl^- not shown for clarity. Averaged ribbon models of QacA are displayed on the maps to provide positional context.

B, Everted vesicles-based assay for sodium transport through $QacA_{WT}$. The vesicles were made using JD838 cells. The timepoints marked as 1-4 represent addition of 100 µM ATP, various concentrations of Sodium gluconate, 0.5 mM TPP and 2 µM Nigericin.

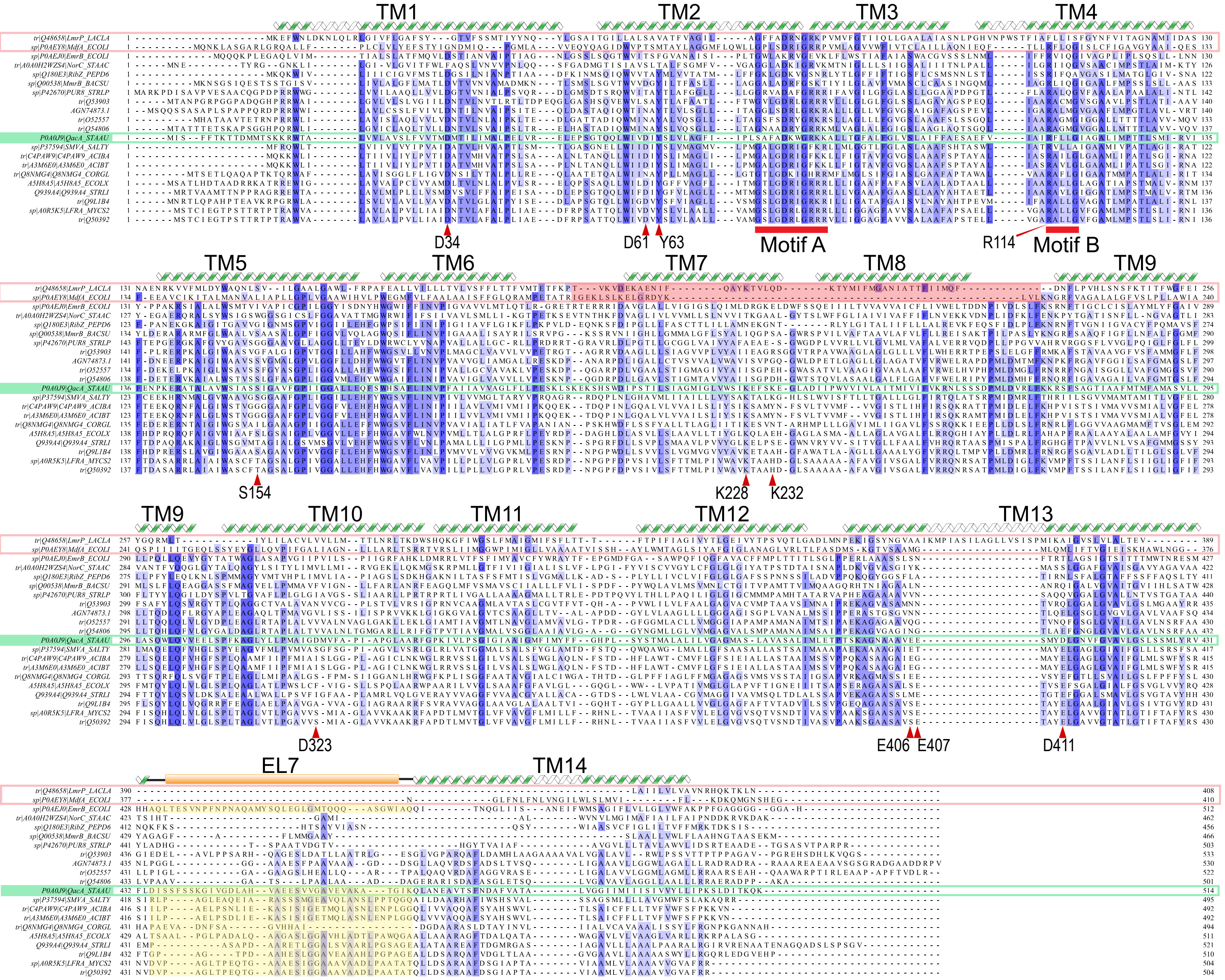

Appendix Figure S6 - Sequence and structural alignments of QacA with DHA1/2 transporters.
Multiple sequence alignment of QacA with related sequences from different prokaryotic genera. QacA is highlighted in green and DHA1 transporters MdfA and LmrP are boxed in red. Extents for transmembrane helices of QacA are shown above the MSA. Sequences for horizontal linker helix between TMs 6 and 7 in MdfA and LmrP, that substitute for linker TMs 7 and 8 in DHA2 transporters are highlighted in red, while the extracellular loop 7 (EL7) sequences in QacA homologs are highlighted in yellow. The conserved Motif A and Motif B are demarcated with red underline. Notable residues in the DHA2 family and with their residue positions in QacA are pointed using red arrows. Alignment was performed using clustal omega suite with 5 HMM iterations. Jalview was used for data visualization.

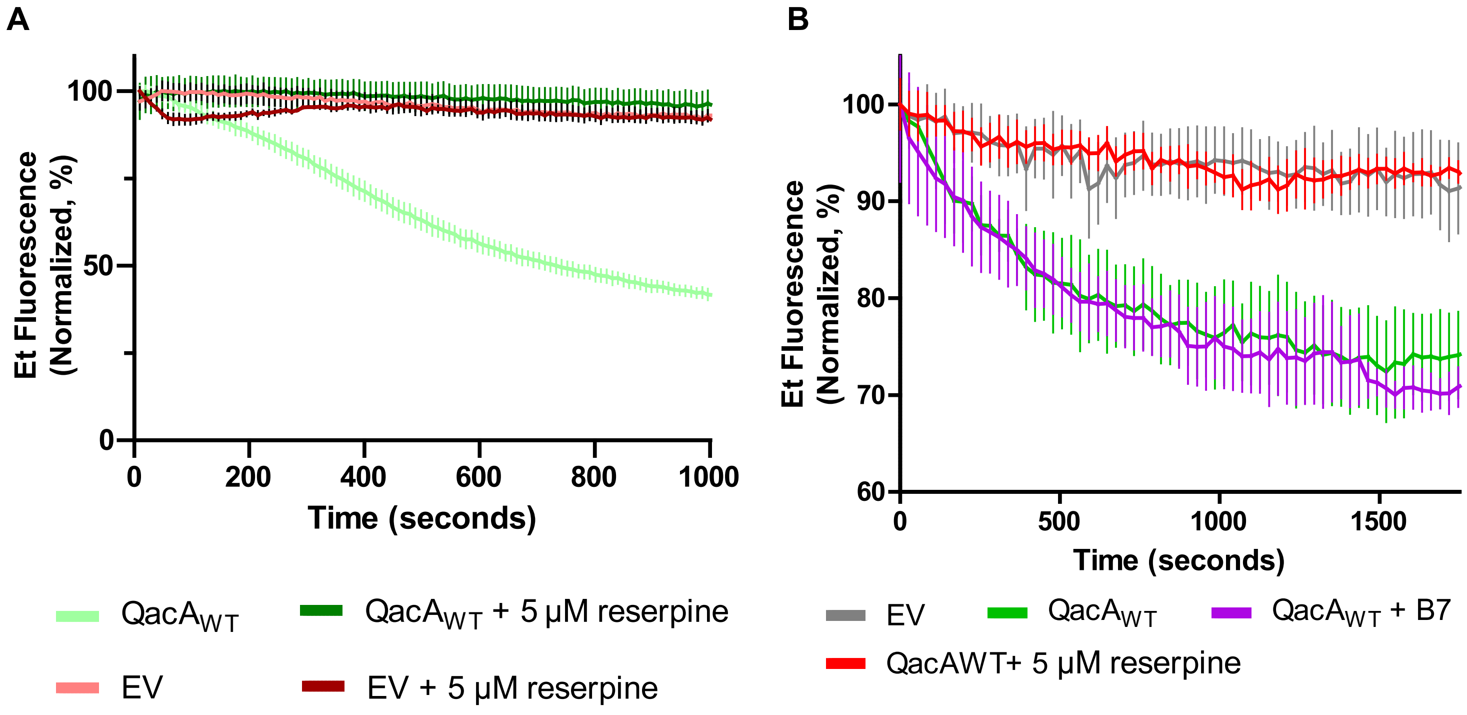

Appendix Figure S7 - Viability assay for spheroplasts based Ethidium efflux assay.
A, Whole cell-based efflux assay done in the presence of 5 µM reserpine to establish its inhibitory effect on $QacA_{WT}$ mediated ethidium efflux. Graph represents one of the two independent replicates performed for the assay. Error bars represent S.E.M. technical triplicates.
B, JD838 cells derived spheroplasts based ethidium efflux assay done in the presence of 5 µM reserpine. Graph represents one of the two independent replicates performed for this assay. Error bars depict S.E.M. for technical triplicates, and duplicates in case of just $QacA_{WT}$.

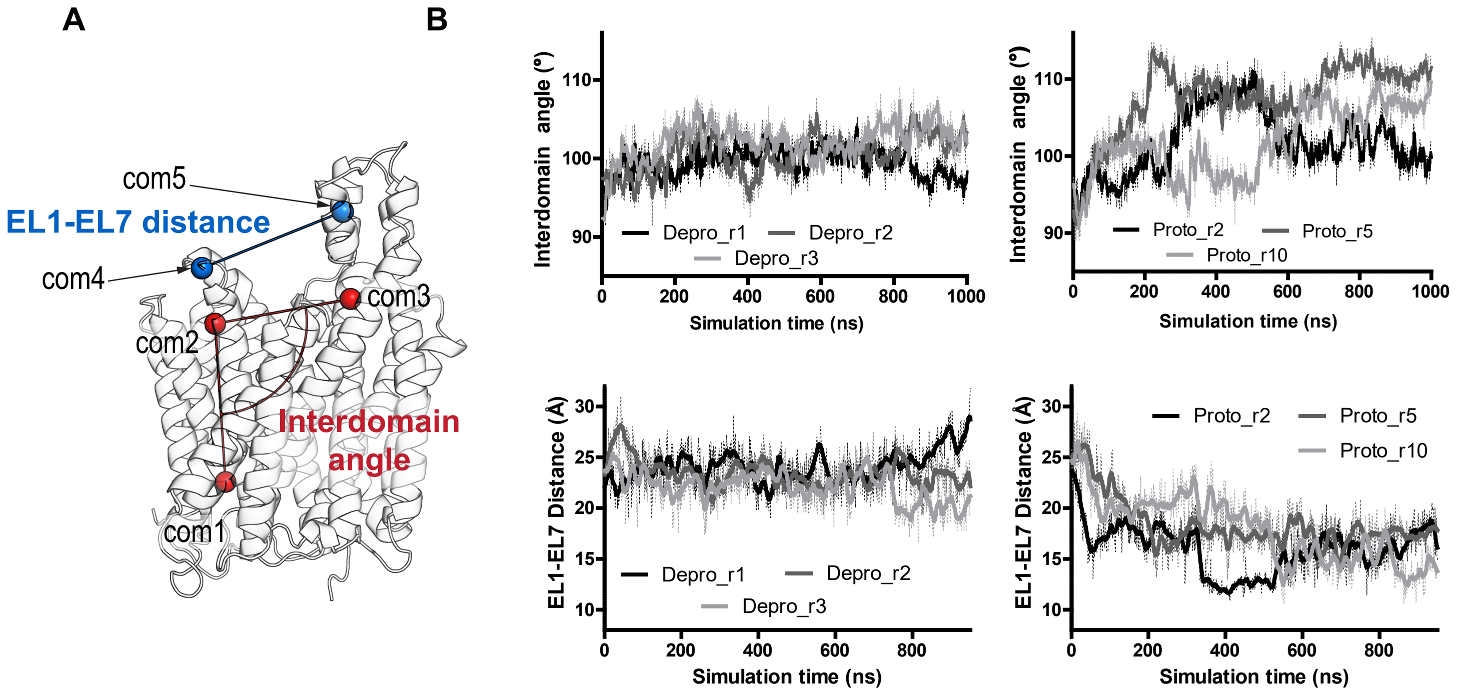

Appendix Figure S8 - Collective variables analyzed for various simulation runs on QacA.
A, A schematic of collective variables (CVs) measured in simulation trajectories. EL1-EL7 distances were calculated between com4 and com5, where they are the centres of mass of Cα atoms of residues R48 to T54, and I443 to T465 respectively. Interdomain angles broadly depict the domain motions of the transporter, where coms 1, 2 and 3 are the centres of mass of Cα atoms of L21, A78, L87 and T128; of L42, L57, V110 and F177; and of Q302, G313, S426 and F478 respectively.
B, Interdomain angle and EL1-EL7 distance trends in three representative runs each from QacA setup with either D411 deprotonated (negatively charged sidechain) or protonated (neutral sidechain).

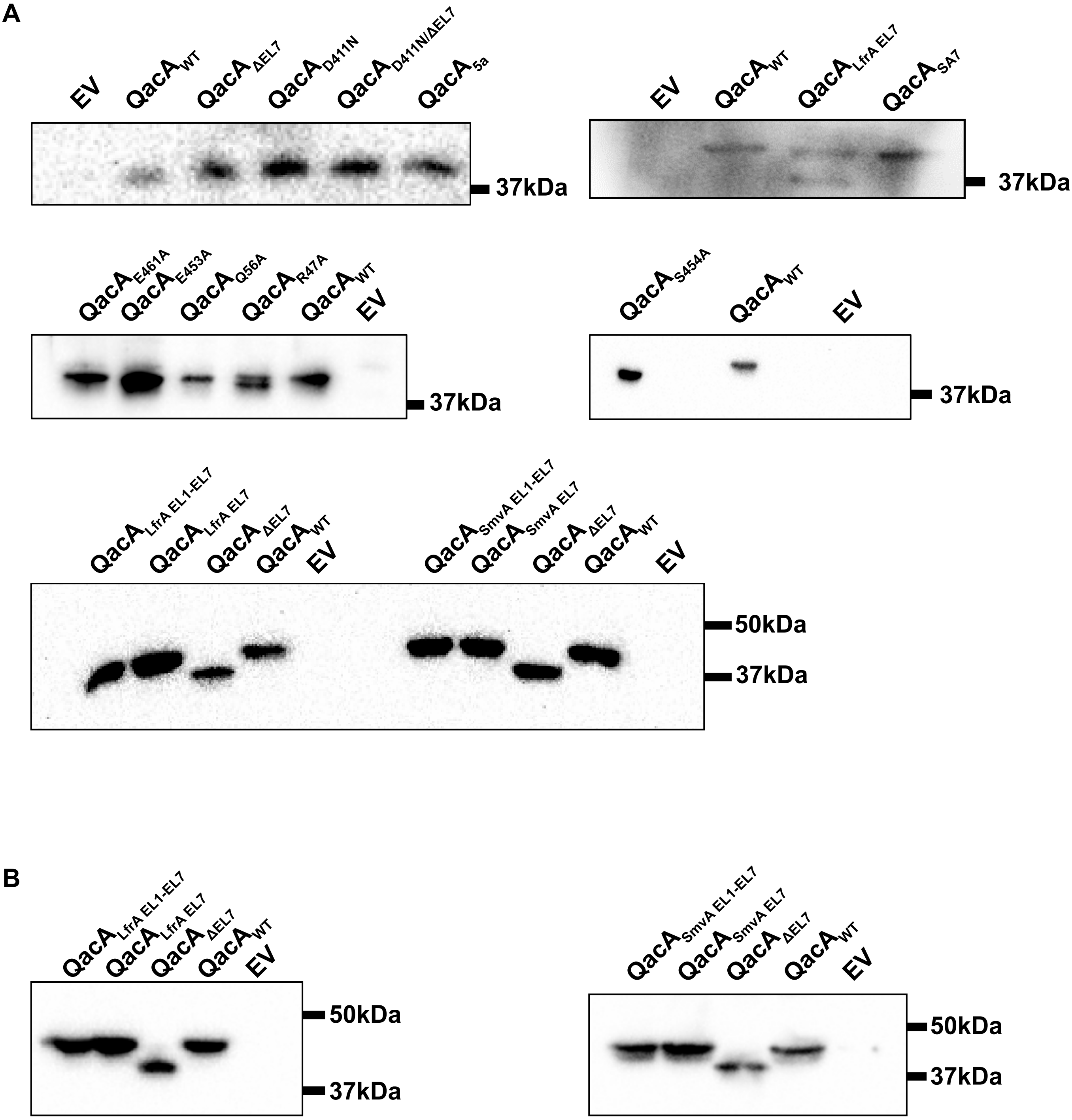

Appendix Figure S9 - Expression analysis of different constructs designed for functional studies on QacA using Western Blots.

The mutant constructs were cloned in pBAD vector with a C-terminal Histag. Expression of each construct was probed using Anti-His antibody and the signal was detected through HRP chemiluminescence.

A, Samples from whole cell-based ethidium efflux assay and spot-based survival assays directly loaded on SDS page.

B, Samples from everted vesicle-based transport assays. Samples (vesicle aliquots, which are devoid of cytoplasmic contents including inclusion bodies) were solubilized in 20 mM n-Undecyl-β-D-Maltopyranoside before loading on SDS page.

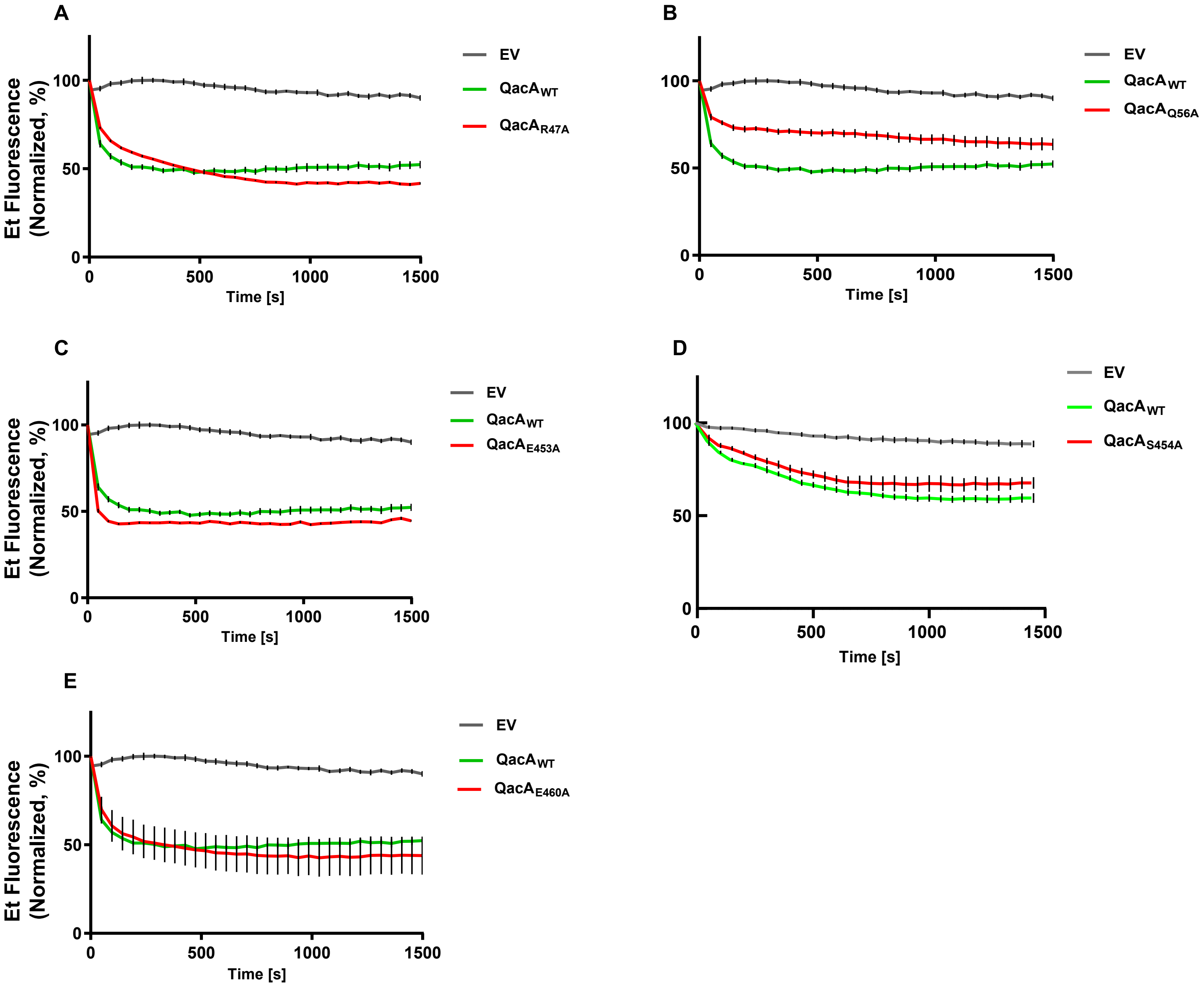

Appendix Figure S10 - Ethidium efflux assay of single alanine mutants at EL1-EL7 interface.
A to E Whole cell-based ethidium efflux assay with the interfacial-residue mutants to check the transport activity of individual residue forming the interface between EL1 and EL7 of the transporter by the means of alanine mutagenesis. n=6 for technical replicates; error bars represent S.E.M.

A QacA$_{R47A}$
B QacA$_{Q56A}$
C QacA$_{E453A}$
D QacA$_{S454A}$
E QacA$_{E460A}$

Appendix Table S1. CryoEM data collection, refinement and validation.

Parameters	QacA-ICab complex EMDB (EMD-33612) PDB id (7Y58)
**Data Collection/Processing**	
Microscope	FEI Titan Krios
Voltage (kV)	300
Detector	K3 Bioquantum
Magnification	105,000x
Defocus range	-1.0 – -4.0 (mm)
Pixel size	0.831
Electron exposure (e^-/Å^2)	51
Exposure time	2
Symmetry Imposed	C1
Initial particle number	5,723,316
Final particle number	218,040
Map Resolution	3.6
FSC threshold	0.143
**Refinement**	
Initial model	QacA Alphafold2 model
Map resolution (masked) (FSC 0.143)	3.8 Å
Map resolution (Density modification) (FSC 0.143)	3.6 Å
**Model Composition**	
Non-Hydrogen atoms	5609
Protein residues	743
Ligands	-
Mean B-factor (Å^2)	113.1
**RMS deviations**	
Bond lengths	0.003
Bond Angles	0.748
**Validation**	
Refined model CC	0.77
Molprobity score	2.12
Clashscore	14
**Ramachandran plot (%)**	
Favoured	90%
Allowed	10%
Disallowed	0
