# Supplementary material for: Cryo‐EM structure of antibacterial efflux transporter QacA from Staphylococcus aureus reveals a novel extracellular loop with allosteric role
Source: EMBO J. 2023 Jul 17;42(16):e113418. doi: 10.15252/embj.2023113418 (PMC10425836; doi:10.15252/embj.2023113418)
Supplement: Supplementary file 2 — Expanded View Figures PDF [file EMBJ-42-e113418-s002.pdf]

Expanded View Figures

Figure EV1. Characterization of affinities of ICabs against QacA.

A Flow cytometry derived apparent affinity measurements of ICabs A2 (top), B1 (middle) and B2 (bottom) for QacA. Error bars represent S.E.M. with $n = 3$ for technical triplicates.

B Population shift-based titrations of ICabs against QacA-GFP preincubated with other ICabs in the group. A rightward shift of population infers noncompetitive binding of ICabs.

C SEC profiles of ICabs A4 (left) and B7 (right). Collected fractions around 13–14 ml of elution volume were run on SDS–PAGE and the corresponding bands are shown (inset).

D FSEC profiles with leftward shifts in elution volumes of $QacA_{D411N}$ in the presence of A4 and B7 indicate increase in apparent molecular mass of QacA upon binding with either or both the ICabs.

Source data are available online for this figure.

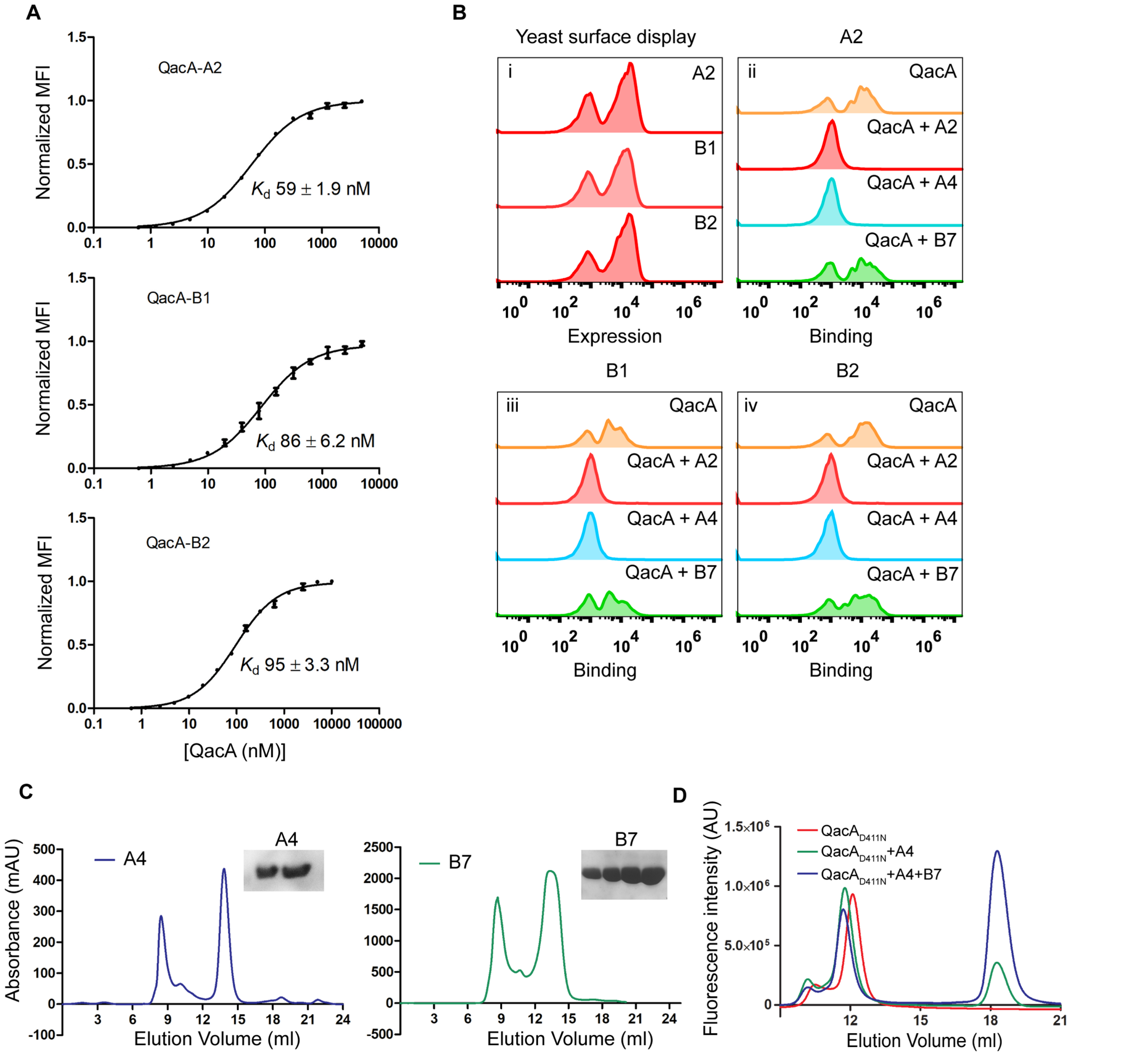

Figure EV1.

Figure EV2. Phylogeny of DHA2 members listed in the Transporter Classification Database.

Bootstrapped phylogeny derived from an evolutionary tree made using maximum likelihood method. Branching confidence is mentioned at each node. Each red or blue bar represents an acidic or basic residue, respectively, present in the vestibule of the transporter, as manually curated from their AlphaFold2 models.

Source data are available online for this figure.

 © 2023 The Authors

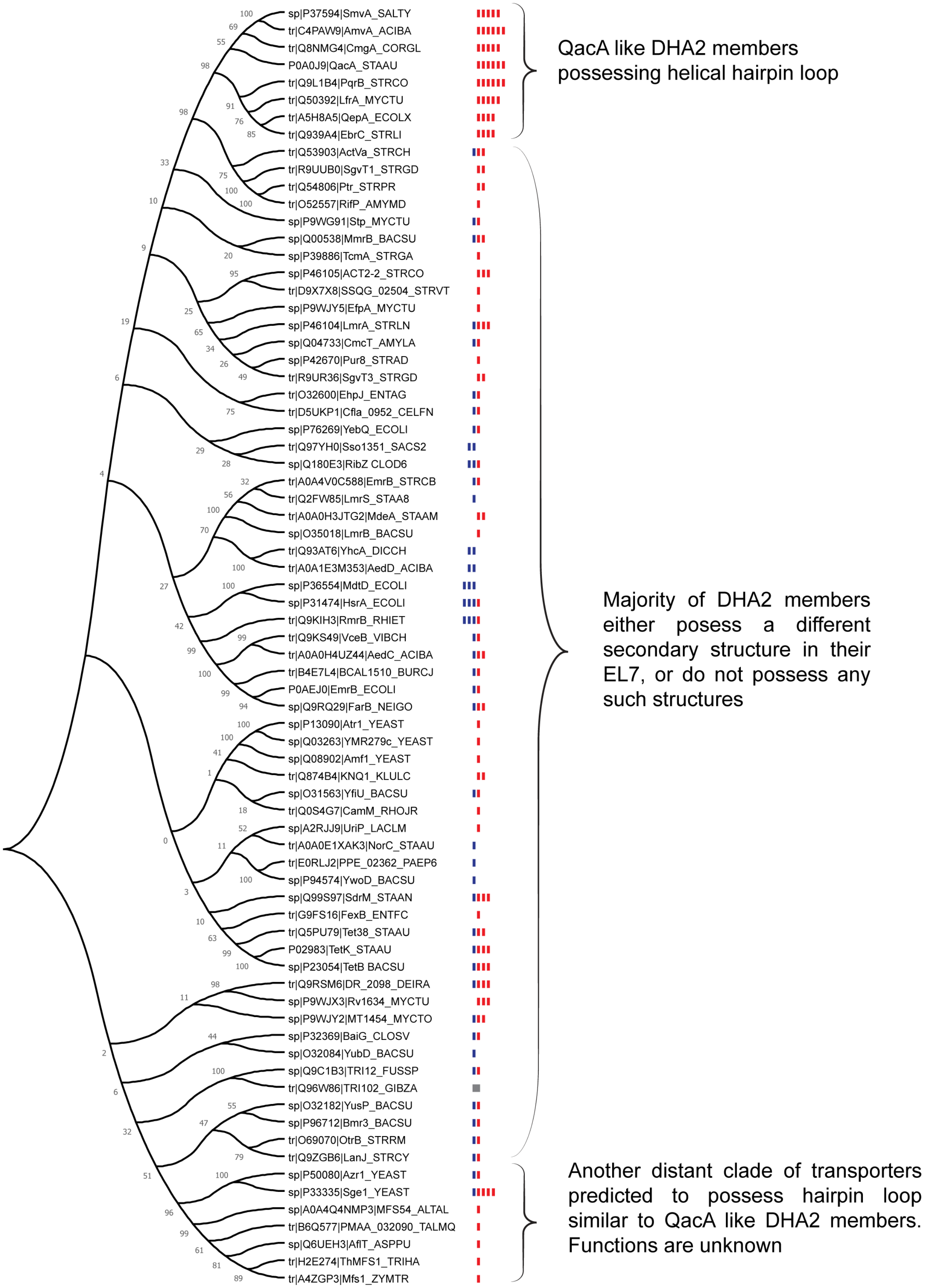
sp|P37594|SmvA_SALTY
tr|C4PAW9|AmvA_ACIBA
tr|Q8NMG4|CmgA_CORGL
P0A0J9|QacA_STAAU
tr|Q9L1B4|PqrB_STRCO
tr|Q50392|LfrA_MYCTU
tr|A5H8A5|QepA_ECOLX
tr|Q939A4|EbrC_STRLI
tr|Q53903|ActVa_STRCH
tr|R9UUB0|SgvT1_STRGD
tr|Q54806|Ptr_STRPR
tr|O52557|RifP_AMYMD
sp|P9WG91|Stp_MYCTU
sp|Q00538|MmrB_BACSU
sp|P39886|TcmA_STRGA
sp|P46105|ACT2-2_STRCO
tr|D9X7X8|SSQG_02504_STRVT
sp|P9WJY5|EfpA_MYCTU
sp|P46104|LmrA_STRLN
sp|Q04733|CmcT_AMYLA
sp|P42670|Pur8_STRAD
tr|R9UR36|SgvT3_STRGD
tr|O32600|EhpJ_ENTAG
tr|D5UKP1|Cfla_0952_CELFN
sp|P76269|YebQ_ECOLI
tr|Q97YH0|Sso1351_SACS2
sp|Q180E3|RibZ CLOD6
tr|A0A4V0C588|EmrB_STRCB
tr|Q2FW85|LmrS_STAA8
tr|A0A0H3JTG2|MdeA_STAAM
sp|O35018|LmrB_BACSU
tr|Q93AT6|YhcA_DICCH
tr|A0A1E3M353|AedD_ACIBA
sp|P36554|MdtD_ECOLI
sp|P31474|HsrA_ECOLI
tr|Q9KIH3|RmrB_RHIET
tr|Q9KS49|VceB_VIBCH
tr|A0A0H4UZ44|AedC_ACIBA
tr|B4E7L4|BCAL1510_BURCJ
P0AEJ0|EmrB_ECOLI
sp|Q9RQ29|FarB_NEIGO
sp|P13090|Atr1_YEAST
sp|Q03263|YMR279c_YEAST
sp|Q08902|Amf1_YEAST
tr|Q874B4|KNQ1_KLULC
sp|O31563|YfiU_BACSU
tr|Q0S4G7|CamM_RHOJR
sp|A2RJJ9|UriP_LACLM
tr|A0A0E1XAK3|NorC_STAAU
tr|E0RLJ2|PPE_02362_PAEP6
sp|P94574|YwoD_BACSU
sp|Q99S97|SdrM_STAAN
tr|G9FS16|FexB_ENTFC
tr|Q5PU79|Tet38_STAAU
P02983|TetK_STAAU
sp|P23054|TetB BACSU
tr|Q9RSM6|DR_2098_DEIRA
sp|P9WJX3|Rv1634_MYCTU
sp|P9WJY2|MT1454_MYCTO
sp|P32369|BaiG_CLOSV
sp|O32084|YubD_BACSU
sp|Q9C1B3|TRI12_FUSSP
tr|Q96W86|TRI102_GIBZA
sp|O32182|YusP_BACSU
sp|P96712|Bmr3_BACSU
tr|O69070|OtrB_STRRM
tr|Q9ZGB6|LanJ_STRCY
sp|P50080|Azr1_YEAST
sp|P33335|Sge1_YEAST
sp|A0A4Q4NMP3|MFS54_ALTAL
tr|B6Q577|PMAA_032090_TALMQ
sp|Q6UEH3|AflT_ASPPU
tr|H2E274|ThMFS1_TRIHA
tr|A4ZGP3|Mfs1_ZYMTR
QacA like DHA2 members possessing helical hairpin loop
Majority of DHA2 members either posess a different secondary structure in their EL7, or do not possess any such structures
Another distant clade of transporters predicted to possess hairpin loop similar to QacA like DHA2 members. Functions are unknown

Figure EV2.

Figure EV3. General overview of QacA-ICab interfaces and their inhibition propensities.

A Structural overview of $QacA_{D411N}$-A4-B7 complex with the globular domains of ICabs shown as gray cartoons, and the CDRs 1, 2 and 3 are shown in red, green, and yellow colors, respectively. QacA is shown in blue helices.

B QacA-A4 interface shown with participating residues in sticks. For the residues that are involved in forming polar/H-bond interactions through their main-chain atoms only, the sidechains are shown as thin lines, for clarity. Interacting residues from A4 and QacA are labeled in gray and black fonts and depicted in gray and blue sticks, respectively. H-bond interactions are shown as dotted lines. Color scheme follows panel A.

C QacA-B7 interface shown in the same convention as panel (B).

D Spheroplasts-based ethidium efflux assay shown as traces normalized between 100 and 0% to first reading in each sample and zero fluorescence, respectively. Data shown here are from a single biological replicate. Error bars represent SEM obtained from six technical replicates.

E Superposition of $QacA_{io}$ model with $QacA_{D411N}$-B7 coordinates structurally aligned at the EL7 region. A probable clash between CDR2 (green cartoon) and EL1 (opaque blue cartoon) observed through rigid fitting is shown as the circled ROI.

Source data are available online for this figure.

© 2023 The Authors

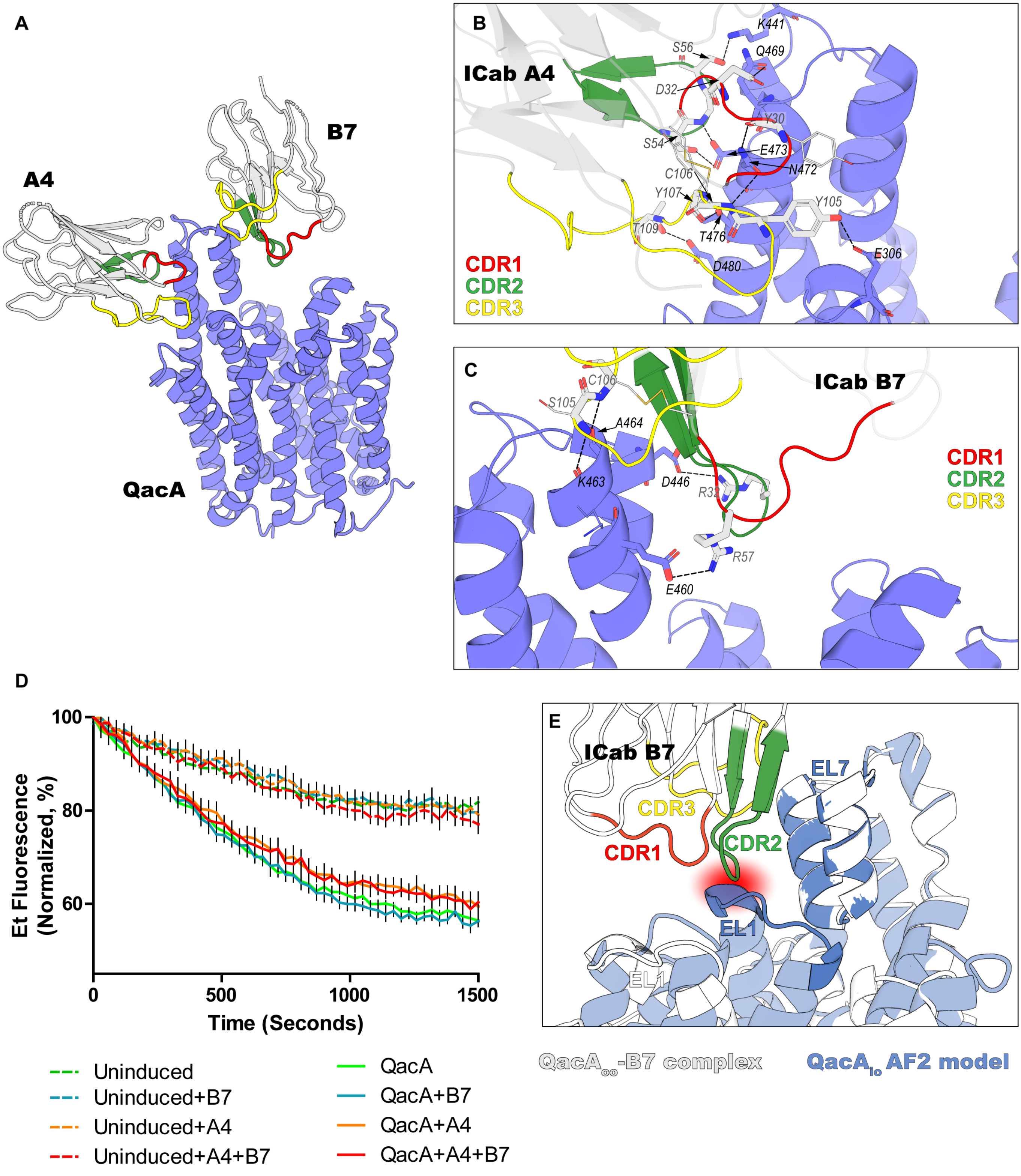
A
B7
A4
QacA
B
ICab A4
K441
S56
Q469
D32
Y30
S54
E473
N472
C106
Y107
Y105
T109
T476
E306
D480
CDR1
CDR2
CDR3
C
C106
ICab B7
S105
A464
CDR1
CDR2
CDR3
K463
D446
R57
E460
D
100
80
60
Et Fluorescence (Normalized, %)
0
500
1000
1500
Time (Seconds)
Uninduced
Uninduced+B7
Uninduced+A4
Uninduced+A4+B7
QacA
QacA+B7
QacA+A4
QacA+A4+B7
E
ICab B7
EL7
CDR3
CDR2
CDR1
EL1
EL1
QacA$_{oo}$-B7 complex
QacA$_{io}$ AF2 model

Figure EV3.

Figure EV4. Structure similarity of EL1 and EL7 regions in QacA homologs.

A Structural similarity between LfrA, SmvA, and QacA ELs 1 and 7 shown using their AlphaFold2 models. Identical residues are shown in transparent spheres.

B Everted vesicle traces (technical triplicates shown for 1 biological replicate analyzed in Fig 5J) for one of three biological replicates reported in this study. Fluorescence is normalized between data recorded between 40 and 50 s of acquisition and zero absolute fluorescence intensity.

C Histogram showing normalized ACMA fluorescence recovery during everted vesicle-based transport assays. Data points overlaid are mean values of technical triplicates, for three biological replicates. Error bars represent SEM. Unpaired *t*-test conducted assuming Gaussian distribution and equal variance across constructs gave the following *P*-values: 0.001 for EV vs $QacA_{WT}$; 0.002 for $QacA_{WT}$ vs $QacA_{\Delta EL7}$; 0.0275 for $QacA_{\Delta EL7}$ vs $QacA_{LfrA\ EL7}$; 0.0015 for $QacA_{\Delta EL7}$ vs $QacA_{LfrA\ EL1\text{-}EL7}$; 0.004 for $QacA_{\Delta EL7}$ vs $QacA_{SmvA\ EL1\text{-}EL7}$; < 0.0001 for $QacA_{SmvA\ EL7}$ vs $QacA_{SmvA\ EL1\text{-}EL7}$. $*P < 0.05$, $**P < 0.01$, $***P < 0.001$.

Source data are available online for this figure.

 © 2023 The Authors

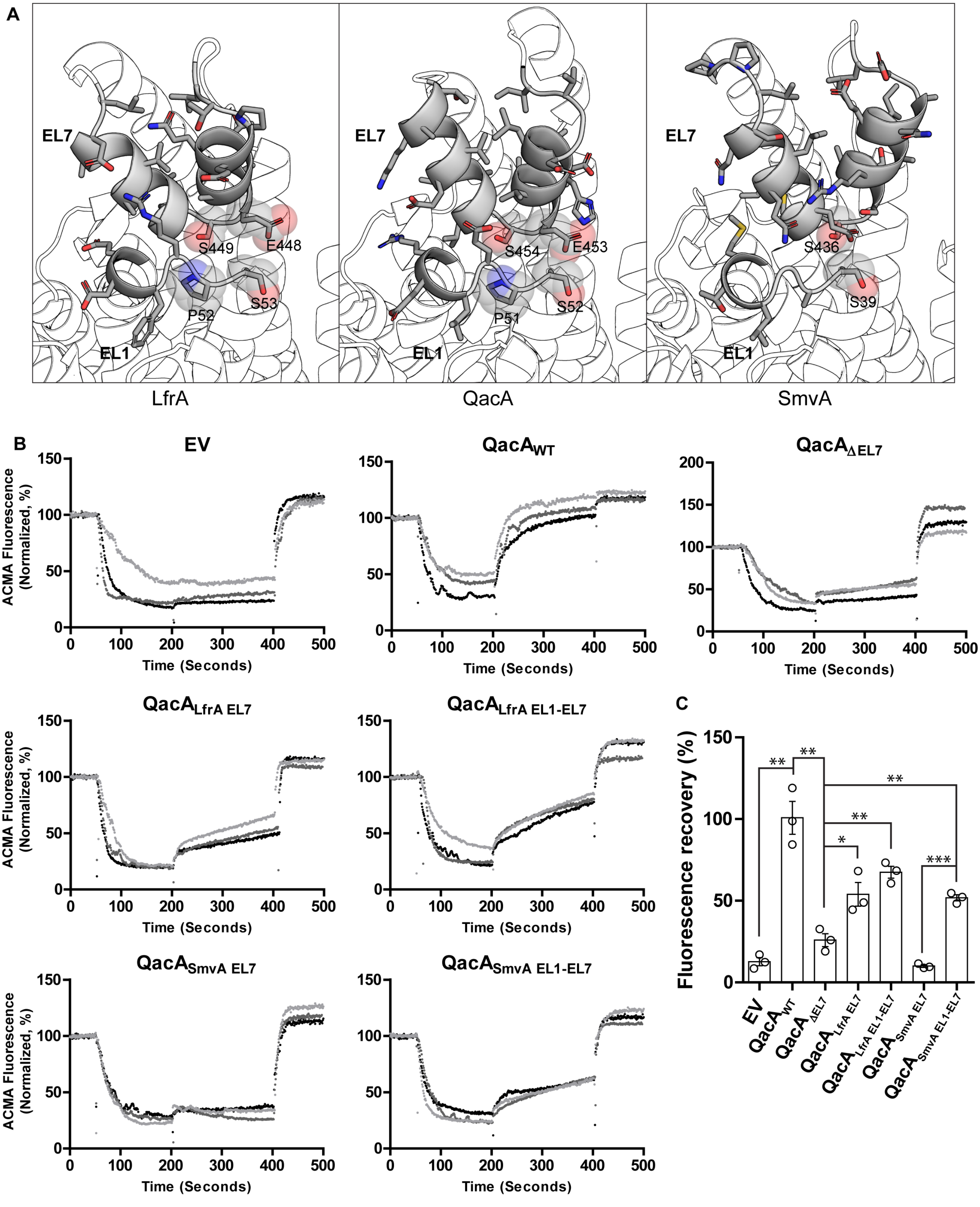
A
EL7
S449
E448
P52
S53
EL1
LfrA
EL7
S454
E453
P51
S52
EL1
QacA
EL7
S436
S39
EL1
SmvA
B
EV
QacA_WT
QacA_ΔEL7
QacA_LfrA EL7
QacA_LfrA EL1-EL7
QacA_SmvA EL7
QacA_SmvA EL1-EL7
ACMA Fluorescence (Normalized, %)
Time (Seconds)
0
50
100
150
200
300
400
500
C
Fluorescence recovery (%)
**
**
**
**
*
***
EV
QacA_WT
QacA_ΔEL7
QacA_LfrA EL7
QacA_LfrA EL1-EL7
QacA_SmvA EL7
QacA_SmvA EL1-EL7

Figure EV4.

Figure EV5. Presence of asymmetric rocker-switch motion in QacA.

A The Cα displacement vectors (blue dashes) between aligned $QacA_{D411N}$ ($QacA_{oo}$) structure and $QacA_{io}$ model show axis of rotation traversing along and through the middle of the bilayer to depict asymmetry between the degree of motions between the N-terminal and C-terminal domains.

B Overlay of $QacA_{oo}$ and $QacA_{io}$ coordinates with $QacA_{oo}$ contoured in the background for positional context. Helices shown in opaque or corresponding faded shades depict individual segments of $QacA_{io}$ and $QacA_{oo}$ states, respectively. A rainbow color scheme is followed from N- to the C-terminal end of QacA.

C Cα displacement values are shown for structurally aligned inward- and outward-open states of QacA, MdfA and LacY, with extents of N- and C-terminal domains, TMs 7-8 or ICH, and EL7 annotated. Models used for these alignments are $QacA_{D411N}$ cryo-EM structure and QacA AF2 model, PDBIDs 6GV1 (MdfA outward open) and 4ZP0 (MdfA inward open), and PDBIDs 5GXB (LacY outward open) and 1PV7 (LacY inward open).

D Interdomain angle (left) and EL1-EL7 distance (right) traces extracted from one of the simulation runs having D411 sidechain neutral (protonated). One frame from 408th ns was used to depict $QacA_{occ}$ conformation. Solid lines are smoothened projections of real trace (shown as dashed gray trace). Y-intercept in both the graphs represents the values of these CVs in $QacA_{io}$ model.

E Snapshots (ribbons in greyscale) of 10 equally distributed frames between 300 and 500 ns of trajectory of the run described in panel (E). Small bunches of helices are shown in multiple panels for clarity. Major displacements from $QacA_{oo}$ state are marked with arrows. A cartoon outline of $QacA_{D411N}$ is displayed behind the helices to provide positional context. Since the comparison is made with an ensemble, displacements are not quantified in the panels.

Source data are available online for this figure.

 © 2023 The Authors

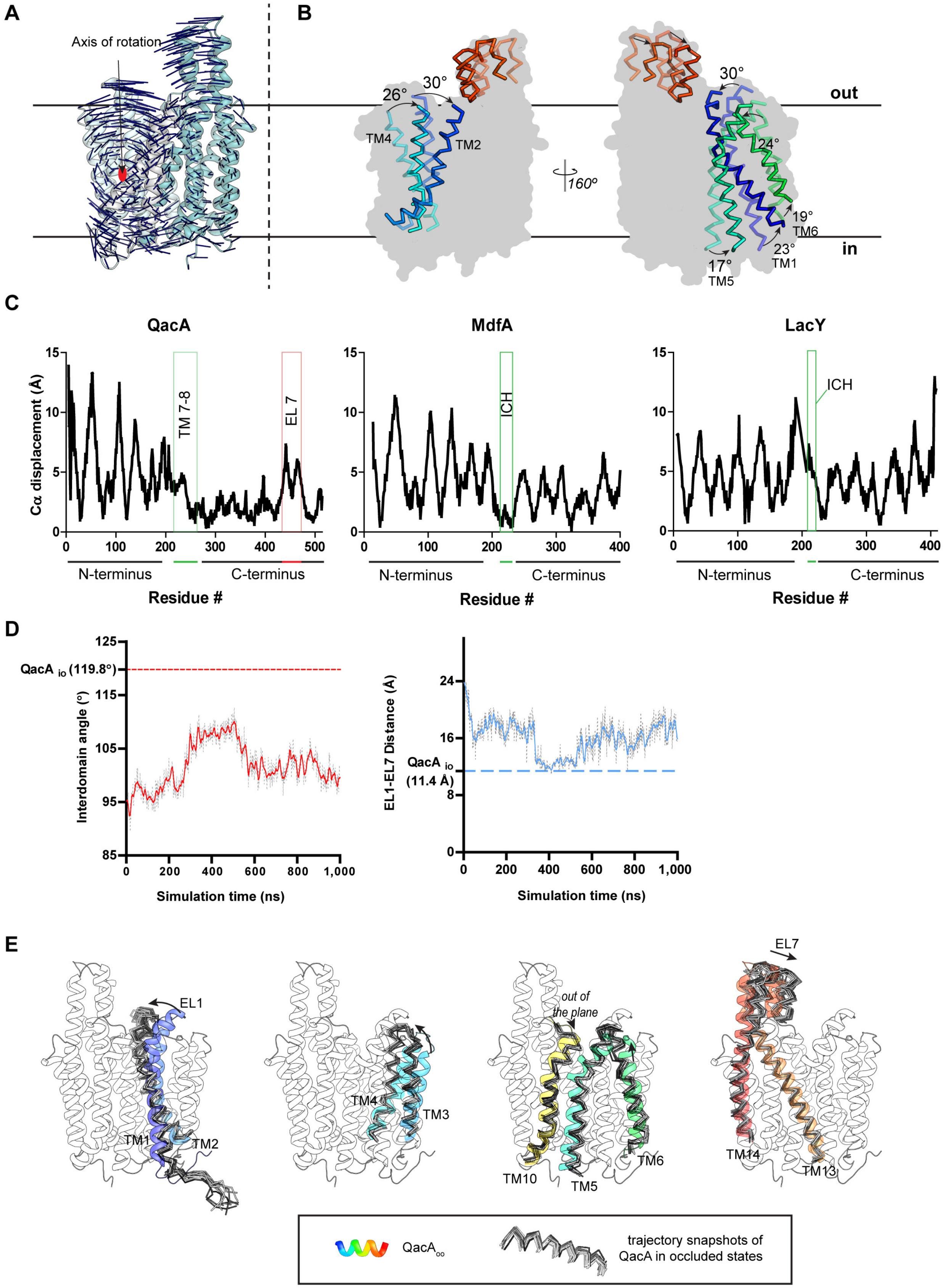
A
Axis of rotation
B
26°
30°
TM4
TM2
160°
30°
24°
19°
TM6
23°
TM1
17°
TM5
out
in
C
QacA
MdfA
LacY
Cα displacement (Å)
TM 7-8
EL 7
ICH
N-terminus
C-terminus
Residue #
D
QacA io (119.8°)
Interdomain angle (°)
Simulation time (ns)
EL1-EL7 Distance (Å)
QacA io (11.4 Å)
E
EL1
TM1
TM2
TM4
TM3
out of the plane
TM10
TM5
TM6
EL7
TM14
TM13
QacAoo
trajectory snapshots of QacA in occluded states

Figure EV5.
