# Supplementary figures and images for: Cryo‐EM structure of antibacterial efflux transporter QacA from Staphylococcus aureus reveals a novel extracellular loop with allosteric role
Source: EMBO J. 2023 Jul 17;42(16):e113418. doi: 10.15252/embj.2023113418 (PMC10425836; doi:10.15252/embj.2023113418)

A4 SDS PAGE

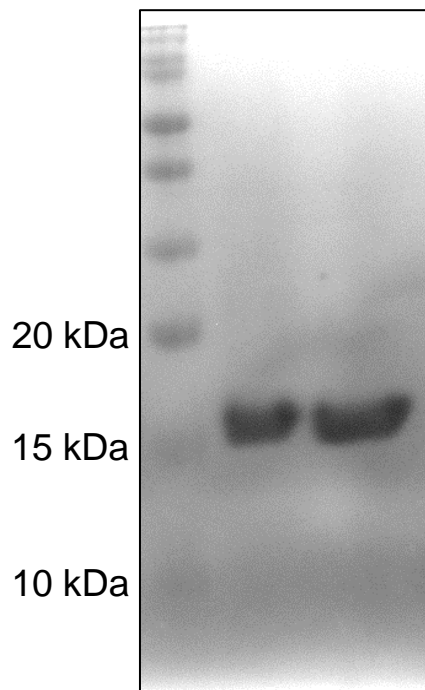

B7 SDS PAGE

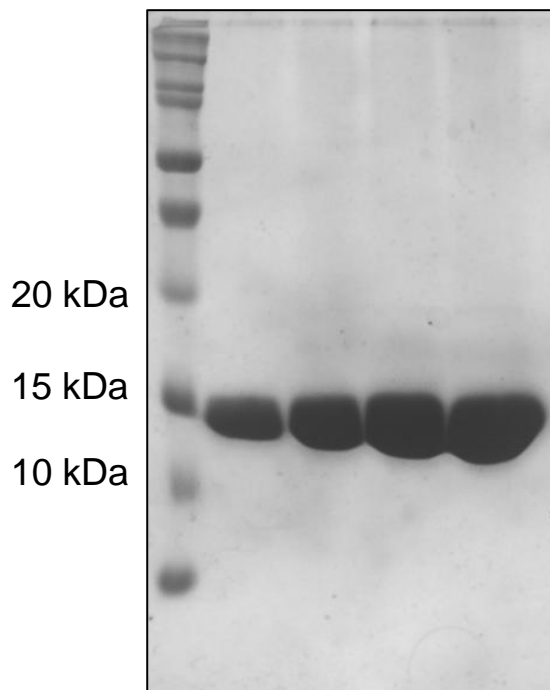

SDS PAGE gels from Figure EV1C

Supplement: Supplementary file 4 — Source Data for Expanded View [file EMBJ-42-e113418-s008.zip › Expanded view source data/Figure EV1/EV1C.pdf]

**Figure 1C spot assays**

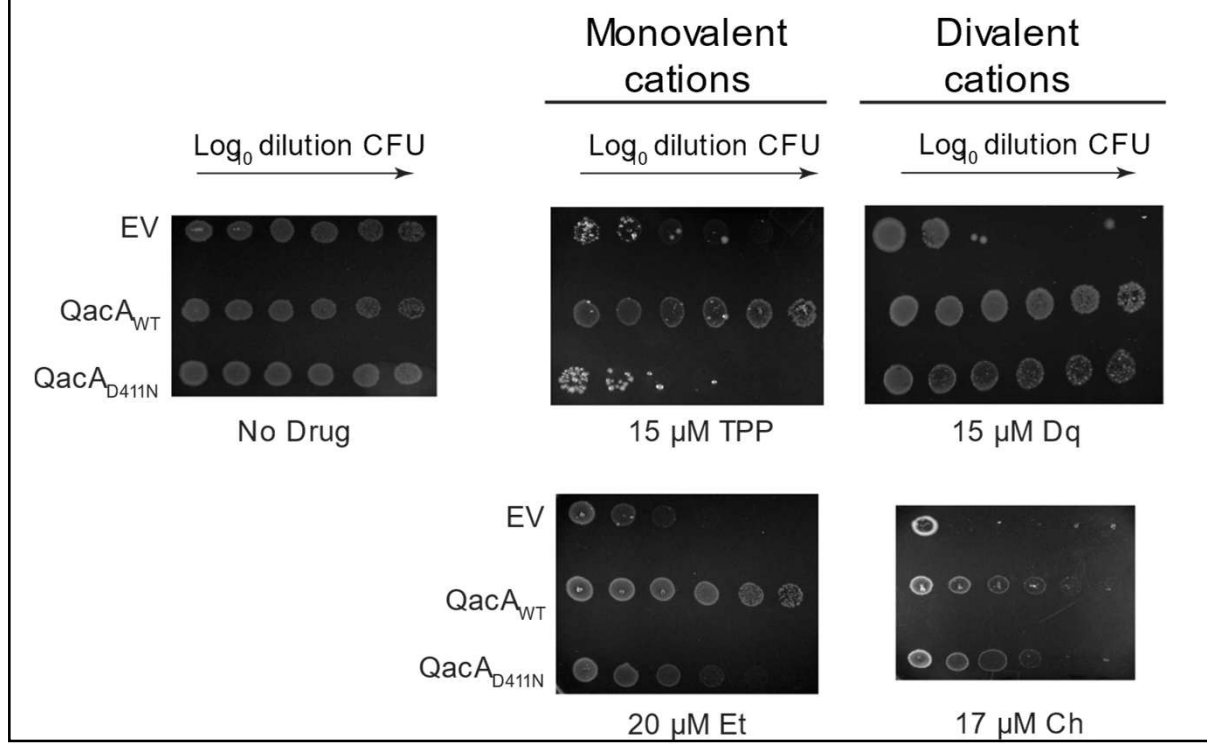

Supplement: Supplementary file 6 — Source Data for Figure 1 [file EMBJ-42-e113418-s005.zip › Main Figure 1/Figure 1C/Figure 1C Spot assay.pdf]

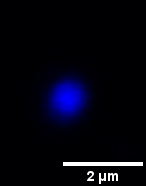

Supplement: Supplementary file 6 — Source Data for Figure 1 [file EMBJ-42-e113418-s005.zip › Main Figure 1/Figure 1G/B7 localization/processed/induced/cropped/cell1 image7_DAPI.png]

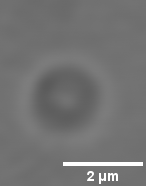

Supplement: Supplementary file 6 — Source Data for Figure 1 [file EMBJ-42-e113418-s005.zip › Main Figure 1/Figure 1G/B7 localization/processed/induced/cropped/cell1 image7_DIC.png]

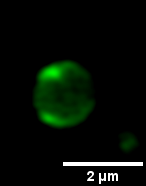

Supplement: Supplementary file 6 — Source Data for Figure 1 [file EMBJ-42-e113418-s005.zip › Main Figure 1/Figure 1G/B7 localization/processed/induced/cropped/cell1 image7_GFP.png]

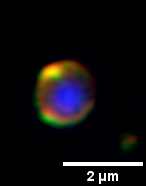

Supplement: Supplementary file 6 — Source Data for Figure 1 [file EMBJ-42-e113418-s005.zip › Main Figure 1/Figure 1G/B7 localization/processed/induced/cropped/cell1 image7_merged.png]

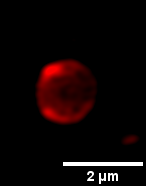

Supplement: Supplementary file 6 — Source Data for Figure 1 [file EMBJ-42-e113418-s005.zip › Main Figure 1/Figure 1G/B7 localization/processed/induced/cropped/cell1 image7_rhodamine.png]

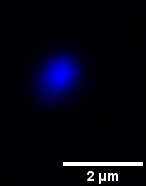

Supplement: Supplementary file 6 — Source Data for Figure 1 [file EMBJ-42-e113418-s005.zip › Main Figure 1/Figure 1G/B7 localization/processed/induced/cropped/cell1 image9_DAPI.png]

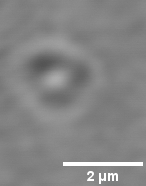

Supplement: Supplementary file 6 — Source Data for Figure 1 [file EMBJ-42-e113418-s005.zip › Main Figure 1/Figure 1G/B7 localization/processed/induced/cropped/cell1 image9_DIC.png]

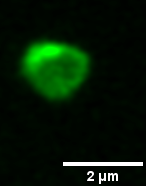

Supplement: Supplementary file 6 — Source Data for Figure 1 [file EMBJ-42-e113418-s005.zip › Main Figure 1/Figure 1G/B7 localization/processed/induced/cropped/cell1 image9_GFP.png]

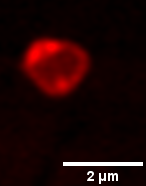

Supplement: Supplementary file 6 — Source Data for Figure 1 [file EMBJ-42-e113418-s005.zip › Main Figure 1/Figure 1G/B7 localization/processed/induced/cropped/cell1 image9_rhodamine.png]

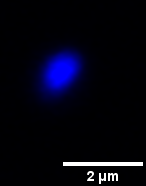

Supplement: Supplementary file 6 — Source Data for Figure 1 [file EMBJ-42-e113418-s005.zip › Main Figure 1/Figure 1G/B7 localization/processed/induced/cropped/cell2 image7_DAPI.png]

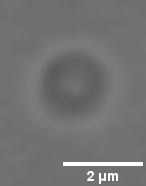

Supplement: Supplementary file 6 — Source Data for Figure 1 [file EMBJ-42-e113418-s005.zip › Main Figure 1/Figure 1G/B7 localization/processed/induced/cropped/cell2 image7_DIC.png]

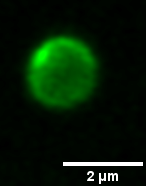

Supplement: Supplementary file 6 — Source Data for Figure 1 [file EMBJ-42-e113418-s005.zip › Main Figure 1/Figure 1G/B7 localization/processed/induced/cropped/cell2 image7_GFP.png]

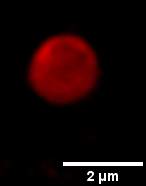

Supplement: Supplementary file 6 — Source Data for Figure 1 [file EMBJ-42-e113418-s005.zip › Main Figure 1/Figure 1G/B7 localization/processed/induced/cropped/cell2 image7_rhodamine.png]

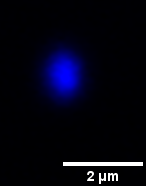

Supplement: Supplementary file 6 — Source Data for Figure 1 [file EMBJ-42-e113418-s005.zip › Main Figure 1/Figure 1G/B7 localization/processed/induced/cropped/cell2 image9_DAPI.png]

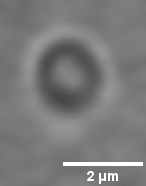

Supplement: Supplementary file 6 — Source Data for Figure 1 [file EMBJ-42-e113418-s005.zip › Main Figure 1/Figure 1G/B7 localization/processed/induced/cropped/cell2 image9_DIC.png]

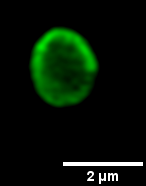

Supplement: Supplementary file 6 — Source Data for Figure 1 [file EMBJ-42-e113418-s005.zip › Main Figure 1/Figure 1G/B7 localization/processed/induced/cropped/cell2 image9_GFP.png]

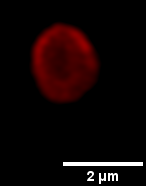

Supplement: Supplementary file 6 — Source Data for Figure 1 [file EMBJ-42-e113418-s005.zip › Main Figure 1/Figure 1G/B7 localization/processed/induced/cropped/cell2 image9_rhodamine.png]

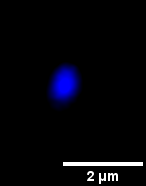

Supplement: Supplementary file 6 — Source Data for Figure 1 [file EMBJ-42-e113418-s005.zip › Main Figure 1/Figure 1G/B7 localization/processed/induced/cropped/cell3 image7_DAPI.png]

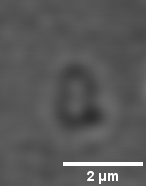

Supplement: Supplementary file 6 — Source Data for Figure 1 [file EMBJ-42-e113418-s005.zip › Main Figure 1/Figure 1G/B7 localization/processed/induced/cropped/cell3 image7_DIC.png]

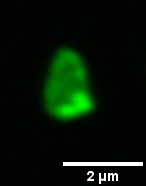

Supplement: Supplementary file 6 — Source Data for Figure 1 [file EMBJ-42-e113418-s005.zip › Main Figure 1/Figure 1G/B7 localization/processed/induced/cropped/cell3 image7_GFP.png]

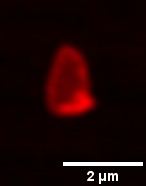

Supplement: Supplementary file 6 — Source Data for Figure 1 [file EMBJ-42-e113418-s005.zip › Main Figure 1/Figure 1G/B7 localization/processed/induced/cropped/cell3 image7_rhodamine.png]

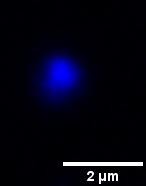

Supplement: Supplementary file 6 — Source Data for Figure 1 [file EMBJ-42-e113418-s005.zip › Main Figure 1/Figure 1G/B7 localization/processed/induced/cropped/cell3 image9_DAPI.png]

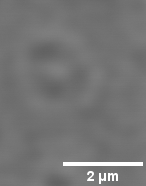

Supplement: Supplementary file 6 — Source Data for Figure 1 [file EMBJ-42-e113418-s005.zip › Main Figure 1/Figure 1G/B7 localization/processed/induced/cropped/cell3 image9_DIC.png]

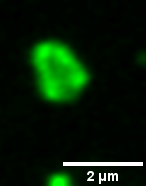

Supplement: Supplementary file 6 — Source Data for Figure 1 [file EMBJ-42-e113418-s005.zip › Main Figure 1/Figure 1G/B7 localization/processed/induced/cropped/cell3 image9_GFP.png]

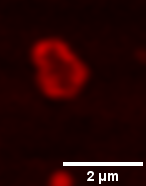

Supplement: Supplementary file 6 — Source Data for Figure 1 [file EMBJ-42-e113418-s005.zip › Main Figure 1/Figure 1G/B7 localization/processed/induced/cropped/cell3 image9_rhodamine.png]

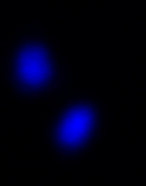

Supplement: Supplementary file 6 — Source Data for Figure 1 [file EMBJ-42-e113418-s005.zip › Main Figure 1/Figure 1G/B7 localization/processed/induced/cropped/cell4 image7_DAPI.png]

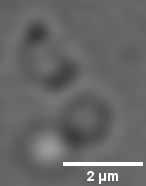

Supplement: Supplementary file 6 — Source Data for Figure 1 [file EMBJ-42-e113418-s005.zip › Main Figure 1/Figure 1G/B7 localization/processed/induced/cropped/cell4 image7_DIC.png]

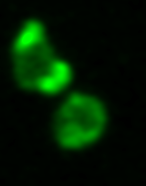

Supplement: Supplementary file 6 — Source Data for Figure 1 [file EMBJ-42-e113418-s005.zip › Main Figure 1/Figure 1G/B7 localization/processed/induced/cropped/cell4 image7_GFP.png]

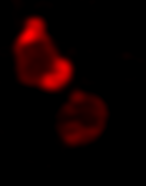

Supplement: Supplementary file 6 — Source Data for Figure 1 [file EMBJ-42-e113418-s005.zip › Main Figure 1/Figure 1G/B7 localization/processed/induced/cropped/cell4 image7_rhodamine.png]

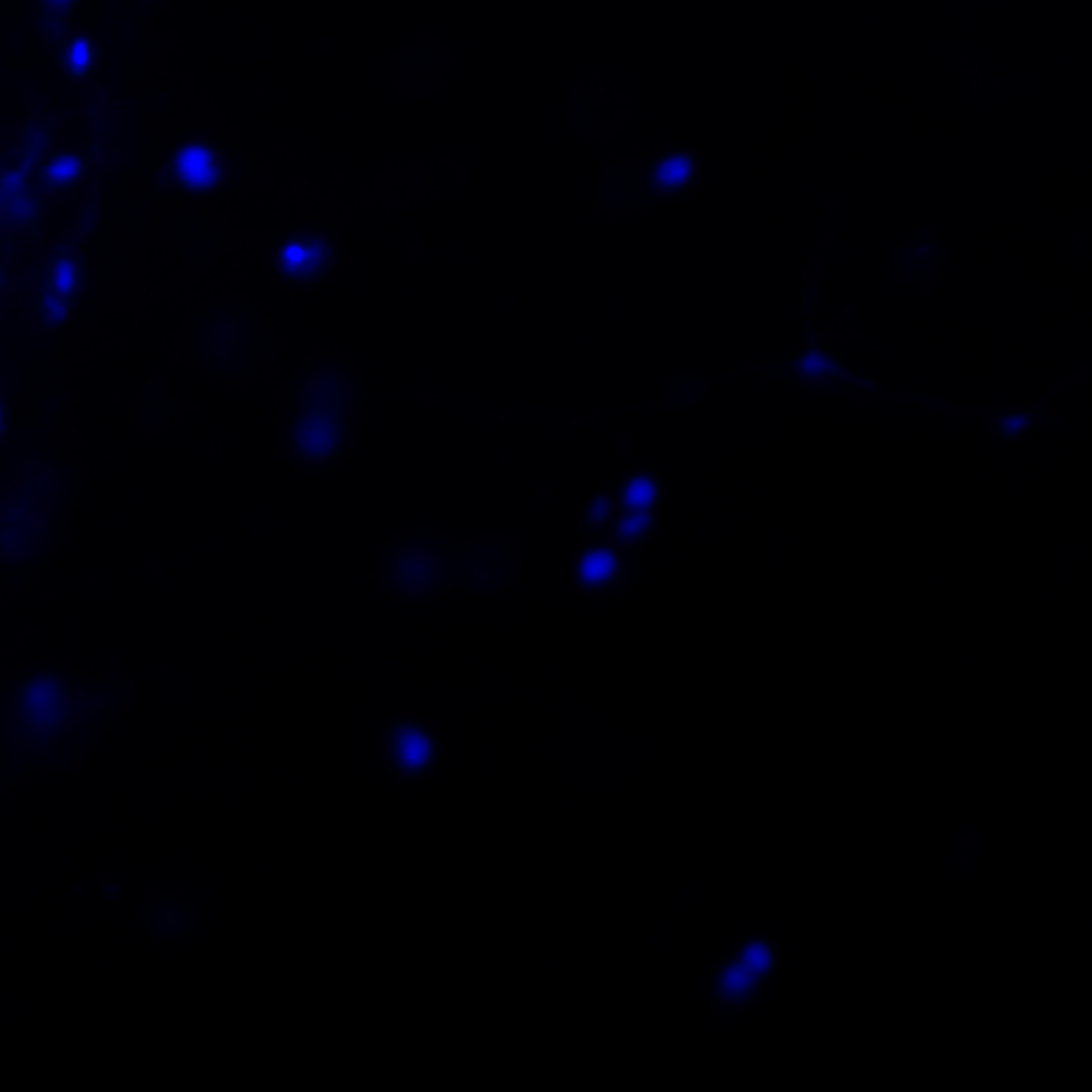

Supplement: Supplementary file 6 — Source Data for Figure 1 [file EMBJ-42-e113418-s005.zip › Main Figure 1/Figure 1G/B7 localization/processed/induced/Image 11_Airyscan Processing_DAPI.tif]

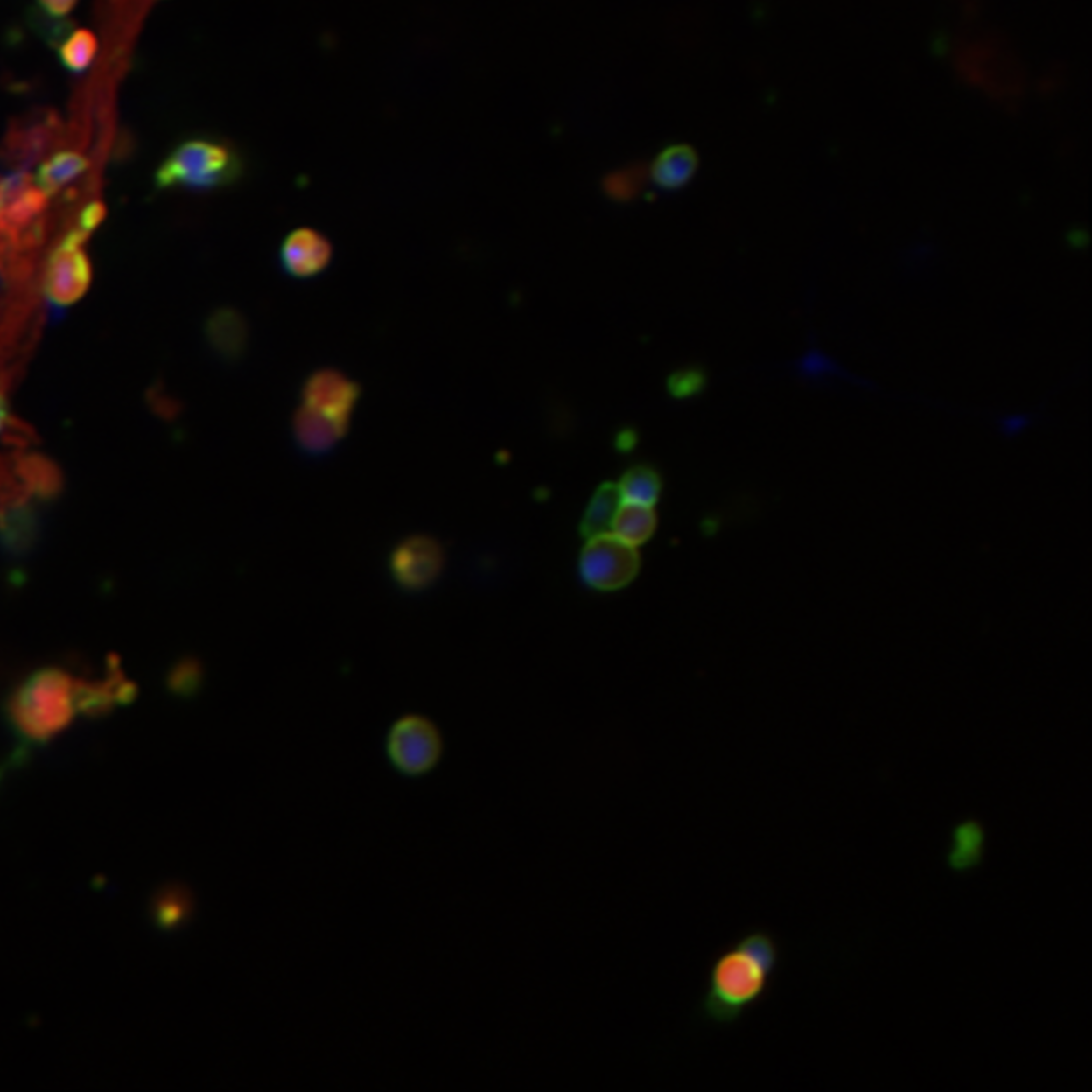

Supplement: Supplementary file 6 — Source Data for Figure 1 [file EMBJ-42-e113418-s005.zip › Main Figure 1/Figure 1G/B7 localization/processed/induced/Image 11_Airyscan Processing_merge.tif]

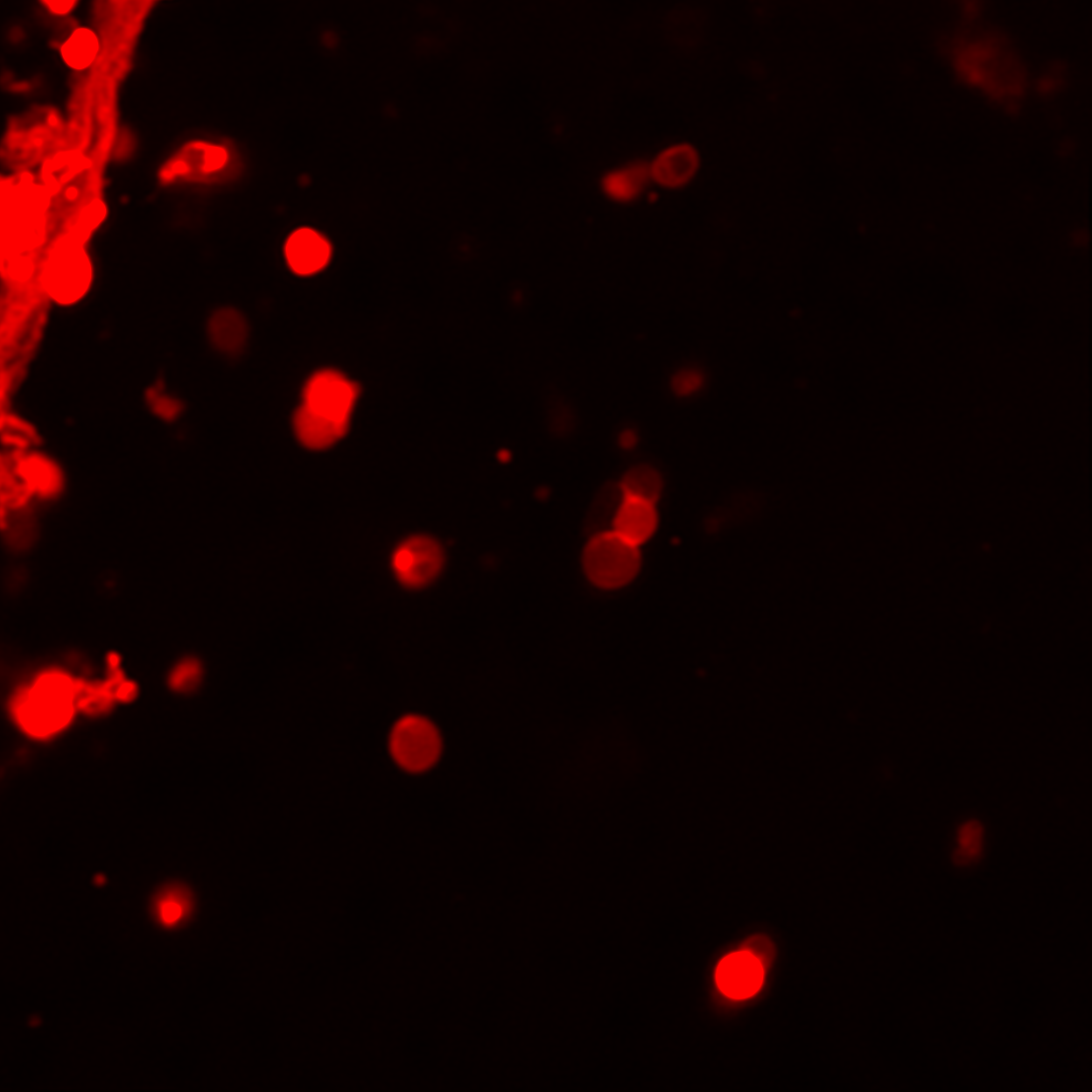

Supplement: Supplementary file 6 — Source Data for Figure 1 [file EMBJ-42-e113418-s005.zip › Main Figure 1/Figure 1G/B7 localization/processed/induced/Image 11_Airyscan Processing_rhodamine.tif]

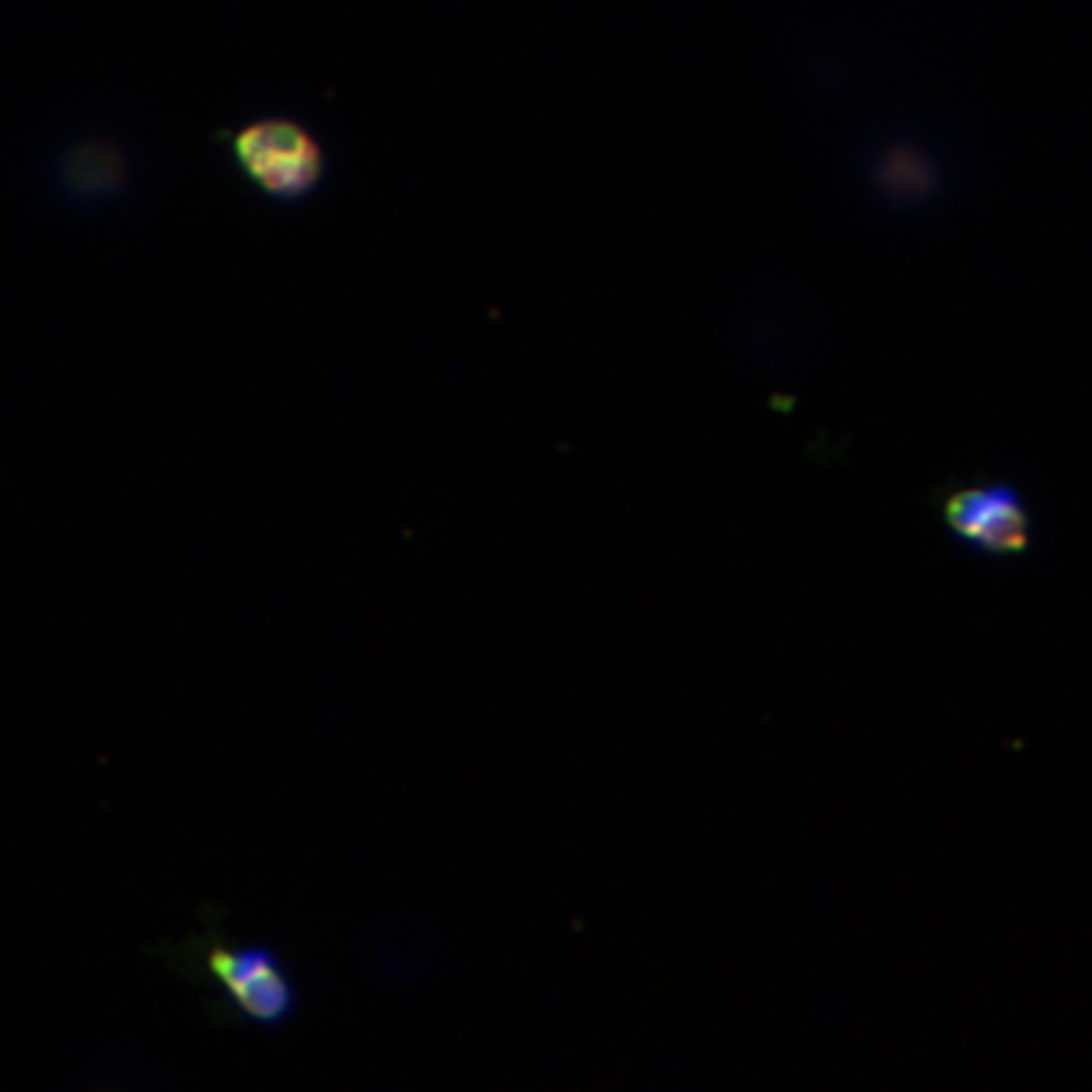

Supplement: Supplementary file 6 — Source Data for Figure 1 [file EMBJ-42-e113418-s005.zip › Main Figure 1/Figure 1G/B7 localization/processed/induced/Image 13_Airyscan Processing.tif]

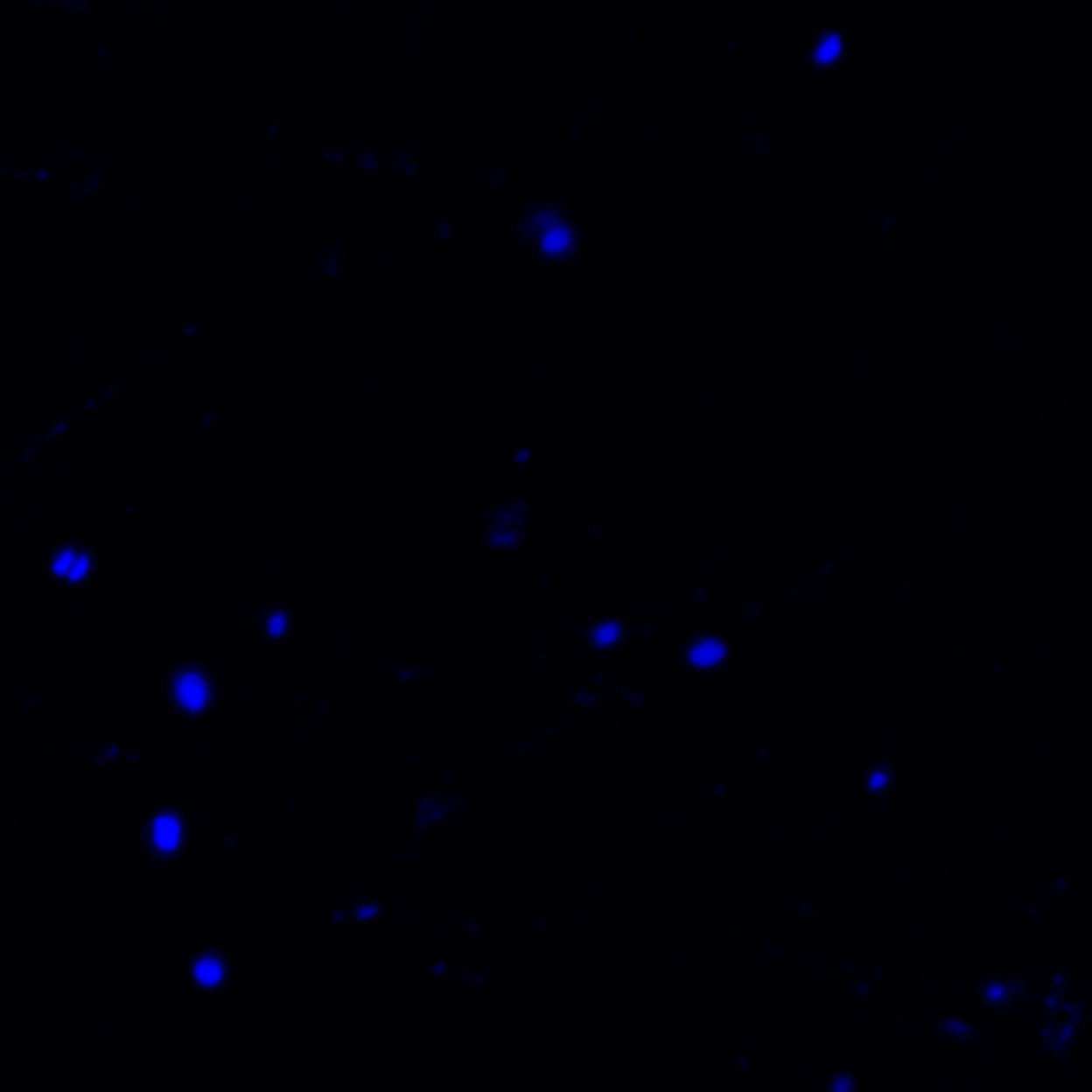

Supplement: Supplementary file 6 — Source Data for Figure 1 [file EMBJ-42-e113418-s005.zip › Main Figure 1/Figure 1G/B7 localization/processed/induced/Image 2_Airyscan Processing_DAPI.tif]

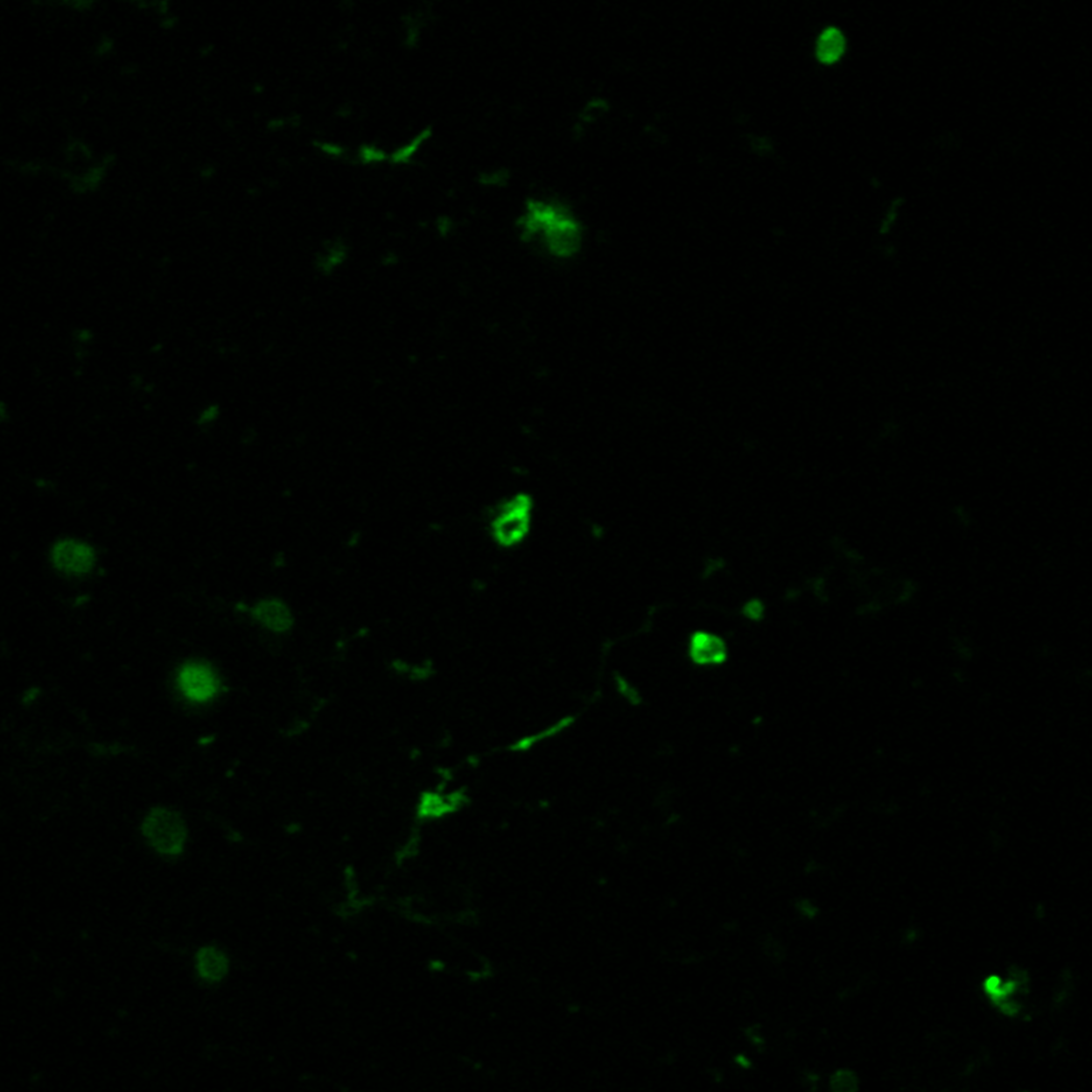

Supplement: Supplementary file 6 — Source Data for Figure 1 [file EMBJ-42-e113418-s005.zip › Main Figure 1/Figure 1G/B7 localization/processed/induced/Image 2_Airyscan Processing_GFP.tif]

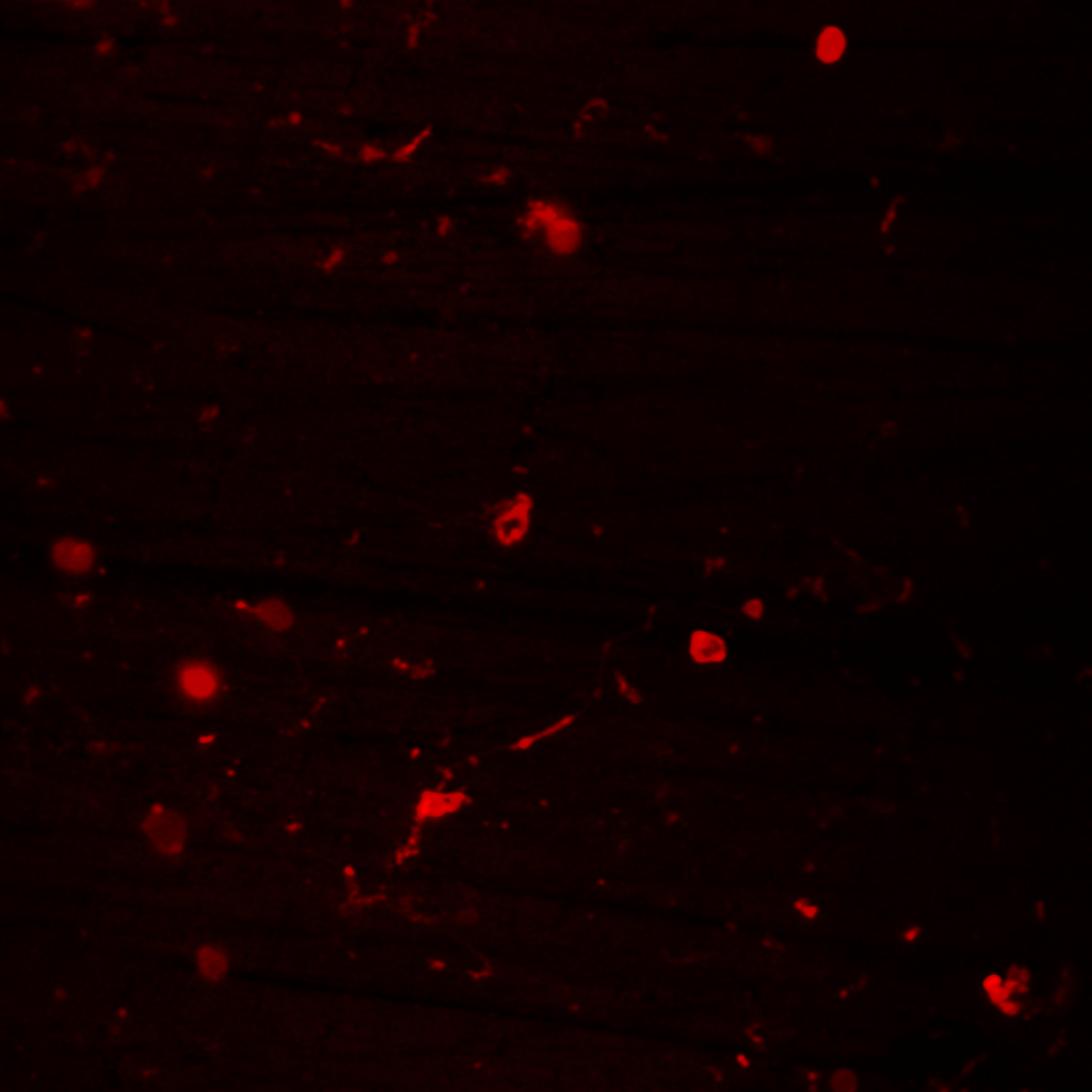

Supplement: Supplementary file 6 — Source Data for Figure 1 [file EMBJ-42-e113418-s005.zip › Main Figure 1/Figure 1G/B7 localization/processed/induced/Image 2_Airyscan Processing_Rhodamine.tif]

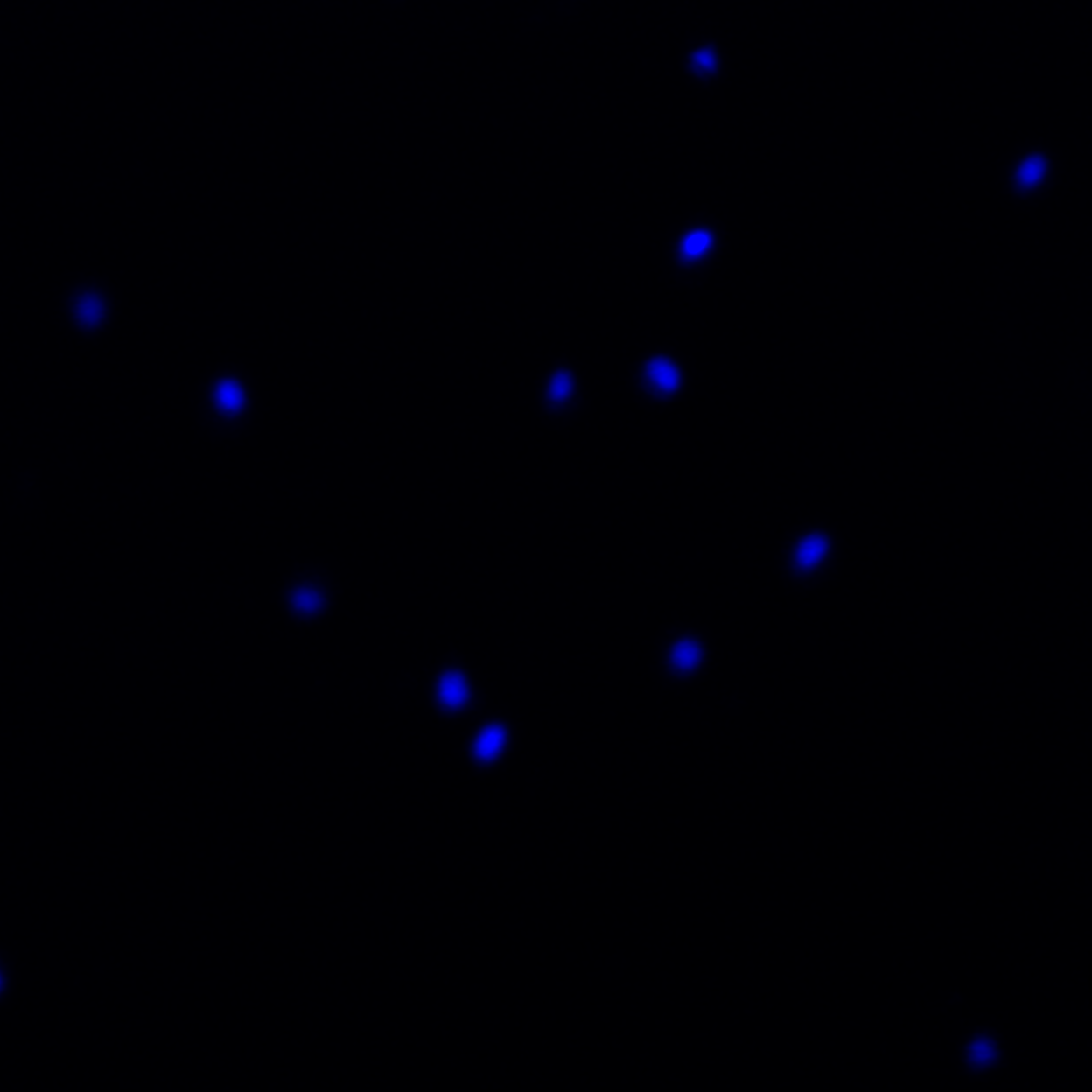

Supplement: Supplementary file 6 — Source Data for Figure 1 [file EMBJ-42-e113418-s005.zip › Main Figure 1/Figure 1G/B7 localization/processed/induced/Image 7_Airyscan Processing_DAPI.tif]

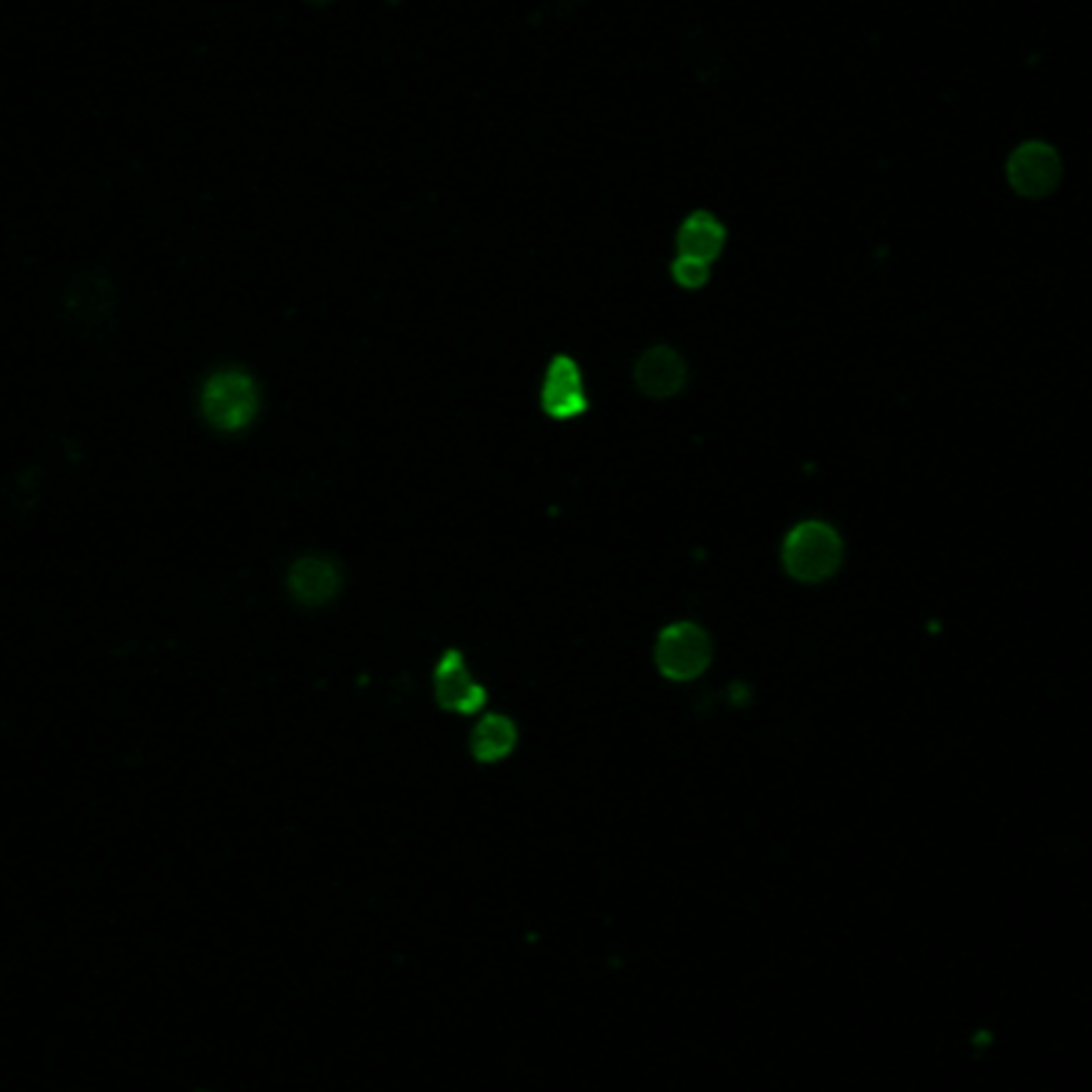

Supplement: Supplementary file 6 — Source Data for Figure 1 [file EMBJ-42-e113418-s005.zip › Main Figure 1/Figure 1G/B7 localization/processed/induced/Image 7_Airyscan Processing_GFP.tif]

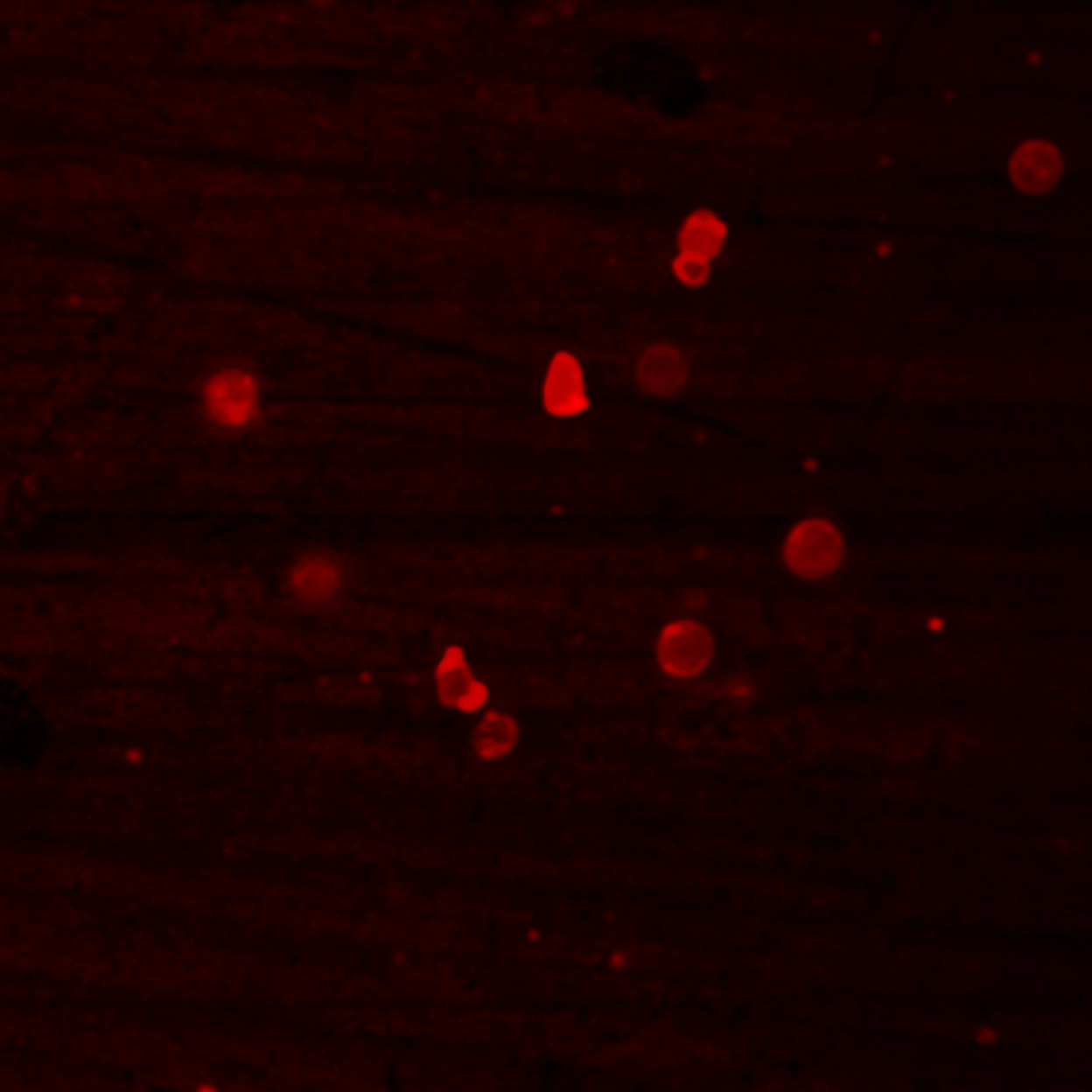

Supplement: Supplementary file 6 — Source Data for Figure 1 [file EMBJ-42-e113418-s005.zip › Main Figure 1/Figure 1G/B7 localization/processed/induced/Image 7_Airyscan Processing_rhodamine.tif]

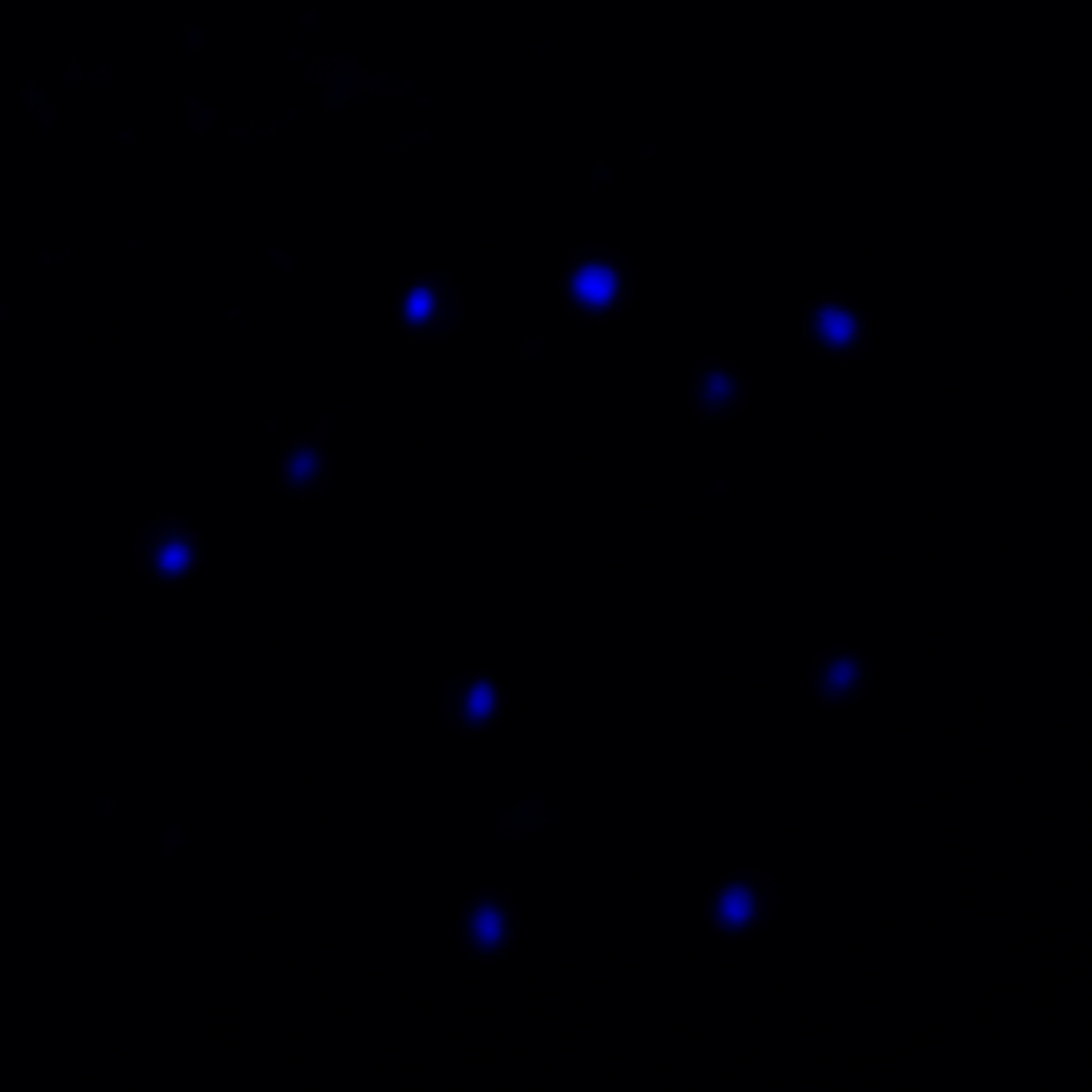

Supplement: Supplementary file 6 — Source Data for Figure 1 [file EMBJ-42-e113418-s005.zip › Main Figure 1/Figure 1G/B7 localization/processed/induced/Image 9_Airyscan Processing_DAPI.tif]

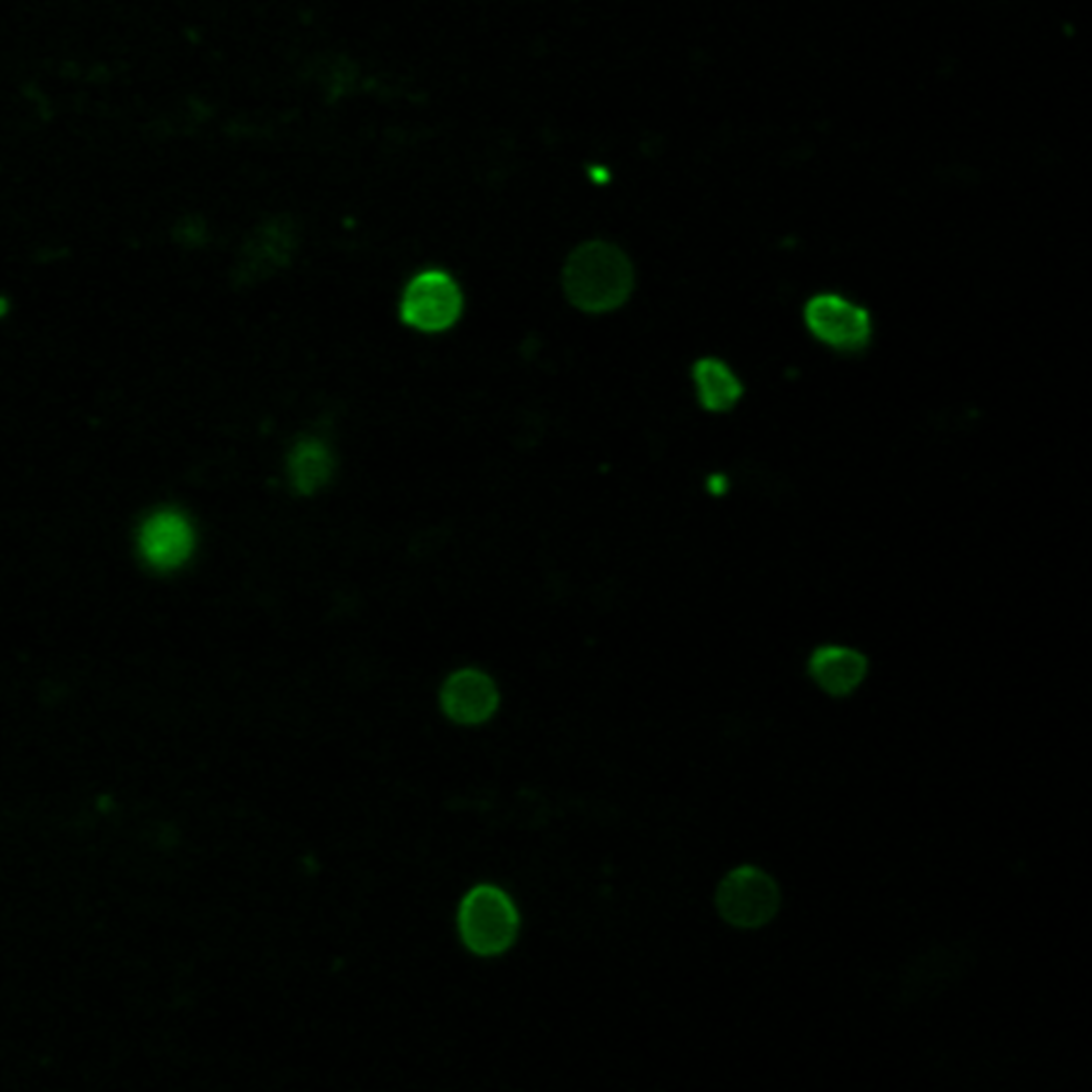

Supplement: Supplementary file 6 — Source Data for Figure 1 [file EMBJ-42-e113418-s005.zip › Main Figure 1/Figure 1G/B7 localization/processed/induced/Image 9_Airyscan Processing_GFP.tif]

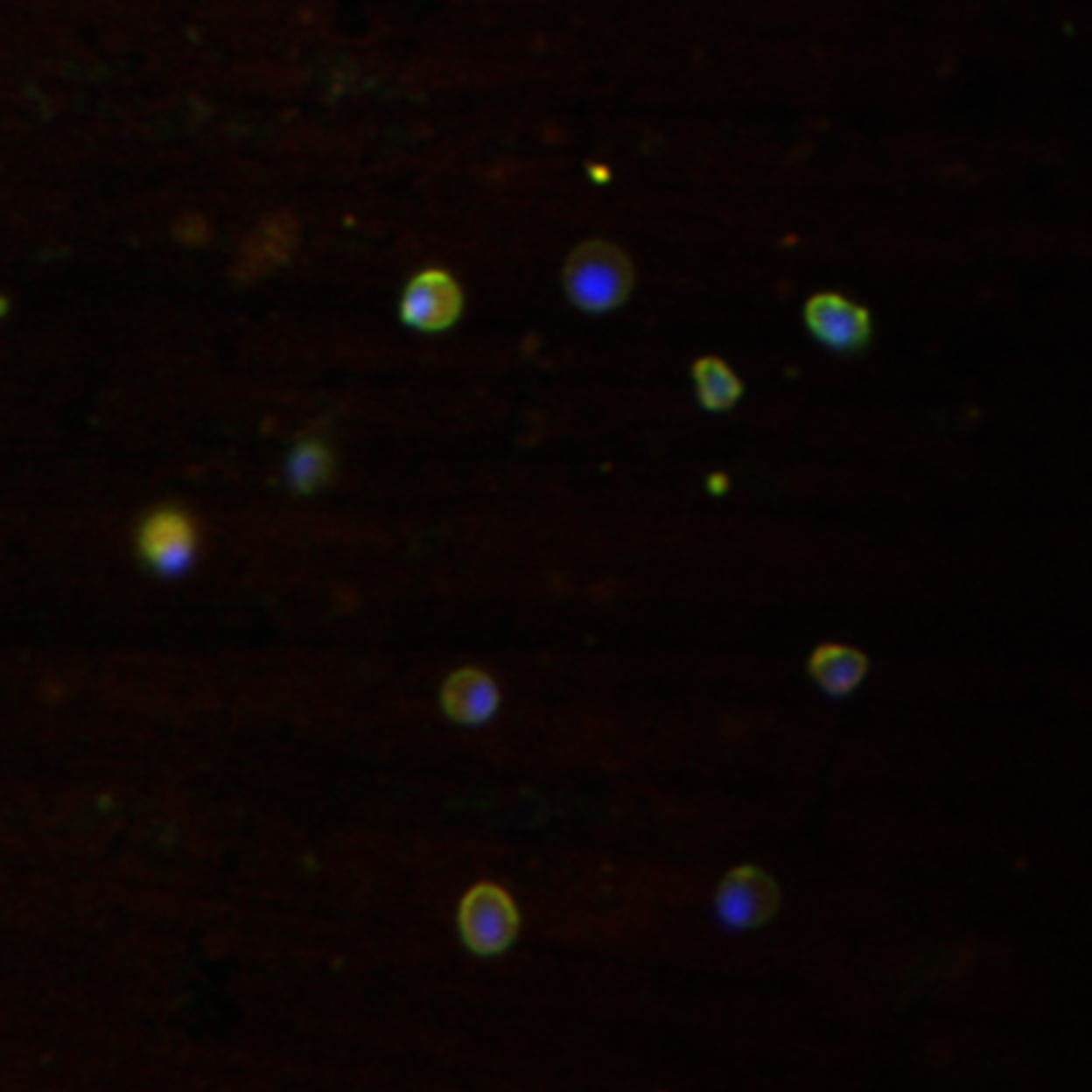

Supplement: Supplementary file 6 — Source Data for Figure 1 [file EMBJ-42-e113418-s005.zip › Main Figure 1/Figure 1G/B7 localization/processed/induced/Image 9_Airyscan Processing_merged.tif]

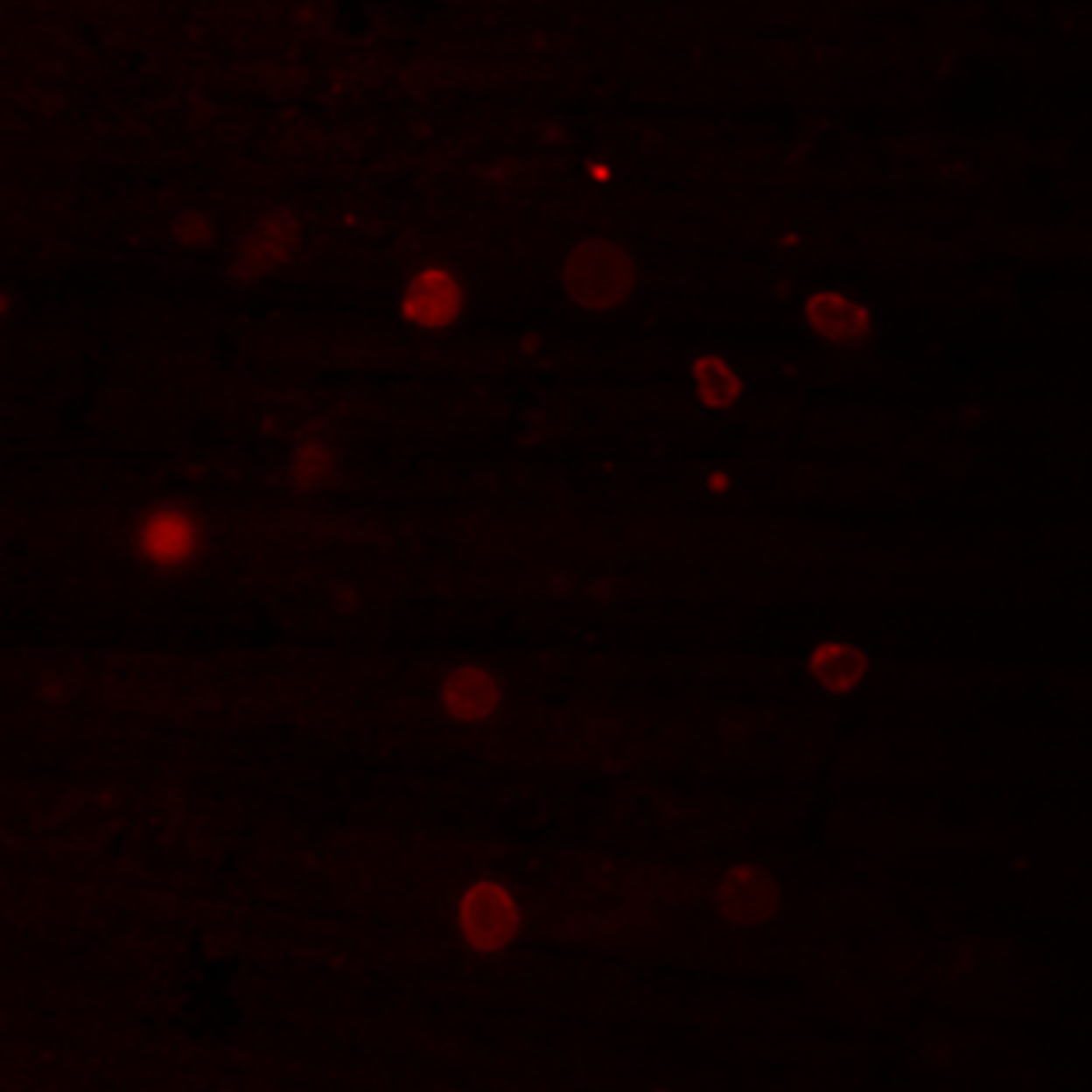

Supplement: Supplementary file 6 — Source Data for Figure 1 [file EMBJ-42-e113418-s005.zip › Main Figure 1/Figure 1G/B7 localization/processed/induced/Image 9_Airyscan Processing_Rhodamine.tif]

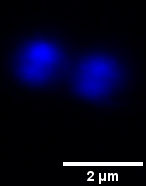

Supplement: Supplementary file 6 — Source Data for Figure 1 [file EMBJ-42-e113418-s005.zip › Main Figure 1/Figure 1G/B7 localization/processed/uninduced/cropped/cell1 Image17_DAPI.png]

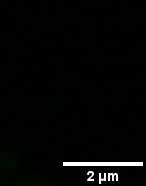

Supplement: Supplementary file 6 — Source Data for Figure 1 [file EMBJ-42-e113418-s005.zip › Main Figure 1/Figure 1G/B7 localization/processed/uninduced/cropped/cell1 Image17_GFP.png]

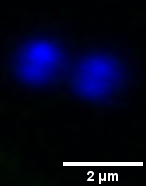

Supplement: Supplementary file 6 — Source Data for Figure 1 [file EMBJ-42-e113418-s005.zip › Main Figure 1/Figure 1G/B7 localization/processed/uninduced/cropped/cell1 Image17_merged.png]

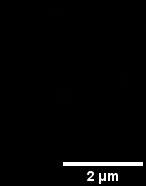

Supplement: Supplementary file 6 — Source Data for Figure 1 [file EMBJ-42-e113418-s005.zip › Main Figure 1/Figure 1G/B7 localization/processed/uninduced/cropped/cell1 Image17_rhodamine.png]

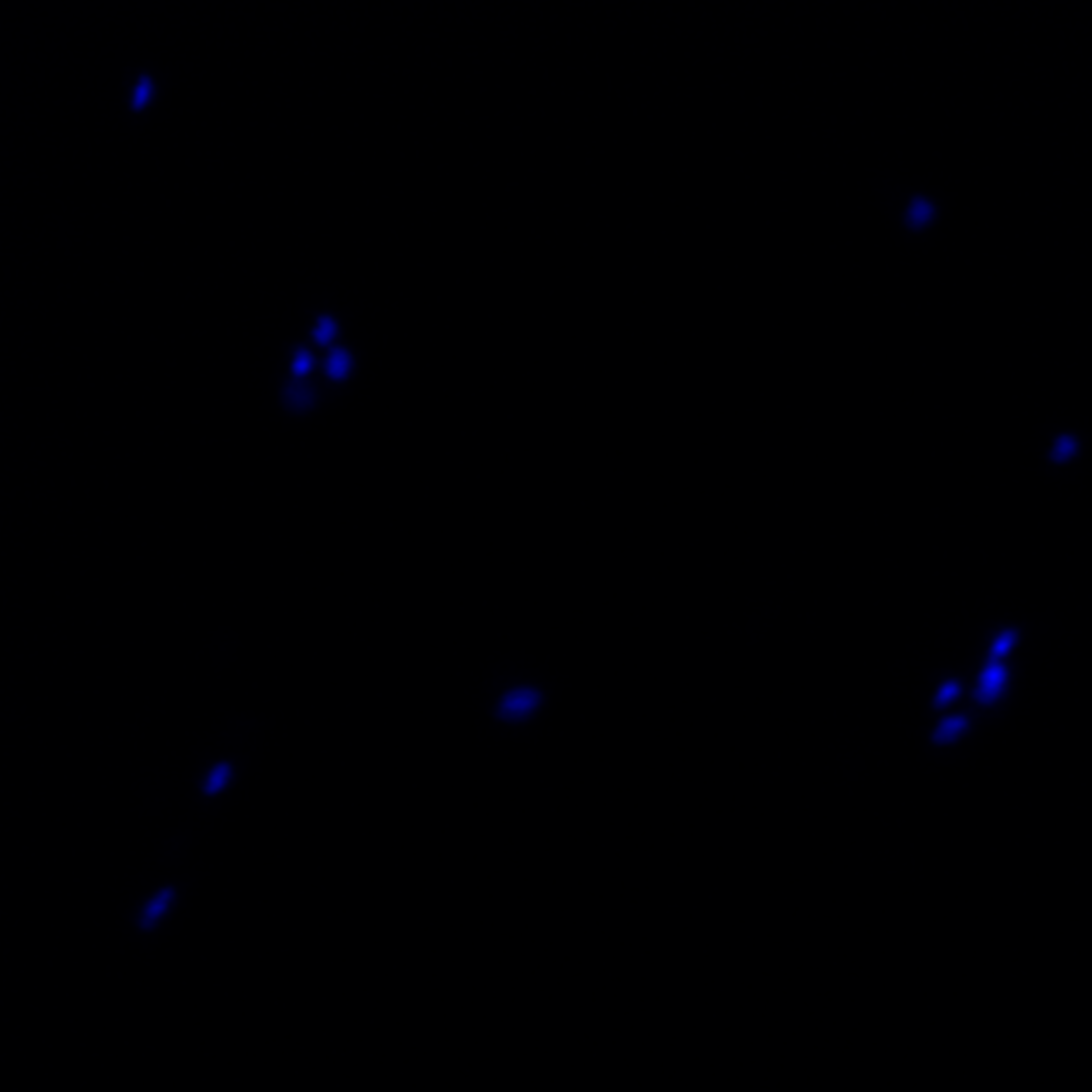

Supplement: Supplementary file 6 — Source Data for Figure 1 [file EMBJ-42-e113418-s005.zip › Main Figure 1/Figure 1G/B7 localization/processed/uninduced/Image 19_Airyscan Processing_DAPI.tif]

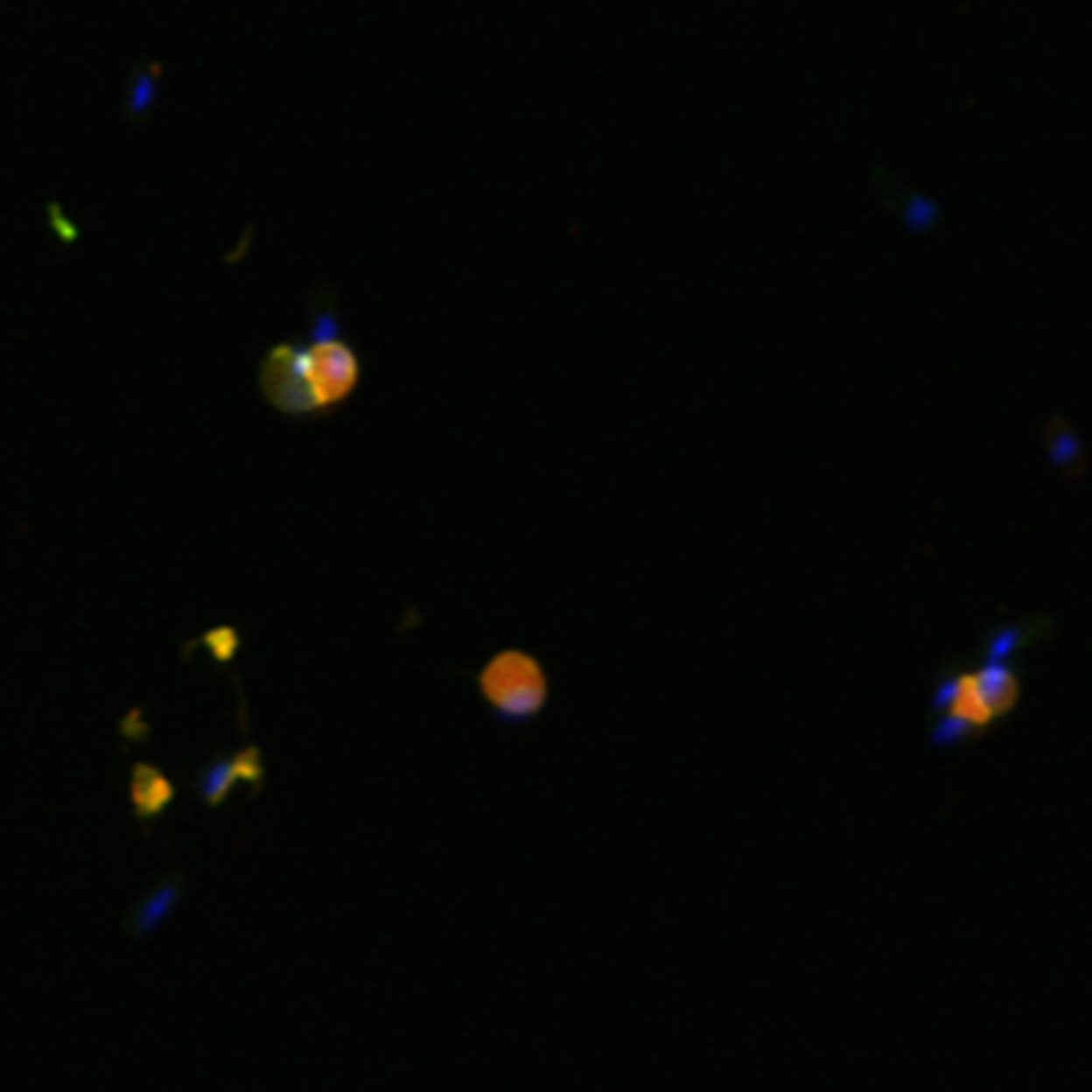

Supplement: Supplementary file 6 — Source Data for Figure 1 [file EMBJ-42-e113418-s005.zip › Main Figure 1/Figure 1G/B7 localization/processed/uninduced/Image 19_Airyscan Processing_merged.tif]

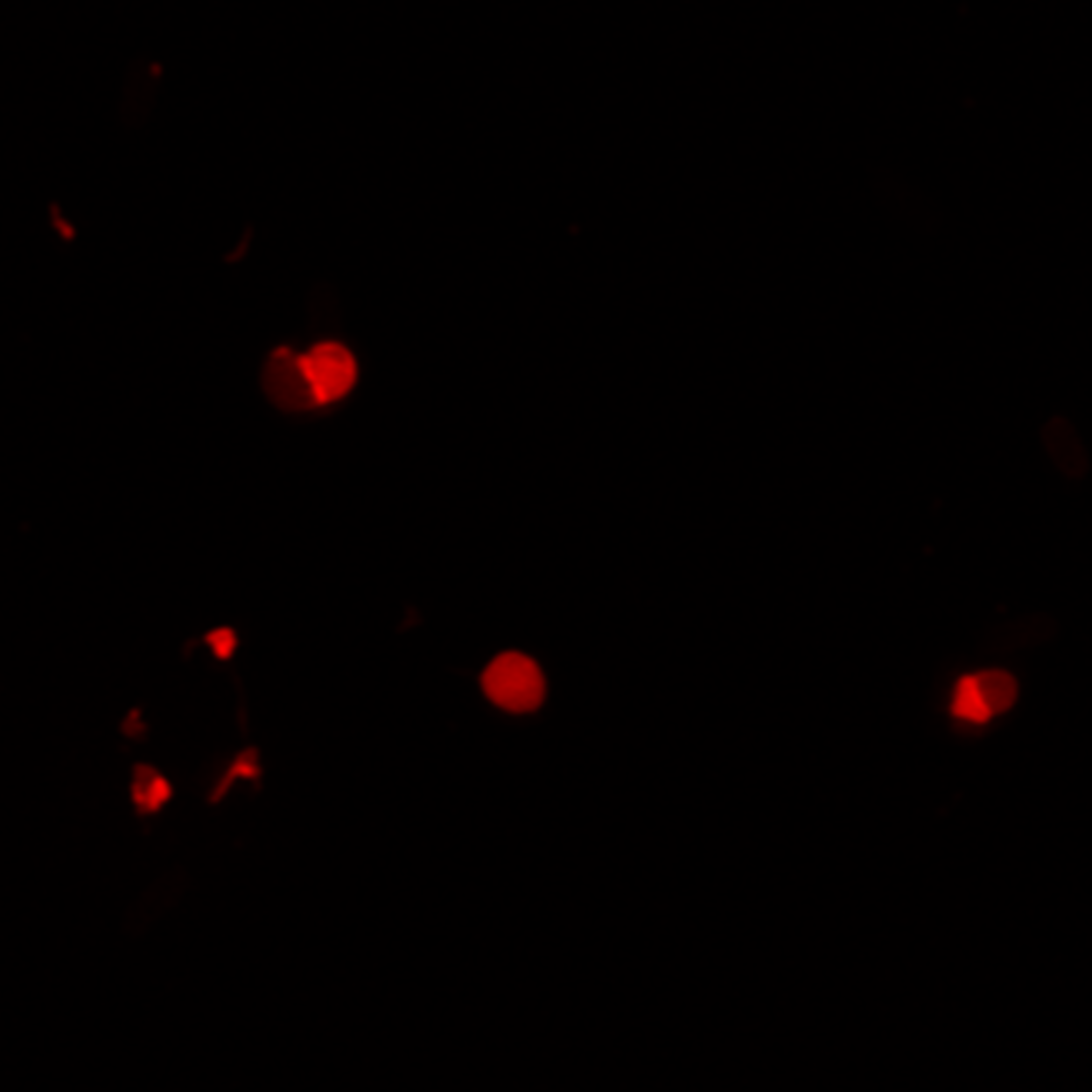

Supplement: Supplementary file 6 — Source Data for Figure 1 [file EMBJ-42-e113418-s005.zip › Main Figure 1/Figure 1G/B7 localization/processed/uninduced/Image 19_Airyscan Processing_Rhodamine.tif]

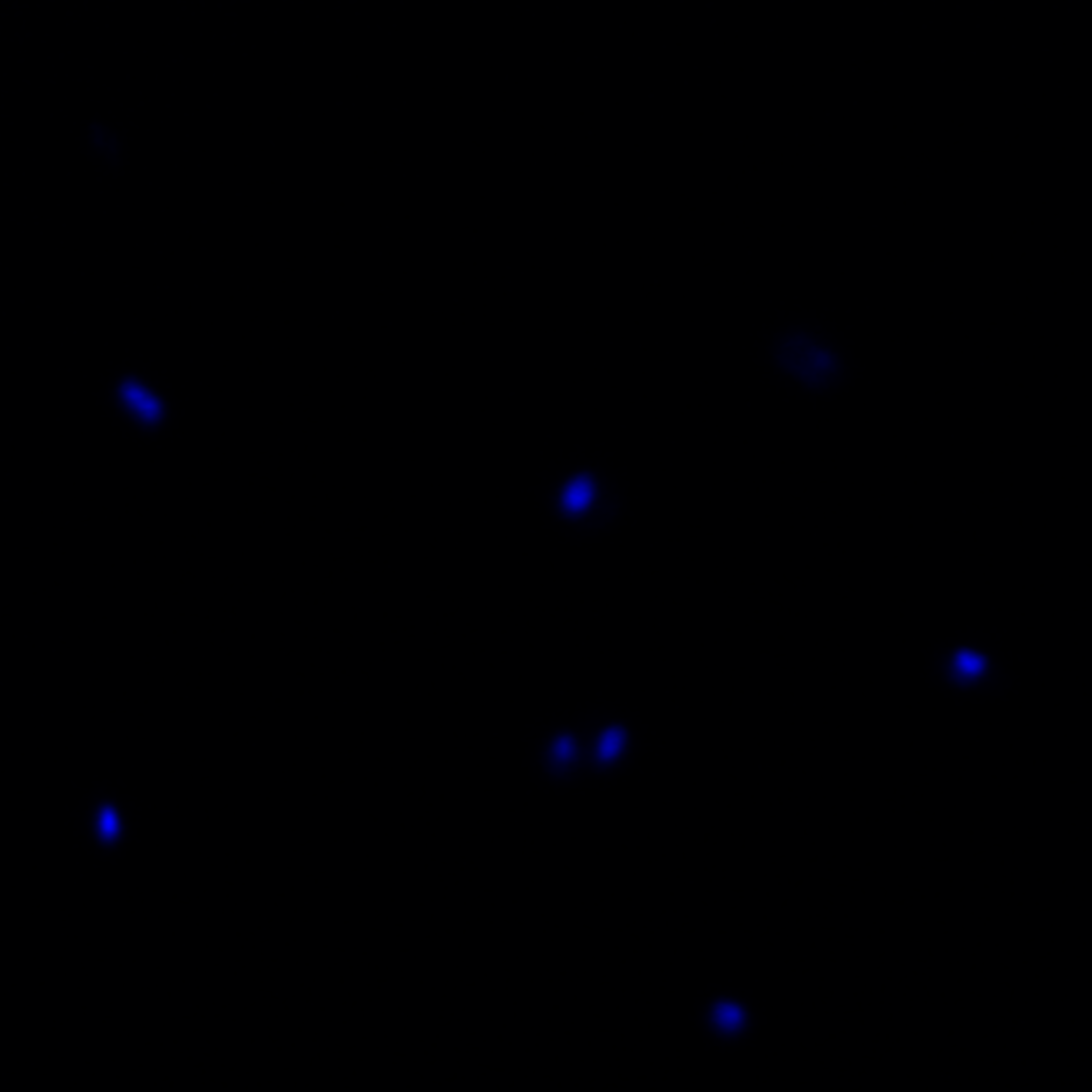

Supplement: Supplementary file 6 — Source Data for Figure 1 [file EMBJ-42-e113418-s005.zip › Main Figure 1/Figure 1G/B7 localization/processed/uninduced/Image 20_Airyscan Processing_DAPI.tif]

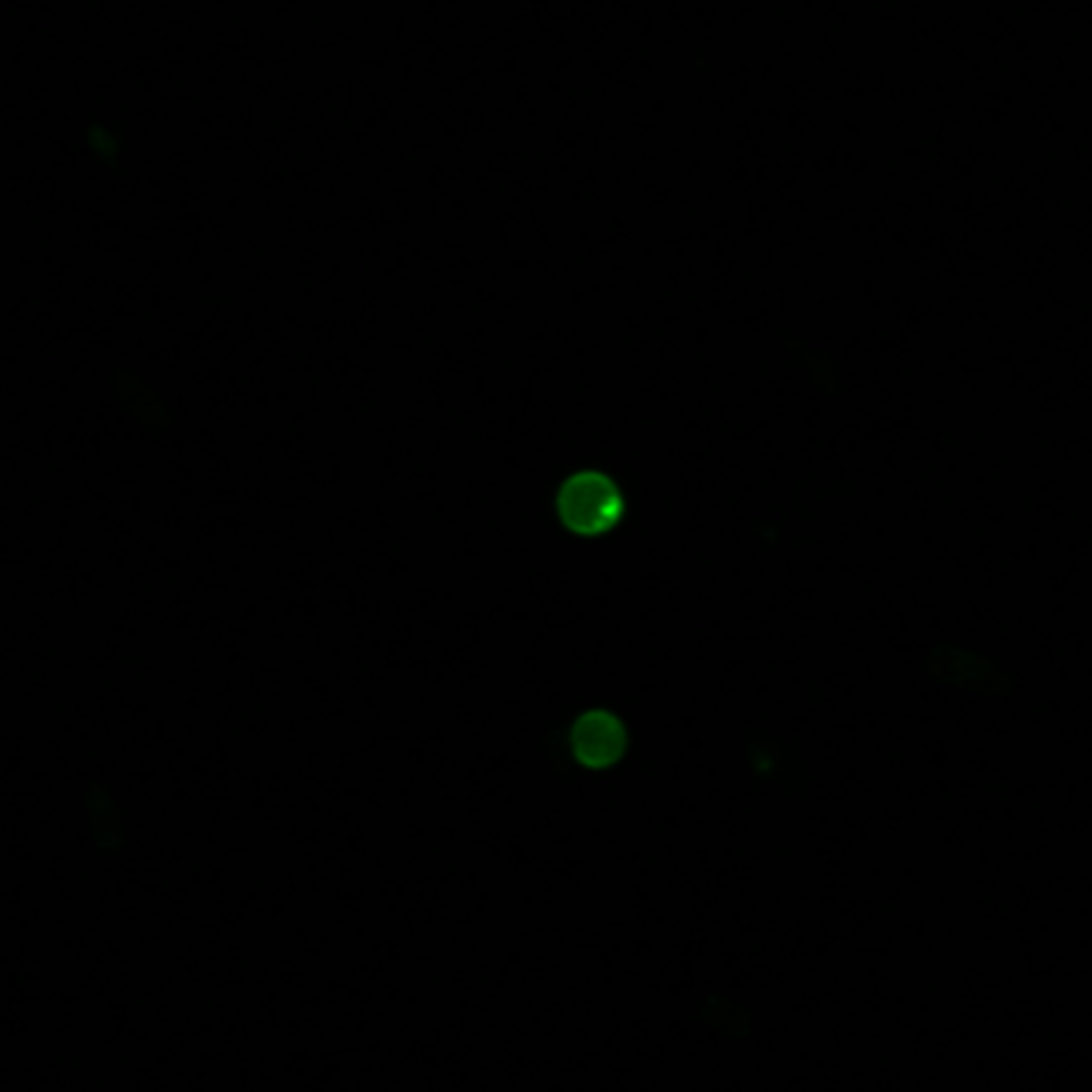

Supplement: Supplementary file 6 — Source Data for Figure 1 [file EMBJ-42-e113418-s005.zip › Main Figure 1/Figure 1G/B7 localization/processed/uninduced/Image 20_Airyscan Processing_GFP.tif]

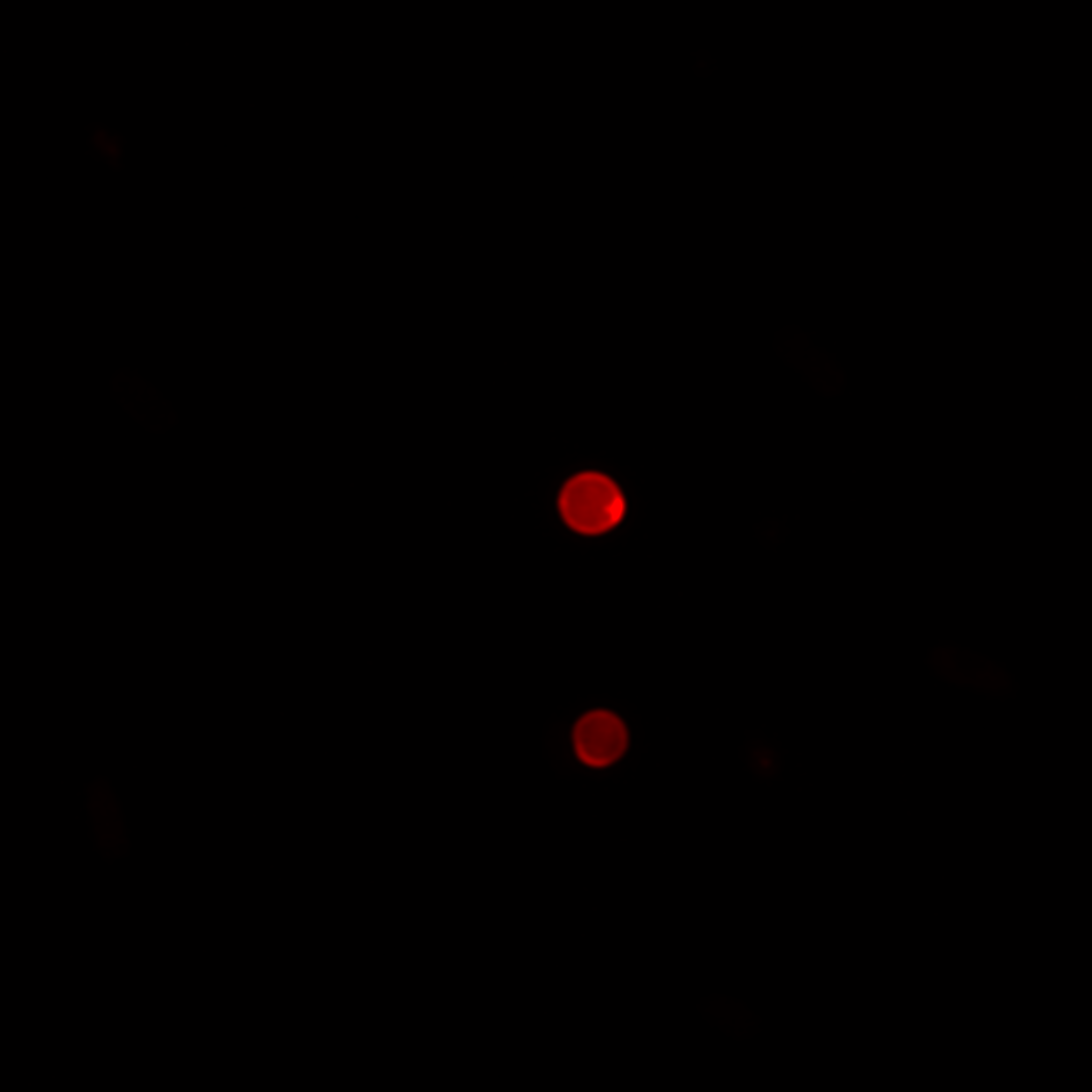

Supplement: Supplementary file 6 — Source Data for Figure 1 [file EMBJ-42-e113418-s005.zip › Main Figure 1/Figure 1G/B7 localization/processed/uninduced/Image 20_Airyscan Processing_Rhodamine.tif]

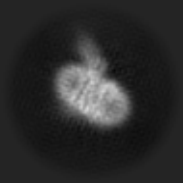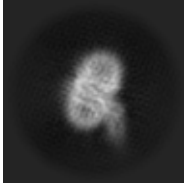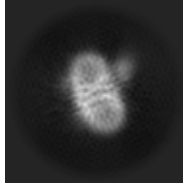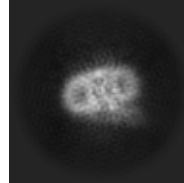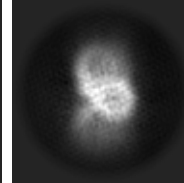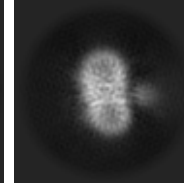

Supplement: Supplementary file 6 — Source Data for Figure 1 [file EMBJ-42-e113418-s005.zip › Main Figure 1/Figure 1H/P11_J58_selected_6_classes.pdf]

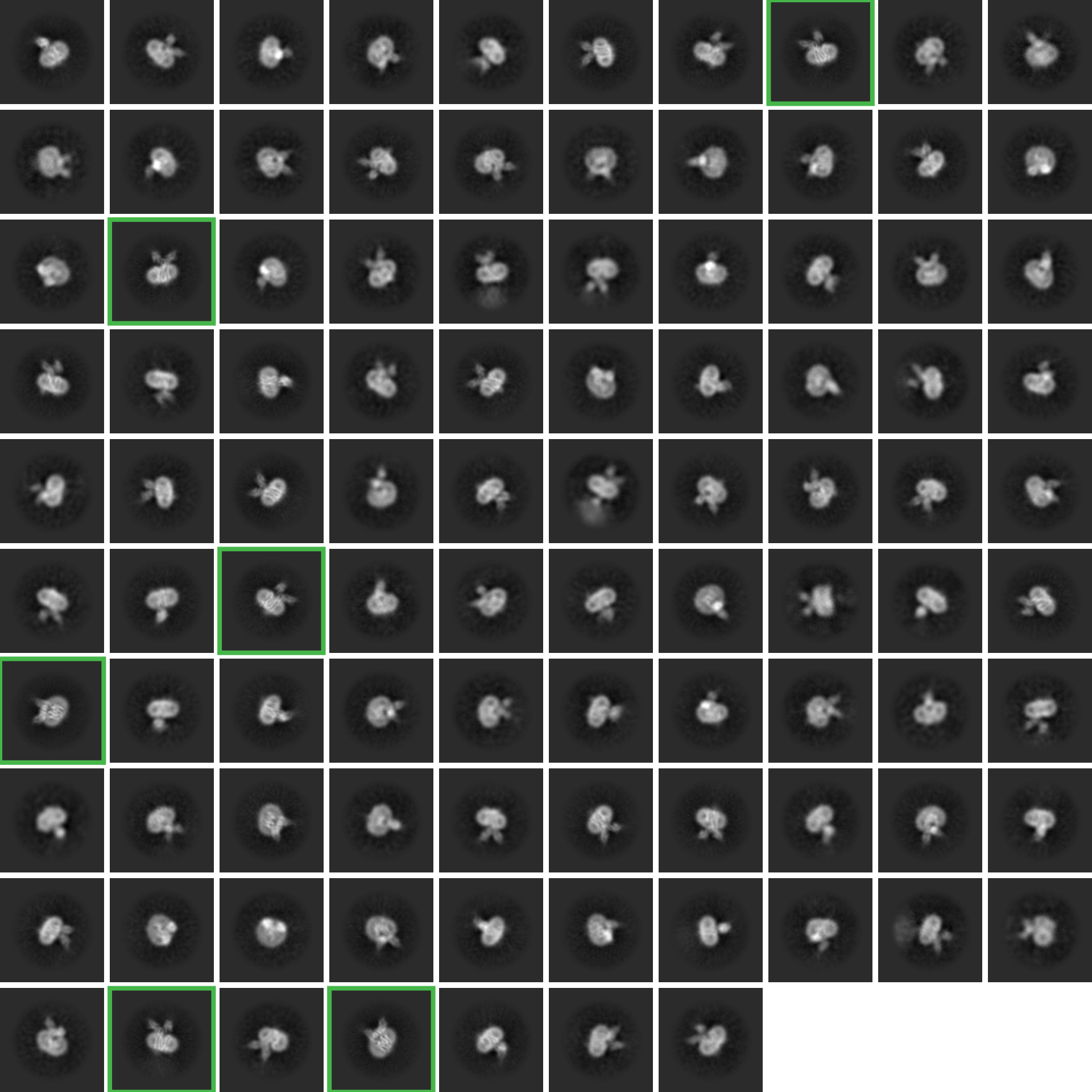

Supplement: Supplementary file 6 — Source Data for Figure 1 [file EMBJ-42-e113418-s005.zip › Main Figure 1/Figure 1H/P16_J24_selected_97_classes 6 boxed.pdf]

**Figure 4D spot assays**

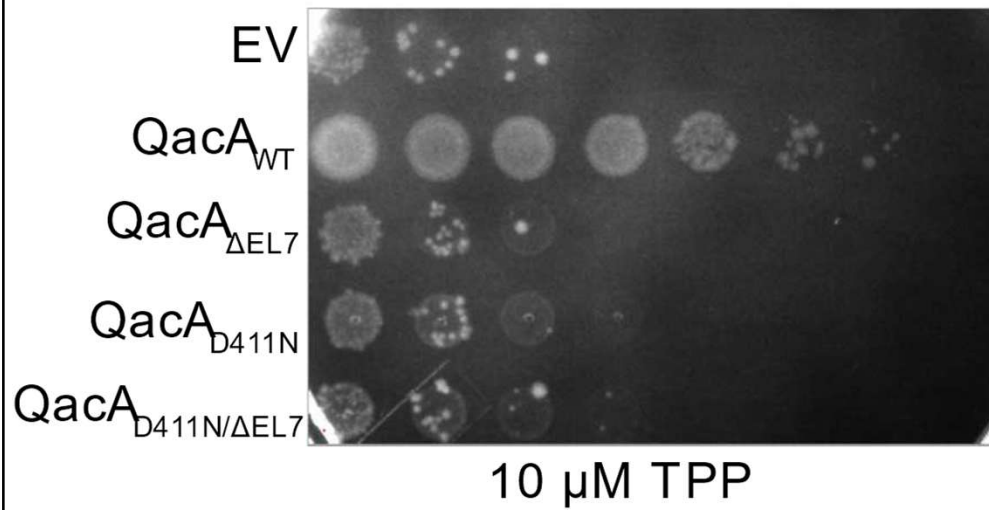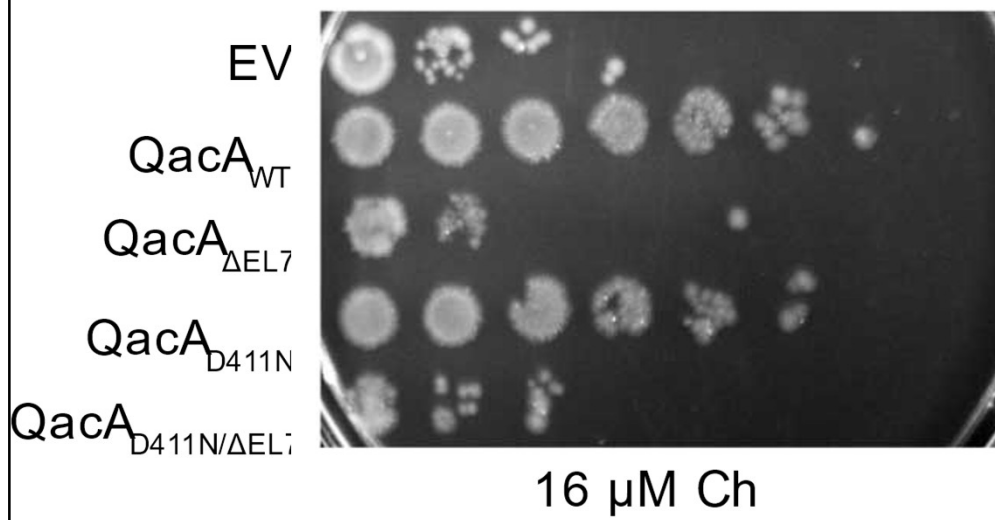

Supplement: Supplementary file 7 — Source Data for Figure 4 [file EMBJ-42-e113418-s006.zip › Main Figure 4/Figure 4D spot assay.pdf]

**Figure 5D spot assays**

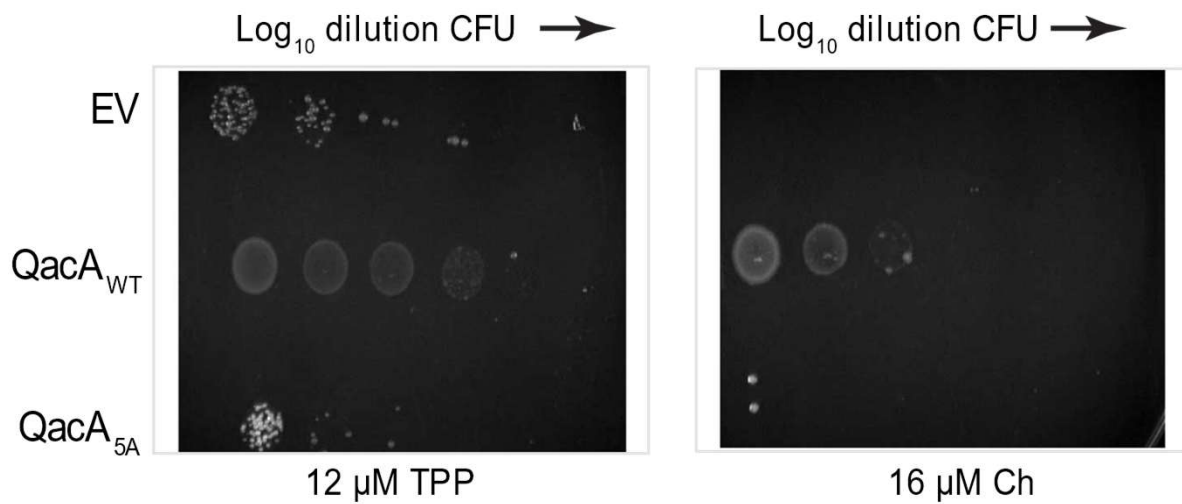

**Figure 5G spot assays**

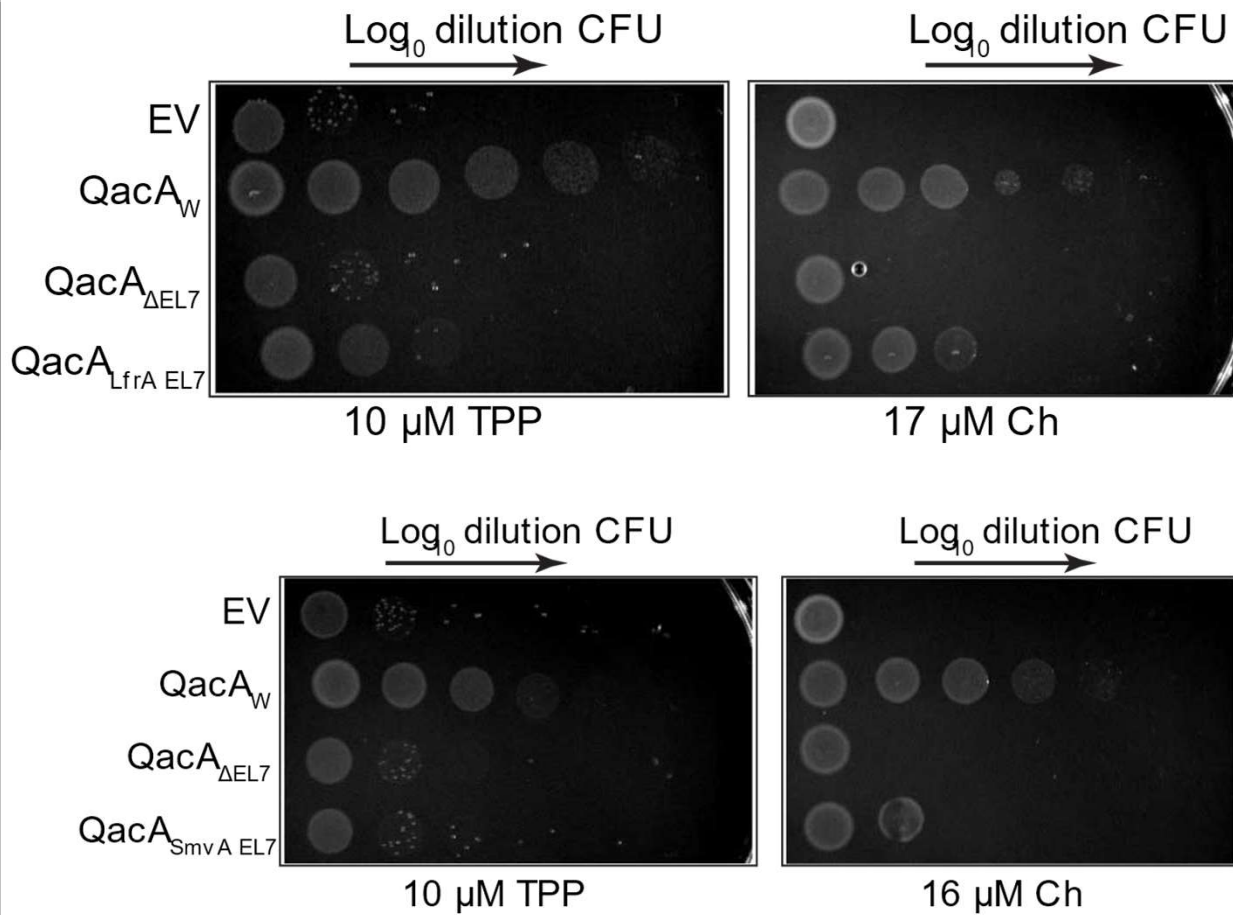

Supplement: Supplementary file 8 — Source Data for Figure 5 [file EMBJ-42-e113418-s001.zip › Main Figure 5/Figure 5D and G/Figrue 5D and G spot assay.pdf]
