# Supplementary material for: Cryo‐EM structure of antibacterial efflux transporter QacA from Staphylococcus aureus reveals a novel extracellular loop with allosteric role
Source: EMBO J. 2023 Jul 17;42(16):e113418. doi: 10.15252/embj.2023113418 (PMC10425836; doi:10.15252/embj.2023113418)
Supplement: Supplementary file 9 [file EMBJ-42-e113418-s004.pdf]

THE EMBO JOURNAL

Cryo-EM structure of antibacterial efflux transporter QacA in Staphylococcus aureus reveals a novel extracellular loop with allosteric role

Puja Majumder, Shahbaz Ahmad, Pragya Ahuja, Arunabh Athreya, Rakesh Ranjan, and Aravind Penmatsa
DOI: 10.15252/embj.2023113418

Corresponding author(s): Aravind Penmatsa (penmatsa@iisc.ac.in)

Review Timeline:

Submission Date:	1st Jan 23
Editorial Decision:	23rd Jan 23
Revision Received:	7th May 23
Editorial Decision:	9th Jun 23
Revision Received:	21st Jun 23
Accepted:	26th Jun 23

Editor: Daniel Klimmeck

Transaction Report:

(Note: With the exception of the correction of typographical or spelling errors that could be a source of ambiguity, letters and reports are not edited. Depending on transfer agreements, referee reports obtained elsewhere may or may not be included in this compilation. Referee reports are anonymous unless the Referee chooses to sign their reports.)

1st Editorial Decision **23rd Jan 2023**

Dear Dr Aravind Penmatsa,

Thank you for the submission of your manuscript (EMBOJ-2022-113418) to The EMBO Journal, as well as for your patience with our response at this time of the year. Your study has been sent to three reviewers with transporter structural and functional expertise and we have received feedback from all of them, which I enclose below.

As you will see, the referees acknowledge the potential interest and novelty of your results, although they also express several issues that will have to be conclusively addressed before they can be supportive of publication of your manuscript in The EMBO Journal. In more detail, the reviewers raise a number of points related to additional controls required, improved methods annotation and data processing, overall discussion and acknowledgement of the limitations of the findings as well as textual comments and typos, that would need to be conclusively addressed to achieve the level of robustness and clarity needed for The EMBO Journal.

I judge the comments of the referees to be generally reasonable and given their overall interest, we are in principle happy to invite you to revise your manuscript experimentally to address the referees' comments.

As you may remember from previous experience, we generally allow three months as standard revision time. As a matter of policy, competing manuscripts published during this period will not negatively impact on our assessment of the conceptual advance presented by your study. However, we request that you contact the editor as soon as possible upon publication of any related work, to discuss how to proceed. Should you foresee a problem in meeting this three-month deadline, please let us know in advance and we may be able to grant an extension.

When submitting your revised manuscript, please carefully review the instructions below.

Thank you for the opportunity to consider your work for publication.
I look forward to your revision.

Best regards,

Daniel Klimmeck

Daniel Klimmeck, PhD
Senior Editor
The EMBO Journal

Instruction for the preparation of your revised manuscript:

1) a .docx formatted version of the manuscript text (including legends for main figures, EV figures and tables). Please make sure that the changes are highlighted to be clearly visible.

2) individual production quality figure files as .eps, .tif, .jpg (one file per figure).

3) a .docx formatted letter INCLUDING the reviewers' reports and your detailed point-by-point response to their comments. As part of the EMBO Press transparent editorial process, the point-by-point response is part of the Review Process File (RPF), which will be published alongside your paper.

4) a complete author checklist, which you can download from our author guidelines (https://wol-prod-cdn.literatumonline.com/pb-assets/embo-site/Author Checklist%20-%20EMBO%20J-1561436015657.xlsx). Please insert information in the checklist that is also reflected in the manuscript. The completed author checklist will also be part of the RPF.

5) Please note that all corresponding authors are required to supply an ORCID ID for their name upon submission of a revised manuscript.

6) It is mandatory to include a 'Data Availability' section after the Materials and Methods. Before submitting your revision, primary datasets produced in this study need to be deposited in an appropriate public database, and the accession numbers and database listed under 'Data Availability'. Please remember to provide a reviewer password if the datasets are not yet public (see https://www.embopress.org/page/journal/14602075/authorguide#datadeposition).
In case you have no data that requires deposition in a public database, please state so in this section. Note that the Data Availability Section is restricted to new primary data that are part of this study.
*** Note - All links should resolve to a page where the data can be accessed. ***

7) Our journal encourages inclusion of *data citations in the reference list* to directly cite datasets that were re-used and obtained from public databases. Data citations in the article text are distinct from normal bibliographical citations and should directly link to the database records from which the data can be accessed. In the main text, data citations are formatted as follows: "Data ref: Smith et al, 2001" or "Data ref: NCBI Sequence Read Archive PRJNA342805, 2017". In the Reference list, data citations must be labeled with "[DATASET]". A data reference must provide the database name, accession number/identifiers and a resolvable link to the landing page from which the data can be accessed at the end of the reference. Further instructions are available at .

8) At EMBO Press we ask authors to provide source data for the main and EV figures. Our source data coordinator will contact you to discuss which figure panels we would need source data for and will also provide you with helpful tips on how to upload and organize the files.

Numerical data can be provided as individual .xls or .csv files (including a tab describing the data). For 'blots' or microscopy, uncropped images should be submitted (using a zip archive or a single pdf per main figure if multiple images need to be supplied for one panel). Additional information on source data and instruction on how to label the files are available at .

9) We replaced Supplementary Information with Expanded View (EV) Figures and Tables that are collapsible/expandable online (see examples in https://www.embopress.org/doi/10.15252/embj.201695874). A maximum of 5 EV Figures can be typeset. EV Figures should be cited as 'Figure EV1, Figure EV2" etc. in the text and their respective legends should be included in the main text after the legends of regular figures.

- For the figures that you do NOT wish to display as Expanded View figures, they should be bundled together with their legends in a single PDF file called *Appendix*, which should start with a short Table of Content. Appendix figures should be referred to in the main text as: "Appendix Figure S1, Appendix Figure S2" etc. See detailed instructions regarding expanded view here: .

- Additional Tables/Datasets should be labelled and referred to as Table EV1, Dataset EV1, etc. Legends have to be provided in a separate tab in case of .xls files. Alternatively, the legend can be supplied as a separate text file (README) and zipped together with the Table/Dataset file.

10) When assembling figures, please refer to our figure preparation guideline in order to ensure proper formatting and readability in print as well as on screen:
http://bit.ly/EMBOPressFigurePreparationGuideline

Please remember: Digital image enhancement is acceptable practice, as long as it accurately represents the original data and conforms to community standards. If a figure has been subjected to significant electronic manipulation, this must be noted in the figure legend or in the 'Materials and Methods' section. The editors reserve the right to request original versions of figures and the original images that were used to assemble the figure.

11) For data quantification: please specify the name of the statistical test used to generate error bars and P values, the number (n) of independent experiments (specify technical or biological replicates) underlying each data point and the test used to calculate p-values in each figure legend. The figure legends should contain a basic description of n, P and the test applied. Graphs must include a description of the bars and the error bars (s.d., s.e.m.).

Further information is available in our Guide to Authors: https://www.embopress.org/page/journal/14602075/authorguide

We realize that it is difficult to revise to a specific deadline. In the interest of protecting the conceptual advance provided by the work, we recommend a revision within 3 months (23rd Apr 2023). Please discuss the revision progress ahead of this time with the editor if you require more time to complete the revisions. Use the link below to submit your revision:

https://emboj.msubmit.net/cgi-bin/main.plex

Referee #1:

This manuscript by Majumder et al. reports the cryoEM structure of QacA, which mediates proton-coupled efflux of mono and divalent cationic antibacterial compounds in Staphylococcus aureus. The authors inserted a single mutation D411N to improve homogeneity - without abolishing dequalinium and chlorhexidine efflux activity - and resolved the structure of substrate-free QacA in an outward open conformation to a resolution of 3.6 Å. QacA exhibits an extracellular helical hairpin loop between TMs

13 and 14, which is conserved in a subset of drug:proton antiport (DHA) transporters. They demonstrate that removal of the EL7 hairpin loop compromises efflux activity and that chimeras between QacA and LfrA exhibit reduced efflux activity. They conclude that EL7 hairpin allosterically regulates efflux in QacA.
This is an interesting manuscript that reports the combination of a large variety of methods to understand a biologically very important transporter. My main concerns are the sparseness in the methods description and the overall lack of a clear conclusion of this work. Is it really necessary to combine cryo-EM, MD simulation, recombinant DNA technology, functional data to conclude that an external hairpin fulfills an allosteric and crucial role for function? I would expect a clear mechanistic conclusion in such work..
1. P. 7, Fig. 3, The authors use APBS to determine electrostatic potentials. Please describe the procedures in the Method section. Moreover, please discuss the limitations of APBS.
2. The reported electrotstatic potentials are significantly more negative than in related transporters. Please discuss potential consequences of this feature! Could it be that non-subtrate cations, such as Na+ and K+, accumulated in the vestibule? Are these transporters Na+ or K+ dependent? Are there functional data in the presence and in the absence of small cations? Are the positive electrostic potential a hallmark of drug:proton antiporters in the MFS family?
3. P. 10, Fig. 5. The authors performed equilibrium MD simulations to elucidate a transition from the outward-open QacA structure to the inward-facing occluded state. Obviously, the transporter returns in the apo state from the outward into the inward facing conformation. This should not be possible when the proton-acceptor - necessary for drug-proton antiport is protonated. Did the authors try this?
4. How was this simulation exactly done? Is there any substrate present? How often such simulations were performed? Are there any statistics? What is the conclusion?
5. How was the AlphaFold2 prediction of the inward-facing conformation performed?
6. The authors demonstrate that deletion of EL7 abolishes efflux transport and that substitution of EL7 from related transporters partially restores activity. This seems to be a major result. However, I could not find any no mechanistic role of EL7 in this function. Given the high experimental and theoretical efforts of the authors such conclusion is required.
7. p.18: "All the acidic residues that were solvent exposed were kept deprotonated except D411, which was modelled with a neutral side chain." Why was D411 simulated with neutral side chain? To account for the mutation in the expression construct? Please discuss in this context how these transporters are thought to mediate drug-proton antiport?

Referee #2:

The manuscript by Majumder et al. describes the structural characterization of a proton coupled drug efflux antiporter of the MFS superfamily with a critical role for antimicrobial resistance of the pathogenic bacterium Staphylococcus aureus. The study presents insights from a 3.6 Å nanobody-complexed cryo-EM structure of the transporter QacA (with a single point mutation for enhanced biochemical stability) in the outward open state, highlighting interesting differences to other proton-coupled drug efflux transporters. The study also includes functional data from a bacterial survival assay testing the resistance towards cationic antimicrobials, as well as ethidium efflux assays for a series of mutant and chimeric variants and combines this with models of the transporter in other conformational states (occluded and inward open states) generated by AlphaFold2 and MD simulations. The two camelid nanobodies (ICabs) used as fiducial markers to enable high resolution structure determination of this 80 kDa membrane protein complex are also functionally characterized but were shown to not have any effect on drug efflux activity mediated by QacA in E. coli spheroplasts. The main finding of this work is that a conserved, helical hairpin-shaped extracellular loop (EL7) within the C-terminal 6TM-bundle of QacA is a unique feature and essential for drug efflux by this branch of the DHA2 family of drug proton antiporters. Potential mechanistic explanations for its critical role in transport are presented, based on the functional analysis of a deletion variant and chimeric transporters with grafted EL7 from other DHA2 family members. The cryo-EM structure of QacA suggests inter-bundle interactions of EL7 with another extracellular loop in the N-bundle (EL1) and MD simulations are utilized to predict putative conformational changes in these loops during different steps of the transport cycle. In addition, several mutants with alanine replacements of residues at the predicted interface are functionally characterized, showing that multiple mutations at this EL1/EL7 interface are required to disrupt ethidium efflux activity. Overall, the findings from this study are well-supported by the experimental data and are presented in an understandable manner to the broader audience. The cryo-EM data is solid and the map quality shown in Figure S5 is sufficient for building the model of QacA. A slightly higher resolution could potentially be achieved by data collection with a defocus range between -0.8 and -2.5 μm (instead of the -1 to -4 range reported in Table S1). However, the defocus range was probably chosen to enhance contrast due to the small size of the nanobody/QacA complex and the achieved resolution is sufficient to support the presented data, since no interactions of the transporter with small molecules in distinct binding poses are reported here.
One limitation of this manuscript is that the D411N variant used to enable purification of monodisperse protein for cryo-EM studies only retains the ability to transport certain divalent cationic substrates but loses the ability to transport monovalent cationic substrates. Furthermore, no cryo-EM structures have been solved in the presence of any of these antimicrobial drugs, hence the results do not provide any direct insights into the molecular interactions with cationic substrates. This makes the structural data less useful for drug discovery efforts utilizing virtual screening techniques to develop new antimicrobial "additives" designed to prevent efflux of cationic antibacterial drugs via QacA when treating infectious diseases caused by multi-resistant strains of Staphylococcus aureus. The work is suitable for publication in The EMBO Journal, but I would suggest a number of changes that would enhance the readability of this manuscript:

Major points:
1.) The manuscript would benefit from shortening introduction, results and discussion because summaries of the main findings are included in all three sections which is repetitive and a more concise description would be easier to follow. Conversely, "motif B" discussed in the result section has not been mentioned in the introduction.
2.) In the first page of the introduction, the text should be rephrased because the word "diverse" is used very frequently in many sentences.
3.) In the result section (page 7), differences in the interdomain cleft between TM5 and TM8 and "exposure to the lipid bilayer allowing interactions with amphipathic molecules like detergents through the bilayer" compared to other DHA members are mentioned but the functional relevance of this observation is not further explained or discussed.
4) The discussion is quite short and is not going into detail about mechanistic conclusions that can be drawn from the results. For example, one aspect is the relative mobility of N- and C-terminal bundles and how this may be influenced by the presence of the unique hairpin-shaped EL7 and its interaction with EL1. For this, a superposition of TM1-6 and TM9-14 bundles of the outward open structure and inward-open model of QacA as additional Appendix Figure would be interesting, because it would show which of the two bundles is more mobile (undergoing larger conformational changes upon transition from outward open to inward open state) or if it has symmetric movements of both bundles like LacY and MdfA. For example, for the structures of PepT family members PepT1 and PepT2 such structural overlays have helped to show that C -terminal bundle is more rigid (Fig 3C in Killer et al Sci Adv. 2021).

Minor points:
1.) Abstract: "chimeric constructs of QacA built by translocating" rephrase to "chimeric constructs of QacA with helical hairpin grafted from another DHA2 member...,"
2.) Introduction, page 5: change "A diverse array of pumps and transporters are employed" to "A diverse array of pumps and transporters is employed" (singular)
3.) Introduction, page 6: "We employed these ICabs to perform electron cryomicroscopy" -> the term "cryo-electron microscopy" (cryo-EM) is much more commonly used
4.) Page 9, Figure S7 callout, replace Appendix Fig. 7 A-C with Fig. S7 A-C
5.) Page 11, section about chimeric constructs: "We surmise that this discrepancy in the behavior is the result of the greater sequence similarity among QacA and LfrA EL7 hairpins" -> add the percentage of sequence identity as a number
6.) Figure legends: a mixture of present and past tense is used. Present tense makes more sense, eg Figure 1 A, B: "...size exclusion profiles of QacA D421A mutant showed a more homogenous profile" -> change to "show"
7.) Figure 1 C legend explain what the abbreviation "CFU" stands for
8.) Figure 1 F legend: add a sentence to make clear that these are flow cytometry output diagrams
9.) Figure 1 H legend. Add details about how the 2D classes were created (how many particles, how many iterations od 2D classification). Is this data from a Talos Arctica screen or the final Titan Krios collection? Maybe refer to Suppl. S4 with EM workflow
10.) Figure 3 C , first panel: add a label for TM2 with Y63
11.) Figure 5 B: adding slab views (like in Figure 3 A) would make it easier to see occluded and inward open conformations of the two models generated by AF2 and MD respectively.
12.) Figure 5 C, D: please add labels for the TM helices, EL1 and EL7 for better clarity
13.) Figure S4 A: add a scale bar to the cryo-EM micrograph
14.) Figure S6: add info about the algorithm used for the sequence alignment
15.) Figure S7 B and C: please label CDRs 1-3 of the nanobody and display them in different color shades and define color scheme in the Figure legend and add similar color coding for the labeling of CDRs 1-3 to Figure S2 B (sequence info for the nanobodies)

Referee #3:

The manuscript by Majumder et al. presents a cryo-EM structure of QacA, a proton-coupled efflux transporter of antibacterial compounds that contributes to the drug-resistance mechanism of an important human pathogen. Of note, QacA is biochemically unstable and the authors found a functional mutant with improved stability that, along with two different camelid nanobodies that bind the surface of the transporter, enabled cryo-EM structure determination.
This work constitutes the first high-resolution structure of QacA, displaying an outward-facing state of the transport cycle and highlights a novel architectural feature of the transporter, namely the extracellular helical-hairpin loop EL7, as well as speculates on its role in the transport mechanism. The cryo-EM map and structure are of good quality, and the structural data are nicely complemented with mutagenesis and functional analysis, as well as MD simulations. The text is concise and clear for the most part, but see minor comments, and the figures are nicely done to support the scientific claims.
Overall this structural and functional study will be of interest to the membrane transport and microbiology communities. However, I have some major and minor comments that should be considered before publication.

Major comments:

-It is striking the presence of a sizeable extracellular domain like EL7, however its role in transport function is not totally clear from the data in the manuscript. On one side, MD simulations and alphafold modelling of occluded and inward-facing states,

respectively, suggest that EL7 might interface with the N-term domain of the transporter (EL1) to isomerize into the inward-facing state. However, single-point mutations at predicted amino acid contacts have no effect on function, and only a penta-alanine mutant showed partial effect. More importantly, the nanobody (Nb) B7 appears to bind, at least partly to the predicted interacting interface on the EL7 side, and yet that Nb doesn't have an effect on function. How can the authors explain the latter result? It would be important to describe in more detail the presence of steric clashes and/or the burying effect of the Nb B7 on interfacial amino acids in the occluded and inward-facing models.

-Regarding the Et efflux assay. It is a key functional assay in the manuscript and it would be nice to see a positive control for QacA-mediated efflux inhibition using a known molecule from the literature, assuming such an inhibitor exists. Moreover, the experiment in Fig. S7D showing lack of effect of the Nbs is a key experiment that contains important mechanistic information, and it should be included in the main text.

-I could not find in the manuscript the details on how protein expression was determined, beyond some mention to western blotting using anti-his antibodies. Could this method also detect non-functional protein in inclusion bodies? Please, clarify this point in the methods. I think this is important because the functional effect of EL7 deletion is based on the fact that it doesn't affect QacA expression.

- Regarding cryo-EM data processing. It is rather surprising that the authors did not attempt to run 3d classification jobs (ab initio and/or heterogenous refinement). In our own experience, 3D classification pipelines yield better particles sets (higher-quality maps) than pipelines where particles were selected based on 2D classification only. I advise the authors to run such jobs in search for better quality maps, as well as potential conformational heterogeneity. Regarding this, from the 2D classes of the single-nanobody sample, it seems that the data is of enough quality to generate 3D reconstructions, and spot potential differences in QacA structure bound to a single Nb. Have the authors explore this possibility?

Minor comments:

Page 4: Acidic residues "...undergo protonation to facilitate enhanced stoichiometry for drug:proton antiport...", this sentence is rather ambiguous. What is "enhanced stoichiometry"? Does the stoichiometry of H:substrate change asmong different substrates?
Also, the last paragraph on this page just echoes the abstract and I would shorten it to a brief sentence stating what the study aims at.

Page 7: at the top, why is the size of QacA vestibule being compared to DHA1 transporters, and not to DHA2 ones?

Page 9: Third paragraph, please rephrase "this is the primary site where most binders interact". Also, the reported Kd values are apparent ones, please clarify that. In addition, the way the apparent Kd values are reported for Nb A4 and B7 leaves the erroneous impression that A4 is a potent binder and B7 is not. The differences in apparent Kd are marginal (60 vs 100 nM), plus it is unclear if the assay used in the manuscript accurately distinguishes small differences in apparent Kd.

Page 16-methods: in the sample preparation section, it is unclear why the authors performed preliminary data collection on a Krios, then they did the grid screening on a Talos, and the final data collection on a Krios-K3 setup. Please, explain the rationale for preliminary data collection on a krios before screening grids, or remove the sentence.

EMBOJ-2023-113418: Responses to Reviewers.
Referee #1:

This manuscript by Majumder et al. reports the cryoEM structure of QacA, which mediates proton-coupled efflux of mono and divalent cationic antibacterial compounds in Staphylococcus aureus. The authors inserted a single mutation D411N to improve homogeneity - without abolishing dequalinium and chlorhexidine efflux activity - and resolved the structure of substrate-free QacA in an outward open conformation to a resolution of 3.6 Å. QacA exhibits an extracellular helical hairpin loop between TMs 13 and 14, which is conserved in a subset of drug:proton antiport (DHA) transporters. They demonstrate that removal of the EL7 hairpin loop compromises efflux activity and that chimeras between QacA and LfrA exhibit reduced efflux activity. They conclude that EL7 hairpin allosterically regulates efflux in QacA.
This is an interesting manuscript that reports the combination of a large variety of methods to understand a biologically very important transporter. My main concerns are the sparseness in the methods description and the overall lack of a clear conclusion of this work. Is it really necessary to combine cryo-EM, MD simulation, recombinant DNA technology, functional data to conclude that an external hairpin fulfills an allosteric and crucial role for function? I would expect a clear mechanistic conclusion in such work..
Response: We thank the reviewer for summarising the major results of the work and finding this work to be of interest and focused on an important transporter. We have addressed the sparseness in the methods by describing the individual methods in much greater detail and provided important and specific details of the experiments done in the revision.
We have also altered the discussion to bring out mechanistic aspects of QacA function and how the present study's results delve into the transport process of QacA and the role of structural features like the EL7 to drive transport. We have rewritten a major part of the discussion to highlight a mechanistic role of QacA function with regards to protonation driven antiport and associated conformational changes comprsing the transport cycle. The presence of EL7-EL1 interface is further highlighted by additional experiments as described in the results and discussion.

1. P. 7, Fig. 3, The authors use APBS to determine electrostatic potentials. Please describe the procedures in the Method section. Moreover, please discuss the limitations of APBS.
Response: The procedures for electrostatic surface generation is described in greater detail in the methods as a separate section as follows,
"APBS electrostatic maps preparation and representation
To visualize the general electrostatic environment, coordinates of QacA (this study), MdfA (PDB ID: 6GV1), LmrP (PDB ID: 6T1Z) and YajR (PDB ID: 3WDO) were parsed through the Adaptive Poisson-Boltzmann Solver (APBS) plugin in PyMOL v2.5.0, that uses AMBER ff99 forcefield. A gradient from red to blue through white colours was used to represent a ramp of -10 to +10 $kBTe_c^{-1}$ in the generated electrostatic maps. All acidic and basic residues were considered solvent exposed and hence charged, and their relative solvent accessibility was not derived from the structure for this purpose. The APBS uses known dielectric constants and local electric potentials for bulk water, organic molecules and many ions for implicit solvation and calculating electric fields around the macromolecule."

The limitations of APBS are also discussed in the following lines in the results: The following sentence was added to the section in page 7 to highlight limitations of APBS, "Although the use of APBS is fraught with some shortcomings like overestimating dielectric at solvated regions with low solvent exchangeability (which also affects the protonation/deprotonation propensity of partially buried residues), the method can be robustly used to visualize distribution of charged residues in the membrane".

2. The reported electrotstatic potentials are significantly more negative than in related transporters. Please discuss potential consequences of this feature! Could it be that non-subtrate cations, such as Na^+ and K^+, accumulated in the vestibule? Are these transporters Na+ or K+ dependent? Are there functional data in the presence and in the absence of small cations? Are the positive electrostic potential a hallmark of drug:proton antiporters in the MFS family?

Response: The reviewer is right in suggesting the surface charge within QacA as more negative than related DHA1 transporters. The presence of multiple acidic residues provides a flexibility of interactions for protons and substrates and plays a role in the promiscuous uptake of antibacterial substrates in QacA as we establsihed in our previous study on this transporter in Majumder et al., 2019, J. Mol. Biol. We used MD simulations and have discussed this in the results section in the folowing lines. "The larger number of acidic residues within the QacA vestibule renders the vestibular surface electronegative to facilitate attraction of protons from the extracellular medium that can be exchanged for cationic antibacterials in the cytosol. This introduces the possibility of localization of metal ions in the vestibule of QacA. Indeed, through MD simulations of QacA in lipid bilayer, we observe an enrichment for both Na^+ and K^+ ions but not Cl^-, at the base of the vestibule, surrounded by E406, E407 and D411 (Appendix Fig. S5A). To assess whether Na^+ can induce proton efflux in QacA, we performed everted vesicle assay with various concentrations of sodium gluconate and found that QacA was not able to transport sodium ions in comparison to empty vesicles lacking QacA (Appendix Fig. S5). Moreover, in the presence of Na^+ , QacA retained the ability of TPP to induce proton efflux (Appendix Fig. S5B). The interactions of Na^+ or K^+ ions are likely to have a very low affinity as we had previously established that ~1000 times lower concentrations of cationic antibacterial compounds can compete for and release protons in purified QacA despite the presence of 200 mM NaCl (Majumder et al., 2019)."

3. P. 10, Fig. 5. The authors performed equilibrium MD simulations to elucidate a transition from the outward-open QacA structure to the inward-facing occluded state. Obviously, the transporter returns in the apo state from the outward into the inward facing conformation. This should not be possible when the proton-acceptor - necessary for drug-proton antiport is protonated. Did the authors try this?

Response: DHA members when present in the open-to-out state interact with protons in the periplasmic compartment or cell wall milieu (low pH) resulting in protonation of acidic residues in the vestibule. The protonation step induces a conformational transition to occlude the outside-open vestibule. Further transition to open-to-in state results in release of protons upon exposure to the higher pH of the cytosol and possibly through competition against higher affinity substrates.

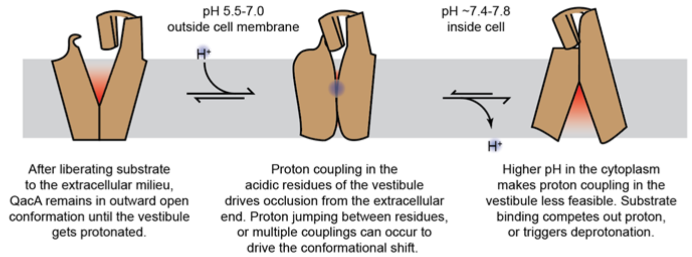

This leads to shift from open-to-in to outward-open state, releasing the substrate and completing the cycle. What we report in our study is a simulation of the early stages of

the protonation induced conformational shift from outward open state to an occluded state. It is relatively easier to predict such a mechanism when a single residue in the vestibule is involved in both proton and substrate coupling, which is not the case in QacA. However, by simply coupling D411 with a proton in simulation runs, we were able to visualize QacA reaching an occluded state, even if a transition to an inward open state was not found in the simulation time scale studied. This is further consistent with the fact that deprotonated D411 in QacA is unable to induce an occluded state in the simulations. To further prove that the conformational transition to an occluded state reported through MD simulations in this study is indeed dependent upon D411 protonation, we initiated unbiased equilibrium MD runs using one of the occluded frames captured in these runs, this time reverting the D411 sidechain to a deprotonated state. We found that during the course of these runs, the only conformational transition that QacA undergoes is back to the outward open state, suggesting the proton-dependent reversibility within the MD simulation (see below panel).

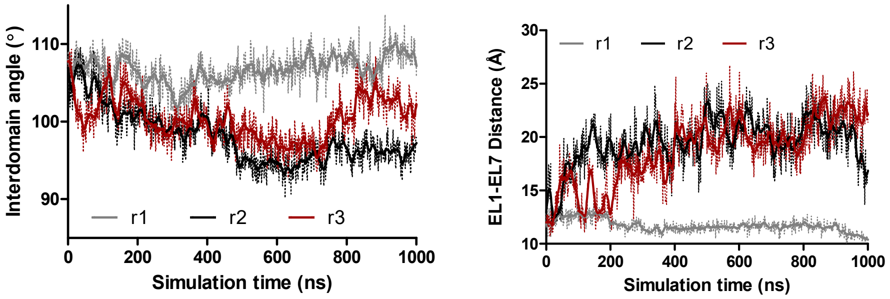

Here interdomain angle measures the angle made by the N and C terminal domains on the vestibule. EL1-EL7 distance measures the centers of masses of the Cα atoms in the two loops. A more descriptive illustration for these graphs is provided later in the following page.

4. How was this simulation exactly done? Is there any substrate present? How often such simulations were performed? Are there any statistics? What is the conclusion?

Response: The simulation was performed in lipid bilayer made of POPE:POPG in roughly 3:1 molar ratio, as already stated in the methods section (detailed methodology figure below). The simulation boxes was set up in with D411 present either in a protonated state, or a deprotonated state. A control run with D411N was also carried out. The cryoEM structure elucidated in this study was used to mutate residue 411 back to aspartate *in-silico*. All these simulations were run in triplicates, with independent random seeds. No substrates were used in any of the simulation boxes, as we knew that a transition from outward to inward open state should not require the presence of a substrate in the vicinity. To assess degree of occlusion of the vestibule, we monitored the distance between a group of Cα of residues present in the extracellular loops EL1 and EL7 of the N- and C-terminal bundles respectively. To monitor its correlation with the degree of conformational change in the entire transporter, we monitored the angle between the center of masses (COMs) of a bunch of Cα of residues in the extracellular side of the N-terminal bundle, cytosolic side of the N-terminal bundle, and extracellular side of the C-terminal bundle (described as 'Interdomain angle' in the study). While the angle between COMs proved to have a good correlation with the distance plot explained above, the latter plot was used to predict possible interactions between EL1 and EL7 as it was more sensitive to capturing such interaction snapshots. Comparative analyses between D411 protonated and deprotonated versions were carried out. No prominent conformational transitions were observed in simulations with deprotonated D411 in any

of their runs, while we could observe significant shifts toward occlusion of extracellular access to the vestibule in the runs where D411 sidechain was modelled as protonated.

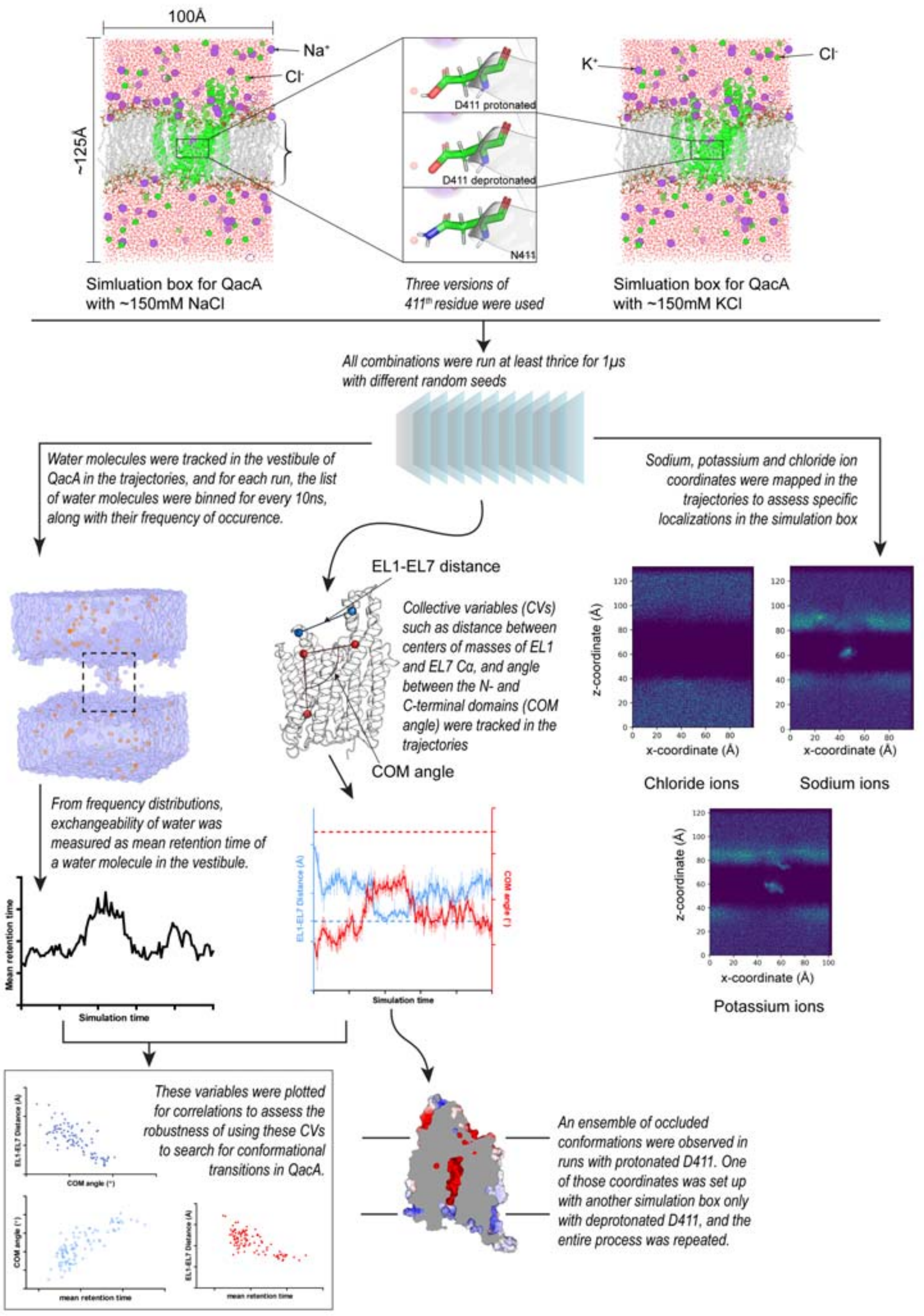

5. How was the AlphaFold2 prediction of the inward-facing conformation performed?
Response: The model of the inward open QacA structure was obtained through the use of Colabfold v1.5, which is available as a free open source code. The model was checked for Ramachandran outliers and clashes.

6. The authors demonstrate that deletion of EL7 abolishes efflux transport and that substitution of EL7 from related transporters partially restores activity. This seems to be a

major result. However, I could not find any no mechanistic role of EL7 in this function. Given the high experimental and theoretical efforts of the authors such conclusion is required.
Response: We concur with the reviewer for emphasizing the need to provide a mechanistic inference for the experiments conducted on the role of EL7 grafting on the transport activity of QacA. Firstly, to prove that the effect of such grafting on the function of QacA arises from its virtue to interact with EL1 in the N-terminal domain, in the revised manuscript we provide data for another set of mutants where we have grafted the putative EL1 loops from LfrA and SmvA onto the existing EL7 chimeric constructs. We have shown that since the EL7 of LfrA has a higher sequence similarity with that of QacA, $QacA_{LfrA\ EL7}$ had a higher gain of function than in the case of $QacA_{SmvA\ EL7}$ chimera, that shows negligible activity. Now by grafting EL1 loops of the homologs into QacA as well, we observe that the mutants have a further gain of function, with $QacA_{LfrA\ EL1\text{-}EL7}$ mutant reaching near QacA-WT like activity, while $QacA_{SmvA\ EL1\text{-}EL7}$ shows a stark revival of transport activity. A summarized comparative analysis for the same can be found in Figure EV4C.

For inferring why such an augmentation could have evolved in the transporter, we checked whether presence of these hairpin loops in QacA homologs is correlated with any other property common among them by reviewing AlphaFold2 models of all the transporters that are annotated as members of the DHA2 family in the Transporter Classification Database (TCID 2.A.1.3). We found that, as pointed out by the reviewer, all transporters with a net negative charge of 4 or more (hence a high positive electrostatic potential) are exclusively found with an EL7 hairpin with them, as shown in Figure EV2.

7. p.18: "All the acidic residues that were solvent exposed were kept deprotonated except D411, which was modelled with a neutral side chain." Why was D411 simulated with neutral side chain? To account for the mutation in the expression construct? Please discuss in this context how these transporters are thought to mediate drug-proton antiport?

Response: As noted by the reviewer, since we had observed that D411N mutation provided a much more homogenous construct in micellar isolates during QacA's construct optimization for cryoEM, QacA was simulated with both protonated (by neutral we meant protonated form) and deprotonated forms of D411 residue. In a previous study by our group (Figure 8, Majumder et al., 2019, JMB), we had also shown that drugs like TPP, Dq and Pentamidine can compete out protons from mainly D34 and D411, and D411N mutation causes a significant decrease in such competition-driven proton release.
A detailed description of the transport cycle in drug-proton antiport has also been discussed as part of the manuscript discussion as follows, "A typical efflux cycle involves a competition driven H^+/substrate antiport involving the interactions of protons from the periplasmic space at a lower pH (~6.0-6.5) with the acidic residues in the vestibule that undergo protonation in the outward-open conformation of the transporter (Fig. 6)(Schuldiner, 2014). Protonation of acidic residues coupled with the membrane potential and proton electrochemical gradients force the conformational shift of the antiporter to the cytosol-facing state where a higher pH (~7.5) leads to the deprotonation of the acidic residues. This allows the cationic substrates to interact with the negatively charged deprotonated acidic residues causing the subsequent reorganization of the transporter back to the outward-open state leading to the release and efflux of quaternary antibacterial compounds into the periplasmic space."
On the basis of these prior observations as well as results from our MD simulations and AF2 model, we have proposed a mechanism for QacA's conformational transition

during the conversion of QacA to outward-open to the inward-open state in the transport cycle as part of the schematic in figure 6.

Referee #2:

The manuscript by Majumder et al. describes the structural characterization of a proton coupled drug efflux antiporter of the MFS superfamily with a critical role for antimicrobial resistance of the pathogenic bacterium Staphylococcus aureus. The study presents insights from a 3.6 Å nanobody-complexed cryo-EM structure of the transporter QacA (with a single point mutation for enhanced biochemical stability) in the outward open state, highlighting interesting differences to other proton-coupled drug efflux transporters. The study also includes functional data from a bacterial survival assay testing the resistance towards cationic antimicrobials, as well as ethidium efflux assays for a series of mutant and chimeric variants and combines this with models of the transporter in other conformational states (occluded and inward open states) generated by AlphaFold2 and MD simulations. The two camelid nanobodies (ICabs) used as fiducial markers to enable high resolution structure determination of this 80 kDa membrane protein complex are also functionally characterized but were shown to not have any effect on drug efflux activity mediated by QacA in E. coli spheroplasts. The main finding of this work is that a conserved, helical hairpin-shaped extracellular loop (EL7) within the C-terminal 6TM-bundle of QacA is a unique feature and essential for drug efflux by this branch of the DHA2 family of drug proton antiporters. Potential mechanistic explanations for its critical role in transport are presented, based on the functional analysis of a deletion variant and chimeric transporters with grafted EL7 from other DHA2 family members. The cryo-EM structure of QacA suggests inter-bundle interactions of EL7 with another extracellular loop in the N-bundle (EL1) and MD simulations are utilized to predict putative conformational changes in these loops during different steps of the transport cycle. In addition, several mutants with alanine replacements of residues at the predicted interface are functionally characterized, showing that multiple mutations at this EL1/EL7 interface are required to disrupt ethidium efflux activity. Overall, the findings from this study are well-supported by the experimental data and are presented in an understandable manner to the broader audience.
Response: We thank the reviewer for these positive and constructive comments about our study. We have made the required changes as per the suggestions below for both the major and minor comments.

The cryo-EM data is solid and the map quality shown in Figure S5 is sufficient for building the model of QacA. A slightly higher resolution could potentially be achieved by data collection with a defocus range between -0.8 and -2.5 µm (instead of the -1 to -4 range reported in Table S1). However, the defocus range was probably chosen to enhance contrast due to the small size of the nanobody/QacA complex and the achieved resolution is sufficient to support the presented data, since no interactions of the transporter with small molecules in distinct binding poses are reported here.
Response: As the reviewers rightly pointed out, the defocus range was chosen to enhance contrast of the small particles used in the study bound to QacA. We did try extensively to reprocess and improve the resolution (as suggested by a different reviewer) but the original map reported in the study is the best that was achieved and therefore we retained the original processed data.

One limitation of this manuscript is that the D411N variant used to enable purification of monodisperse protein for cryo-EM studies only retains the ability to transport certain divalent cationic substrates but loses the ability to transport monovalent cationic substrates.
Response: While we see the relvance of the reviewer's point in the context of the D411N mutant being inactive for monovalent cationic antibacterial transport, the $QacA_{D411N}$ can transport a few divalent catinoic substrates effectively. It was essential to have a well behaving construct for our structural studies for QacA and therefore this mutant

was used. Future efforts to understand the functional states and substrate interactions of QacA would involve studies using $QacA_{WT}$ construct.

Furthermore, no cryo-EM structures have been solved in the presence of any of these antimicrobial drugs, hence the results do not provide any direct insights into the molecular interactions with cationic substrates. This makes the structural data less useful for drug discovery efforts utilizing virtual screening techniques to develop new antimicrobial "additives" designed to prevent efflux of cationic antibacterial drugs via QacA when treating infectious diseases caused by multi-resistant strains of Staphylococcus aureus.
Response: We appreciate the reviewer pointing this issue and we are also aware of this limitation. However the structural part of the study was facilitated by the rather remarkable improvement of the sample behavior upon making this mutant in comparison to the QacAwt. With the availability of the antibodies it would be easier to explore the substrate interactions, alternate conformation of QacA in the near future.

The work is suitable for publication in The EMBO Journal, but I would suggest a number of changes that would enhance the readability of this manuscript:
Response: We thank the reviewer for providing a balanced asessment of the manuscript and considering this study to be suitable for EMBOJ. We have made all the necessary changes as suggested and improved the quality of the mansucript as per the following suggestions of the reviewer.

Major points:
1.) The manuscript would benefit from shortening introduction, results and discussion because summaries of the main findings are included in all three sections which is repetitive and a more concise description would be easier to follow. Conversely, "motif B" discussed in the result section has not been mentioned in the introduction.
Response: We have shortened the introduction and mentioned the presence of motifs in DHA transporters. We altered the results with newer experiments to bolster our findings and rewrote the discussion more extensively to bring in mechanistic insights to decipher the transport mechanism of QacA in antibacterial efflux. Motif B is now briefly mentioned in the introduction along with the other conserved motifs observed in DHA members in the following line of introduction, "QacA also retains sequence conserved sequence motifs A, B and C that are characteristic of DHA and other MFS transporters."

2.) In the first page of the introduction, the text should be rephrased because the word "diverse" is used very frequently in many sentences.
Response: We have rephrased the sentences to minimize usage of the word "diverse". We thank the reviewer for pointing out this redudancy.

3.) In the result section (page 7), differences in the interdomain cleft between TM5 and TM8 and "exposure to the lipid bilayer allowing interactions with amphipathic molecules like detergents through the bilayer" compared to other DHA members are mentioned but the functional relevance of this observation is not further explained or discussed.
Response: We have added an additional sentence in the results section to explain the siginificance of the two helices in the transport cycle as follows in page 7 para1 , "When compared with the inward open state of QacA AlphaFold2 model the TM5 undergoes an angular shift away from TM10 that remains unaltered. The movement of TM5 and TM1 at the cytosolic face allows the QacA to form a cytosol-facing conformation."

4) The discussion is quite short and is not going into detail about mechanistic conclusions that can be drawn from the results. For example, one aspect is the relative mobility of N- and C-terminal bundles and how this may be influenced by the presence of the unique hairpin-shaped EL7 and its interaction with EL1. For this, a superposition of TM1-6 and TM9-14

bundles of the outward open structure and inward-open model of QacA as additional Appendix Figure would be interesting, because it would show which of the two bundles is more mobile (undergoing larger conformational changes upon transition from outward open to inward open state) or if it has symmetric movements of both bundles like LacY and MdfA. For example, for the structures of PepT family members PepT1 and PepT2 such structural overlays have helped to show that C -terminal bundle is more rigid (Fig 3C in Killer et al Sci Adv. 2021).

Response: As suggested by the reviewer we have extensively altered the discussion to introduce mechanistic roles for the findings in this manuscript and introduced the presence of assymetric rocker switch in the QacA in comparison to symmetric LacY and MdfA transporters (figure EV 5). We thank the reviewer for the suggestion on the PepT1 and PepT2 study and have cited the Killer etal reference to support our case of an asymmetric rocker-switch in QacA.

Minor points:
1.) Abstract: "chimeric constructs of QacA built by translocating" rephrase to "chimeric constructs of QacA with helical hairpin grafted from another DHA2 member...,"
Response: We have altered the phrase as suggested.
2.) Introduction, page 5: change "A diverse array of pumps and transporters are employed" to "A diverse array of pumps and transporters is employed" (singular)
Response: **Altered as suggested.**
3.) Introduction, page 6: "We employed these ICabs to perform electron cryomicroscopy" -> the term "cryo-electron microscopy" (cryo-EM) is much more commonly used
Response. We have altered the phrase as suggested.
4.) Page 9, Figure S7 callout, replace Appendix Fig. 7 A-C with Fig. S7 A-C
Response. We made the alteration and as per journal format we call all figures as Appendix Fig. S1 etc.
5.) Page 11, section about chimeric constructs: "We surmise that this discrepancy in the behavior is the result of the greater sequence similarity among QacA and LfrA EL7 hairpins" -> add the percentage of sequence identity as a number
Response: Percent sequence similarity numbers have been added in parentheses in EL7. We have added the sequence similarity between the two pumps in the statement prior to discussion in the lines as follows, "We found the EL7 of QacA to possess greater sequence identity with LfrA EL7 hairpins (33 %), in comparison to SmvA (24%) (Fig. 5F, Fig. EV4A), hence providing a greater probability of re-establishing the interactions between LfrA-EL7 and QacA-EL1."
6.) Figure legends: a mixture of present and past tense is used. Present tense makes more sense, eg Figure 1 A, B: "...size exclusion profiles of QacA D421A mutant showed a more homogenous profile" -> change to "show"
Response: Legend altered to say "shows"
7.) Figure 1 C legend explain what the abbreviation "CFU" stands for
Response: CFU has been expanded to "colony forming units" in figure 1c. The change is included.
8.) Figure 1 F legend: add a sentence to make clear that these are flow cytometry output diagrams .
Response: The legend has been modified to indicate that the data is from flowcytometry out puts. The sentence reads as follows, "Icab binding analysis performed using populations shifts through FACS using yeast populations…".

9.) Figure 1 H legend. Add details about how the 2D classes were created (how many particles, how many iterations od 2D classification). Is this data from a Talos Arctica screen or the final Titan Krios collection? Maybe refer to Suppl. S4 with EM workflow.

Response: 2D classes were created for QacA-A4 complex with data collected on a Titan Krios with a K2 camera. Processing was done using Cryosparc-3.0 with data collected at the national CryoEM facility at NCBS-Instem, Bangalore. No data was collected on the Talos Arctica. The single particle data used for the final structure was collected at EBIC-Diamond light source. As suggested legend for 1F was altered as follows, "Representative reference-free 2D classes of $QacA_{D411N}$ embedded in UDM micelle, in complex with ICab A4 (top) and with ICabs A4 and B7 (bottom), pointed with pink and green arrows respectively. Representative classes shown for QacA-A4 complex are from a set of 68,000 particles and QacA-A4/B7 complex are from 218,040 particles.

10.) Figure 3 C , first panel: add a label for TM2 with Y63
Response: Label added.
11.) Figure 5 B: adding slab views (like in Figure 3 A) would make it easier to see occluded and inward open conformations of the two models generated by AF2 and MD respectively.
Response: Sagittal sections of the molecules have been added to represent different states in Fig. 5A.
12.) Figure 5 C, D: please add labels for the TM helices, EL1 and EL7 for better clarity
Response: Appropriate labels added for panel 5B.
13.) Figure S4 A: add a scale bar to the cryo-EM micrograph
Response: A new micrograph image with scale bar is included.
14.) Figure S6: add info about the algorithm used for the sequence alignment
Response: The following information about the algorithm was added to the Appendix figure S6. legend, "Alignment was performed using clustal omega suite with 5 HMM iterations. Jalview was used for data visualization."
15.) Figure S7 B and C: please label CDRs 1-3 of the nanobody and display them in different color shades and define color scheme in the Figure legend and add similar color coding for the labeling of CDRs 1-3 to Figure S2 B (sequence info for the nanobodies)
Responses: CDRs have been labelled for the Icabs and different coloring is used in the figure which is now moved to Figure EV3 as suggested by one of the reviewers. Accordingly CDR regions have been colored in the sequence alignment in Appendix Fig. S2B.

Referee #3:

The manuscript by Majumder et al. presents a cryo-EM structure of QacA, a proton-coupled efflux transporter of antibacterial compounds that contributes to the drug-resistance mechanism of an important human pathogen. Of note, QacA is biochemically unstable and the authors found a functional mutant with improved stability that, along with two different camelid nanobodies that bind the surface of the transporter, enabled cryo-EM structure determination.
This work constitutes the first high-resolution structure of QacA, displaying an outward-facing state of the transport cycle and highlights a novel architectural feature of the transporter, namely the extracellular helical-hairpin loop EL7, as well as speculates on its role in the transport mechanism. The cryo-EM map and structure are of good quality, and the structural data are nicely complemented with mutagenesis and functional analysis, as well as MD simulations. The text is concise and clear for the most part, but see minor comments, and the figures are nicely done to support the scientific claims.
Overall this structural and functional study will be of interest to the membrane transport and microbiology communities. However, I have some major and minor comments that should be considered before publication.

Response: We thank the reviewer for a supportive and favourable assessment of this study. We have listed out our responses to both the major and minor comments of the reviewer in the responses below.

Major comments:

-It is striking the presence of a sizeable extracellular domain like EL7, however its role in transport function is not totally clear from the data in the manuscript. On one side, MD simulations and alphafold modelling of occluded and inward-facing states, respectively, suggest that EL7 might interface with the N-term domain of the transporter (EL1) to isomerize into the inward-facing state. However, single-point mutations at predicted amino acid contacts have no effect on function, and only a penta-alanine mutant showed partial effect. More importantly, the nanobody (Nb) B7 appears to bind, at least partly to the predicted interacting interface on the EL7 side, and yet that Nb doesn't have an effect on function. How can the authors explain the latter result? It would be important to describe in more detail the presence of steric clashes and/or the burying effect of the Nb B7 on interfacial amino acids in the occluded and inward-facing models.

Response: The reviewer makes valid points concerning the role of EL7 in transport properites of QacA. The MD and Alphafold 2 models of inward facing QacA display the presence of a EL1 and EL7 interface formation where single substitutions do not effect the transport properties. This is consistent with the observation that a EL7-EL1 interface has to be formed through multiple residue interactions at the interface. From our results it is evident that disrupting one residue at a time has little or no effect on the activity of the transporter. Disruption of the complete interface through the 5 Ala mutant however is observed to have a clear loss of efflux activity suggesting that minor substitutions at the EL1-EL7 interface can still sustain efflux whereas complete removal of this interfacial interactions cause compromised activity.

With regards to the lack of effect of the two antibodies A4 and B7 on transport activity, we were equally confounded by this result and probed the structural transitions through comparison of the outward open state with the inward-open model of QacA by overlapping the EL7 hairpin loop between the two states. The Icab A4 interacts at the extended regions of TM13 and TM14 away from the EL7 hairpin thus avoding any interference in the conformational shifts associated with efflux activity. On the contrary B7 Icab interfaces primarily with helical regions of the hairpin EL7b with an epitope interface much smaller than A4. The CDR2 loop has minor clashes in the region of EL1 at residues 49-51 that forms the end of TM1. The rest of the EL1 from 52-56 is still capable of interacting with EL7 to facilitate trasport. We presume that the presence of B7 does not have a major block of QacA's efflux activity as the EL7 and EL1 interface can still form partially if not completely to facilitate transport. We have discussed these points in the results section, page 11 as follows, "The ICab A4 binds to the TM13 and TM14 helices that protrude out of the bilayer and does not hamper interactions required for rocker-switch motions in QacA. However, B7 interactions that happen at the EL7 hairpin region could have interfered with the transport process. Upon further analyses by overlapping the hairpin loops of the AlphaFold2 $QacA_{io}$ structure on the $QacA_{oo}$, we could observe minor side chain clashes in a part of EL1 region (residues 49-51) with the CDR2 of B7 (residues 55-57) whereas the remaining EL1 and EL7 interface formation remains unhampered (Fig. EV3E)".

-Regarding the Et efflux assay. It is a key functional assay in the manuscript and it would be nice to see a positive control for QacA-mediated efflux inhibition using a known molecule from the literature, assuming such an inhibitor exists. Moreover, the experiment in Fig. S7D showing lack of effect of the Nbs is a key experiment that contains important mechanistic information, and it should be included in the main text.

Response: Reserpine was proposed as an inhibitor of QacA in an earlier study (Mitchell et al., 1999) wherein it blocks ethidium efflux effectively. We also performed the efflux assay to demonstrate that reserpine can effectively block efflux in whole cells. We applied reserpine as a positive control as suggested to spheroplasts and blocked the efflux suggesting that B7 and A4 do not block efflux of QacA Appendix Fig. S10. The

primary spheroplast experiment is now included in expanded view figure 3.

-I could not find in the manuscript the details on how protein expression was determined, beyond some mention to western blotting using anti-his antibodies. Could this method also detect non-functional protein in inclusion bodies? Please, clarify this point in the methods. I think this is important because the functional effect of EL7 deletion is based on the fact that it doesn't affect QacA expression.

Response: The protein levels were detected using western blot using an anti-His tag. For whole cell experiments the Westerns do have a risk of assessing the aggregated protein in inclusion bodies. However we observe that both in Westerns done for whole cells and for blots done in membrane fractions (spheroplasts and everted vesicles) we do not observe a difference between $QacA_{WT}$ and $QacA_{EL7}$ expression(Fig. S8 blots) suggesting that the loss of protein into inclusion bodies is rather minimal if any.

As suggested we have mentioned this in the methods part of the manuscript where we have added a detailed new section titled "Western blots to analyse expression of QacA constructs in whole cells/membrane vesicles".

- Regarding cryo-EM data processing. It is rather surprising that the authors did not attempt to run 3d classification jobs (ab initio and/or heterogenous refinement). In our own experience, 3D classification pipelines yield better particles sets (higher-quality maps) than pipelines where particles were selected based on 2D classification only. I advise the authors to run such jobs in search for better quality maps, as well as potential conformational heterogeneity.

Response. As suggested we performed 3D classification jobs for both a larger particle set (502364 particles) and the particle set used for the reported structure (218040) to employ heterogenous refinements to observe if there is any improvement in the quality of the maps. In the first instance five classes were built through abinitio reconstruction followed by heterogenous refinement of three classes that looked close to QacA structure. This was followed by homogenous refinement of two classes followed by non-uniform refinement that yielded a final resolution of about 3.9 Å for a particle set of 264168 particles. (figure below)

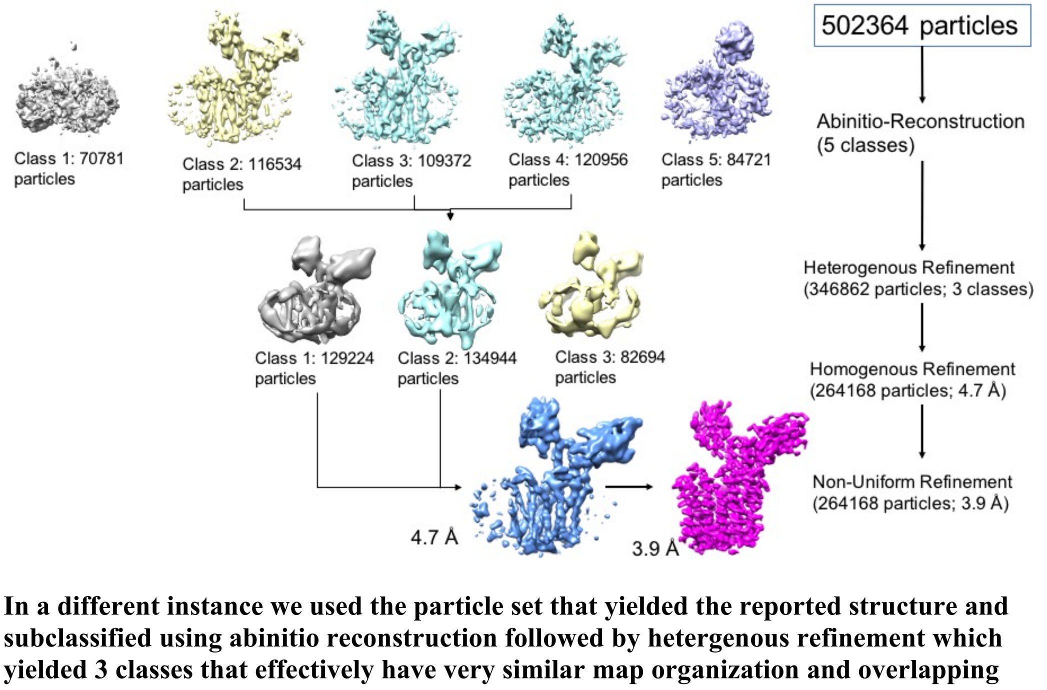

In a different instance we used the particle set that yielded the reported structure and subclassified using abinitio reconstruction followed by hetergenous refinement which yielded 3 classes that effectively have very similar map organization and overlapping similarity when compared with the refined structure. We further refined each of these classes using homogenous and non-uniform refinement steps and observed a highest resolution limit of 4.1 Å for a subset of 80K particles (below). Even at this stage the

three classes had very similar structural organization which justified our using the complete particle set of 218040 particles for the reconstruction of QacA that yielded the best resolution thus far of 3.8 Å.
Since both approaches yielded similar to lower resolution reconstruction we decided to retain our previous map and refinement.

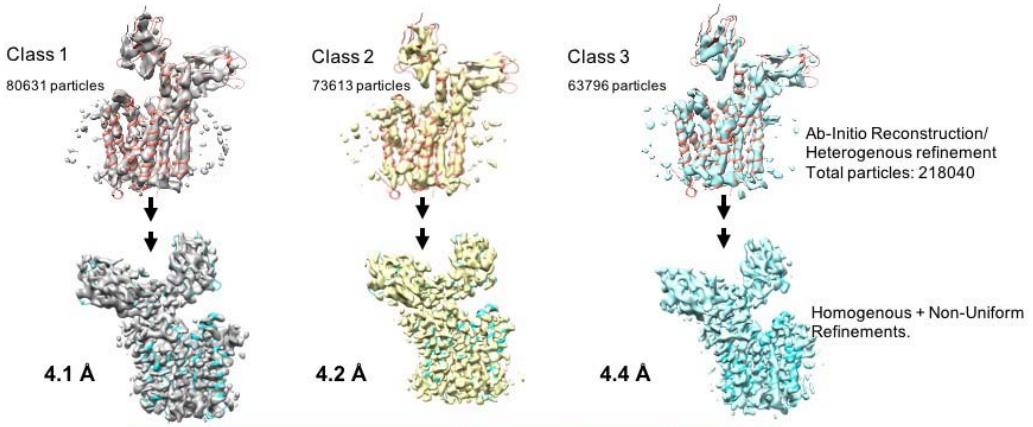

	Class 1	Class 2	Class 3
Particle numbers	80631	73613	63796
Abinitio Reconstruction /Heterogenous Refinement	7.1Å	7.3 Å	7.7 Å
Homogenous Refinement	5.4 Å	6.3 Å	6.7 Å
Non-uniform refinement	4.08 Å	4.2 Å	4.4 Å

Regarding this, from the 2D classes of the single-nanobody sample, it seems that the data is of enough quality to generate 3D reconstructions, and spot potential differences in QacA structure bound to a single Nb. Have the authors explore this possibility?
Response: The 2D classes of the single nanobody sample are indeed of a good quality. However despite clean classes the reconstructions from this relatively small data set of particles we could not get a reasonable reconsrtuction despite several attempts through cryosparc and/or relion likely due to a relatively low particle number of around 65000. The reconstruction required a much larger dataset collected at EBIC-Diamond used in the study. However we do understand the potential use of the QacA-A4 complex and would use this to explore other conformations and substrate complexes in future studies.

Minor comments:

Page 4: Acidic residues "...undergo protonation to facilitate enhanced stoichiometry for drug:proton antiport...", this sentence is rather ambiguous. What is "enhanced stoichiometry"? Does the stoichiometry of H:substrate change asmong different substrates?
Also, the last paragraph on this page just echoes the abstract and I would shorten it to a brief sentence stating what the study aims at.
Resposne: We replaced this statement with a more accurate description of the findings from Majumder etal. 2019 as follows, "A homology model of QacA displays six acidic residues (D34, D61, D323, E406, E407, and D411) within the solvent-accessible vestibule of the transporter that play diverse roles in the promiscuous antibacterial efflux properties of QacA". It is known that DHA transporters that different substrates can have different proton:substrate stoichiometries. We have further altered the last paragraph of introduction and have written it differently to avoid similarity with the abstract.

Page 7: at the top, why is the size of QacA vestibule being compared to DHA1 transporters, and not to DHA2 ones?
Response. The QacA structure in this study is the only DHA2 member whose structure is available. Alternately the DHA1 transporters have multiple structures that are experimentally elucidated and purported to have a similar mechanism of antiport. It is therefore only appropriate to compare the experimentally derived structures of QacA with DHA1 members in outward-open states like MdfA and LmrP.

Page 9: Third paragraph, please rephrase "this is the primary site where most binders interact". Also, the reported Kd values are apparent ones, please clarify that. In addition, the way the apparent Kd values are reported for Nb A4 and B7 leaves the erroneous impression that A4 is a potent binder and B7 is not. The differences in apparent Kd are marginal (60 vs 100 nM), plus it is unclear if the assay used in the manuscript accurately distinguishes small differences in apparent Kd.
Response: The sentence has been rephrased as follows, "epitope where most of the binders interact". We indicated that the difference in the Kd value between A4 and B7 is marginal at appropriate locations. Since the dissociation constant was estimated by measuring the relative amount of bound QacA at different concentrations of QacA , we have termed the values as apparent affinities.

Page 16-methods: in the sample preparation section, it is unclear why the authors performed preliminary data collection on a Krios, then they did the grid screening on a Talos, and the final data collection on a Krios-K3 setup. Please, explain the rationale for preliminary data collection on a krios before screening grids, or remove the sentence.
Response. The sample for grid preparation had to be extensively optimized with one and two Icabs respectively. A dataset with a single A4 bound QacA was collected at the Krios with a K2 camera at the National cryoEM facility at the Instem, Bangalore. However this dataset was not of sufficient quality and size for a complete reconstruction of QacA. Subsequently QacA with two Icabs complexed was used to prepare grids that were sent to EBIC for data collection. Prior to data collection the grids were screened for particle distribution and quality of ice on a Talos arctica and final data collection of 7770 micrographs was performed at the Krios I at EBIC, Diamond with a 300keV voltage using a K3 detector and an energy filter. This dataset yielded the final structure that is reported in this study. We have clarified the methods section as follows, "Data for $QacA_{D411N}$ complexed with A4 were collected on a Titan Krios equipped with a K2 detector(Appendix fig S). Grid screening for QacAD411N-A4-B7 complex was done using an in house Talos Arctica 200 keV cryo-electron microscope and the grids with good particle distribution in thin ice were sent to the eBIC-Diamond Light Source, UK where a large dataset was collected on a Titan Krios 300 keV electron microscope equipped with Gatan K3 detector."

1st Revision - Editorial Decision 9th Jun 2023

Dear Dr Aravind Penmatsa,

Thank you for submitting your revised manuscript (EMBOJ-2023-113418R) to The EMBO Journal, as well as for your patience with our response. Your amended study was sent back to the three referees for their re-evaluation, and we have received comments from all of them, which I enclose below. As you will see, the experts stated that the work has been substantially improved by the revisions and they are now broadly in favour of publication.

Thus, we are pleased to inform you that your manuscript has been accepted in principle for publication in The EMBO Journal.

We still need you to take care of a number of minor issues related to formatting and data annotation as detailed below, which should be addressed at re-submission.

Please contact me at any time if you have additional questions related to below points.

As you might remember, every paper at the EMBO Journal now includes a 'Synopsis', displayed on the html and freely accessible to all readers. The synopsis includes a 'model' figure as well as 2-5 one-short-sentence bullet points that summarize the article. I would appreciate if you could provide this figure and the bullet points.

Thank you for giving us the chance to consider your manuscript for The EMBO Journal. I look forward to your final revision.

Again, please contact me at any time if you need any help or have further questions.

Best regards,

Daniel Klimmeck

Daniel Klimmeck PhD
Senior Editor
The EMBO Journal

****

Formatting changes required for the revised version of the manuscript:

>> Adjust the title of the 'Competing Interests' section to 'Disclosure and Competing Interests Statement'.

>>Funding information: please complement our online manuscript system with the complete funding details; currently missing: Indian Institute of Science, UK's national Electron Bio-imaging Centre (eBIC) [proposal EM BI29692-1], funded by the Wellcome Trust, MRC and BBRSC, Bangalore (DBT/PR12422/MED/31/287/2014), DBT-BUILDER program (BT/INF/22/SP22844/2017) and DST-FIST program (SR/FST/LSII-039/2015), Computational support from the high-performance computing facility "Beagle" setup from grants by a partnership between the DBT, India and the Indian Institute of Science (IISc-DBT partnership programme, the DBT-IISc partnership program phase II and the DST-FIST program support.

>> Author Contributions: Remove the author contributions information from the manuscript text. Note that CRediT has replaced the traditional author contributions section as of now because it offers a systematic machine-readable author contributions format that allows for more effective research assessment. and use the free text boxes beneath each contributing author's name to add specific details on the author's contribution.

>> Data not shown: please adjust statement on page 29 - "Error bars are not shown for clarity" by adding complementary error bars.

>> Callouts: add callouts in the main text for individual panels of Appendix Figure S3, S4, S9.

>> Figure files: EV Figures need to be uploaded as Figures (not Expanded View material).

>> Dataset EV legends: Model coordinates and map need to be uploaded as Excel files.

>> Appendix: please adjust the nomenclature in the ToC and file to "Appendix Figure S1,S2..." and "Appendix Table S1,S2...".

>> Data availability section: please make sure to remove PDB data privacy at acceptance.

>> Introduce a separate statistical analysis' section in the Material and methods detailing the algorithms and testing models applied.

>> Consider additional changes and comments from our production team as indicated by the .doc file enclosed and leave changes in track mode.

Further information is available in our Guide For Authors: https://www.embopress.org/page/journal/14602075/authorguide

We realize that it is difficult to revise to a specific deadline. In the interest of protecting the conceptual advance provided by the work, we recommend a revision within 3 months (7th Sep 2023). Please discuss the revision progress ahead of this time with the editor if you require more time to complete the revisions. Use the link below to submit your revision:

https://emboj.msubmit.net/cgi-bin/main.plex

Referee #1:

The authors have addressed all my comments and I have no further criticism. I suggest acceptance of the manuscript in its present form.

Referee #2:

My own concerns as well as those raised by the other two reviewers have been addressed sufficiently. The manuscript has significantly improved after this revision and the conclusions are much clearer now. Specifically, it is helpful that the authors have expanded the discussion regarding potential mechanistic explanations for the essential role of EL7 in sustaining QacA efflux activity, such as a potential contribution to reinforcing the extracellular gate and its presence may prevent leaks during transport. I also appreciate that the authors have added a more detailed description of the MD simulations and regarding the expected structural effects on nanobody/QacA interactions upon transition to other (hypothetical) states of the transport cycle to explain why the presence of the ICabs does not interfere with efflux activity.

Referee #3:

The authors have made a true effort in improving the manuscript, and addressed all the concerns that I raised. I do not have further comments on the manuscript, and congratulate the authors on their work.

All editorial and formatting issues were resolved by the authors.

Dear Dr Aravind Penmatsa,

Thank you for submitting the revised version of your manuscript. I have now evaluated your amended manuscript and concluded that the remaining minor concerns have been sufficiently addressed.

Thus, I am pleased to inform you that your manuscript has been accepted for publication in the EMBO Journal.

Please note that it is EMBO Journal policy for the transcript of the editorial process (containing referee reports and your response letter) to be published as an online supplement to each paper. I would accordingly like to ask for your consent on keeping the additional referee figures included in this file.

Also, in case you might NOT want the transparent process file published at all, you will also need to inform us via email immediately. More information is available here:
https://www.embopress.org/page/journal/14602075/authorguide#transparentprocess

----

Please note that in order to be able to start the production process, our publisher will need and contact you shortly regarding the page charge authorisation and licence to publish forms.

Authors of accepted peer-reviewed original research articles may choose to pay a fee in order for their published article to be made freely accessible to all online immediately upon publication. The EMBO Open fee is fixed at $6,540 USD / £5,310 GBP / €5,900 EUR (+ VAT where applicable), pending application of a waiver which might be possible in this case as discussed.

We offer two licenses for Open Access papers, CC-BY and CC-BY-NC-ND.
For more information on these licenses, please visit:
http://creativecommons.org/licenses/by-nc-nd/3.0/deed.en_US

Should you be planning a Press Release on your article, please get in contact with embojournal@wiley.com as early as possible, in order to coordinate publication and release dates.

On a different note, I would like to alert you that EMBO Press is currently developing a new format for a video-synopsis of work published with us, which essentially is a short, author-generated film explaining the core findings in hand drawings, and, as we believe, can be very useful to increase visibility of the work. This has proven to offer a nice opportunity for exposure i.p. for the first author(s) of the study. Please see the following link for representative examples and their integration into the article web page:
https://www.embopress.org/video_synopses
https://www.embopress.org/doi/full/10.15252/embj.2019103932

Please let me know, should you be interested to engage in commissioning a similar video synopsis for your work. According operation instructions are available and intuitive.

If you have any questions, please do not hesitate to call or email the Editorial Office.

Thank you again for this contribution to The EMBO Journal and congratulations on a successful publication! Please consider us again in the future for your most exciting work.

Kind regards,

Daniel Klimmeck

Daniel Klimmeck, PhD
Senior Editor
The EMBO Journal
EMBO
Postfach 1022-40
Meyerhofstrasse 1

D-69117 Heidelberg
contact@embojournal.org
Submit at: http://emboj.msubmit.net

** Click here to be directed to your login page: https://emboj.msubmit.net

EMBO Press Author Checklist

Corresponding Author Name: Aravind Penmatsa	
Journal Submitted to: EMBO J	
Manuscript Number: EMBOJ-2023-113418	

USEFUL LINKS FOR COMPLETING THIS FORM
The EMBO Journal - Author Guidelines
EMBO Reports - Author Guidelines
Molecular Systems Biology - Author Guidelines
EMBO Molecular Medicine - Author Guidelines

Reporting Checklist for Life Science Articles (updated January
This checklist is adapted from Materials Design Analysis Reporting (MDAR) Checklist for Authors. MDAR establishes a minimum set of requirements in transparent reporting in the life sciences (see Statement of Task: 10.31222/osf.io/9sm4x). Please follow the journal's guidelines in preparing your
Please note that a copy of this checklist will be published alongside your article.

Abridged guidelines for figures
1. Data
The data shown in figures should satisfy the following conditions:
- the data were obtained and processed according to the field's best practice and are presented to reflect the results of the experiments in an accurate and unbiased manner.
- ideally, figure panels should include only measurements that are directly comparable to each other and obtained with the same assay.
- plots include clearly labeled error bars for independent experiments and sample sizes. Unless justified, error bars should not be shown for technical
- if n<5, the individual data points from each experiment should be plotted. Any statistical test employed should be justified.
- Source Data should be included to report the data underlying figures according to the guidelines set out in the authorship guidelines on Data

2. Captions
Each figure caption should contain the following information, for each panel where they are relevant:
- a specification of the experimental system investigated (eg cell line, species name).
- the assay(s) and method(s) used to carry out the reported observations and measurements.
- an explicit mention of the biological and chemical entity(ies) that are being measured.
- an explicit mention of the biological and chemical entity(ies) that are altered/varied/perturbed in a controlled manner.
- the exact sample size (n) for each experimental group/condition, given as a number, not a range;
- a description of the sample collection allowing the reader to understand whether the samples represent technical or biological replicates (including how many animals, litters, cultures, etc.).
- a statement of how many times the experiment shown was independently replicated in the laboratory.
- definitions of statistical methods and measures:
  - common tests, such as t-test (please specify whether paired vs. unpaired), simple χ2 tests, Wilcoxon and Mann-Whitney tests, can be unambiguously identified by name only, but more complex techniques should be described in the methods section;
  - are tests one-sided or two-sided?
  - are there adjustments for multiple comparisons?
  - exact statistical test results, e.g., P values = x but not P values < x;
  - definition of 'center values' as median or average;
  - definition of error bars as s.d. or s.e.m.

Please complete ALL of the questions below.
Select "Not Applicable" only when the requested information is not relevant for your study.

Materials

Newly Created Materials	Information included in the manuscript?	In which section is the information available? (Reagents and Tools Table, Materials and Methods, Figures, Data Availability Section)
New materials and reagents need to be available; do any restrictions apply?	Yes	Purified Icabs generated through this study would be made available upon reasonable request. (included in Data availability)

Antibodies	Information included in the manuscript?	In which section is the information available? (Reagents and Tools Table, Materials and Methods, Figures, Data Availability Section)
For **antibodies** provide the following information:<br>- Commercial antibodies: RRID (if possible) or supplier name, catalogue number and or/clone number<br>- Non-commercial: RRID or citation	Yes	Antibodies generated as part of this study are described in the methods section

DNA and RNA sequences	Information included in the manuscript?	In which section is the information available? (Reagents and Tools Table, Materials and Methods, Figures, Data Availability Section)
**Short novel DNA or RNA including primers, probes**: provide the sequences.	Not Applicable	

Cell materials	Information included in the manuscript?	In which section is the information available? (Reagents and Tools Table, Materials and Methods, Figures, Data Availability Section)
**Cell lines:** Provide species information, strain. Provide accession number in repository **OR** supplier name, catalog number, clone number, and/**OR** RRID.	Not Applicable	
**Primary cultures:** Provide species, strain, sex of origin, genetic modification status.	Not Applicable	
Report if the cell lines were recently **authenticated** (e.g., by STR profiling) and tested for mycoplasma contamination.	Not Applicable	

Experimental animals	Information included in the manuscript?	In which section is the information available? (Reagents and Tools Table, Materials and Methods, Figures, Data Availability Section)
**Laboratory animals or Model organisms:** Provide species, strain, sex, age, genetic modification status. Provide accession number in repository **OR** supplier name, catalog number, clone number, **OR** RRID.	Not Applicable	
**Animal observed in or captured from the field:** Provide species, sex, and age where possible.	Not Applicable	
Please detail **housing and husbandry conditions**.	Not Applicable	

Plants and microbes	Information included in the manuscript?	In which section is the information available? (Reagents and Tools Table, Materials and Methods, Figures, Data Availability Section)
**Plants:** provide species and strain, ecotype and cultivar where relevant, unique accession number if available, and source (including location for collected wild specimens).	Not Applicable	
**Microbes:** provide species and strain, unique accession number if available, and source.	Yes	E.coli LMG194 (ΔlacX74 galE thi rpsL ΔphoA (Pvu II) Δara714 leu::Tn10)ΔmdfA ΔacrB ΔydhE::Kan (details included in methods section; derived from Majumder etal., 2019, J. Mol. Biol)

Human research participants	Information included in the manuscript?	In which section is the information available? (Reagents and Tools Table, Materials and Methods, Figures, Data Availability Section)
If collected and within the bounds of privacy constraints report on age, sex and gender or ethnicity for all study participants.	Not Applicable	

Core facilities	Information included in the manuscript?	In which section is the information available? (Reagents and Tools Table, Materials and Methods, Figures, Data Availability Section)
If your work benefited from core facilities, was their service mentioned in the acknowledgments section?	Yes	Acknowledgements carry information on core facilities

Design

Study protocol	Information included in the manuscript?	In which section is the information available? (Reagents and Tools Table, Materials and Methods, Figures, Data Availability Section)
If study protocol has been **pre-registered, provide DOI in the manuscript**. For clinical trials, provide the trial registration number **OR** cite DOI.	Not Applicable	
Report the **clinical trial registration number** (at ClinicalTrials.gov or equivalent), where applicable.	Not Applicable	

Laboratory protocol	Information included in the manuscript?	In which section is the information available? (Reagents and Tools Table, Materials and Methods, Figures, Data Availability Section)
Provide DOI OR other citation details if **external detailed step-by-step protocols** are available.	Not Applicable	

Experimental study design and statistics	Information included in the manuscript?	In which section is the information available? (Reagents and Tools Table, Materials and Methods, Figures, Data Availability Section)
Include a statement about **sample size** estimate even if no statistical methods were used.	Yes	Information on statistics are included in relevant figure legends for example Fig .EV4
Were any steps taken to minimize the effects of subjective bias when allocating animals/samples to treatment (e.g. **randomization procedure**)? If yes, have they been described?	Not Applicable	
Include a statement about **blinding** even if no blinding was done.	Not Applicable	
Describe **inclusion/exclusion criteria** if samples or animals were excluded from the analysis. Were the criteria pre-established?<br><br>If sample or data points were omitted from analysis, report if this was due to attrition or intentional exclusion and provide justification.	Not Applicable	
For every figure, are **statistical tests** justified as appropriate? Do the data meet the assumptions of the tests (e.g., normal distribution)? Describe any methods used to assess it. Is there an estimate of variation within each group of data? Is the variance similar between the groups that are being statistically compared?	Yes	Applicable only to the analyses performed for Figur EV4c. Siginifcance values calculated for each sample comparison in the histogram with p-value reported in the legend using unpaired t-tests for biological replicates.

Sample definition and in-laboratory replication	Information included in the manuscript?	In which section is the information available? (Reagents and Tools Table, Materials and Methods, Figures, Data Availability Section)
In the figure legends: state number of times the experiment was **replicated** in laboratory.	Yes	Figure legends indicate number replicates used for the data
In the figure legends: define whether data describe **technical or biological replicates**.	Yes	Figure legends indicate number technical and biological replicates used for the data

Ethics

Ethics	Information included in the manuscript?	In which section is the information available? (Reagents and Tools Table, Materials and Methods, Figures, Data Availability Section)
Studies involving **human participants**: State details of **authority granting ethics approval** (IRB or equivalent committee(s), provide reference number for approval.	Not Applicable	
Studies involving **human participants**: Include a statement confirming that **informed consent** was obtained from all subjects and that the experiments conformed to the principles set out in the WMA Declaration of Helsinki and the Department of Health and Human Services Belmont Report.	Not Applicable	
Studies involving **human participants:** For publication of **patient photos**, include a statement confirming that consent to publish was obtained.	Not Applicable	
Studies involving experimental **animals**: State details of **authority granting ethics approval** (IRB or equivalent committee(s), provide reference number for approval. Include a statement of compliance with ethical regulations.	Yes	Approvals for immunization of Indian camels are available from NRCC, Bikaner animal ethics committee (NRCC/PME/6(141)/2000-Tech-VolII) and along with CPCSEA clearance (25/19/2018-CPCSEA) for the study. Relevant documents can be made available if required.
Studies involving **specimen and field samples:** State if relevant **permits** obtained, provide details of authority approving study; if none were required, explain why.	Not Applicable	

Dual Use Research of Concern (DURC)	Information included in the manuscript?	In which section is the information available? (Reagents and Tools Table, Materials and Methods, Figures, Data Availability Section)
Could your study fall under dual use research restrictions? Please check biosecurity documents and list of **select agents and toxins** (CDC): https://www.selectagents.gov/sat/list.htm	Not Applicable	
If you used a select agent, is the security level of the lab appropriate and reported in the manuscript?	Not Applicable	
If a study is subject to dual use research of concern regulations, is the name of the **authority granting approval and reference number** for the regulatory approval provided in the manuscript?	Not Applicable	

Reporting

The MDAR framework recommends adoption of discipline-specific guidelines, established and endorsed through community initiatives. Journals have their own policy about requiring specific guidelines and recommendations to complement MDAR.

Adherence to community standards	Information included in the manuscript?	In which section is the information available? (Reagents and Tools Table, Materials and Methods, Figures, Data Availability Section)
State if relevant guidelines or checklists (e.g., **ICMJE, MIBBI, ARRIVE, PRISMA**) have been followed or provided.	Not Applicable	
For **tumor marker prognostic studies,** we recommend that you follow the **REMARK** reporting guidelines (see link list at top right). See author guidelines, under 'Reporting Guidelines'. Please confirm you have followed these guidelines.	Not Applicable	
For **phase II and III randomized controlled trials**, please refer to the **CONSORT** flow diagram (see link list at top right) and submit the CONSORT checklist (see link list at top right) with your submission. See author guidelines, under 'Reporting Guidelines'. Please confirm you have submitted this list.	Not Applicable	

Data Availability

Data availability	Information included in the manuscript?	In which section is the information available? (Reagents and Tools Table, Materials and Methods, Figures, Data Availability Section)
Have **primary datasets** been deposited according to the journal's guidelines (see 'Data Deposition' section) and the respective accession numbers provided in the Data Availability Section?	Yes	Coordinates and maps deposited in the PDB and EMDB respectively and included in the data availability section.
Were **human clinical and genomic datasets** deposited in a public access-controlled repository in accordance to ethical obligations to the patients and to the applicable consent agreement?	Not Applicable	
Are **computational models** that are central and integral to a study available without restrictions in a machine-readable form? Were the relevant accession numbers or links provided?	Yes	Information is provided as supplemental information and will be uploaded to a shared resource upon acceptance.
If publicly available data were reused, provide the respective **data citations in the reference list**.	Not Applicable	
